# *Post-hoc* permutation GWAS refinement reveals robust marker-trait associations in faba bean

**DOI:** 10.3389/fpls.2026.1895808

**Published:** 2026-08-10

**Authors:** Troels W. Mouritzen, Elesandro Bornhofen, Thilani Jayakody, Cathrine Kiel Skovbjerg, Linda Kærgaard Nielsen, Andrea Schiemann, Jens Christen Nørgaard Knudsen, Winnie S. Füchtbauer, Jihad Orabi, Luc Janss, Stig U. Andersen

**Affiliations:** 1 Plant Molecular Biology, Institute for Molecular Biology and Genetics, Aarhus University, Aarhus, Denmark; 2 Center for Quantitative Genetics and Genomics, Aarhus University, Aarhus, Denmark; 3 Nordic Seed, Odder, Denmark; 4 Sejet Plant Breeding, Horsens, Denmark

**Keywords:** agronomic traits, diversity panel, faba bean, genome-wide association study, marker-assisted selection, multi-environment trials, permutation, quantitative trait loci

## Abstract

**Introduction:**

Faba bean (*Vicia faba* L.) is a high-yielding, protein-rich grain legume with considerable potential for sustainable cropping systems. However, its genetic improvement has been constrained by a large, complex genome and historically limited genomic resources. We aimed to identify agronomically relevant QTLs in a diverse faba bean panel using genome-wide associations studies (GWAS) with rigorous control of false positives due to model violations.

**Methods:**

The NORFAB faba bean diversity panel was evaluated for agronomic traits over four years (2018–2021) at two locations in Denmark. GWAS were conducted using three complementary methods, incorporating a standard predictor-permutation test, novel in a GWAS context, for the top associations post-hoc to rigorously control false positives due to model violations.

**Results:**

This approach identified 13 QTLs associated with nine agronomic traits. Several QTLs colocalized with genes previously associated with similar traits, such as the flowering time regulator *FLOWERING LOCUS T*. Incorporating a permutation step consistently discovered more internally consistent QTLs compared to using only parametric *p*-values, with each QTL detected by more GWAS methods and associated with a greater number of SNPs, traits and trials.

**Discussion:**

Our results add interesting QTLs and biologically plausible candidate genes for multiple agronomic traits for incorporation into the faba bean molecular breeding toolkit and suggest that SNP-level post-hoc permutation testing can improve GWAS reliability.

## 1 Introduction

Faba bean (*Vicia faba* L.) is a globally grown grain legume, cultivated on all continents except Antarctica (Warsame et al., 2018). It is high-yielding, with a yield potential of up to 10 *tha*^−1^, among the highest of all grain legumes (Cernay et al., 2016). It is also high in protein, with an average value of around 29% (Raikos et al., 2014; Chávez-Murillo et al., 2018). With the growing global emphasis on sustainable and domestic macronutrient production, the unique combination of high yield and protein content has renewed interest in faba bean as a key crop for food security and plant-based food supply (Dhull et al., 2022). Furthermore, it is a particularly efficient and generous nitrogen fixer, which leaves behind more nitrogen in the ground than it takes up, making it a prime candidate for incorporating into diversified crop rotations or intercropping (Jensen et al., 2010; Köpke and Nemecek, 2010).

Despite these advantages, the genetic improvement of faba bean has lagged behind other major crops. This is in part due to its large genome size of 13 Gb, one of the largest among diploid field crops, and the absence of a wild progenitor, limiting the available genetic diversity for breeding (Jayakodi et al., 2023).

Recent advances, notably the publication of the faba bean genome and the development of a high-density genotyping platform, have transformed the landscape for molecular breeding approaches (Jayakodi et al., 2023). These resources empower breeders to implement modern breeding techniques, such as genomic selection, marker-assisted selection, genome editing, GWAS (Iqbal et al., 2025). Since the introduction of a reference genome in faba, the number of GWAS published on faba bean has risen rapidly (Mouritzen et al., 2025). There have even been a few publications on genomic prediction in faba bean (Bornhofen et al., 2024; Abou Khater et al., 2025; Lippolis et al., 2025), accelerating the transition from traditional breeding to genomics-enabled improvement strategies further.

With all previous QTLs and GWAS findings now re-anchored onto the latest reference genome (Mouritzen et al., 2025), this has facilitated robustness in faba bean GWAS through easy multi-study comparisons. However, enabling multi-study comparisons also revealed that many GWAS associations are not replicated across studies in faba bean (Mouritzen et al., 2025). This is a general problem, with non-reproducible associations plaguing plant GWAS (Burghardt et al., 2017; Clauw et al., 2025). This may reflect differences in statistical power, marker sets (especially in marker density) genetic panels, and/or environments. Environmental and physiological factors, including nutrient-mediated susceptibility effects, have been shown to significantly influence trait expression and genetic associations (Liu et al., 2026).

However, some signals are also likely to be false positives resulting from the statistical GWAS framework itself, arising from the winner’s curse effect. This effect alludes to the largest estimated effects of many tested effects tending to be systematically larger in magnitude, as there is always a noise component to this estimate, biasing them upwards (Beavis and Wilkinson, 1994; Xu, 2003; Xie et al., 2021). This observation underscores the need for more robust GWAS methods, particularly in crops with limited resources where very large panels and extensive field validation are unrealistic.

In this study, we characterized 196 faba bean accessions from the NORFAB diversity panel (Skovbjerg et al., 2023) in a multi-location, multi-year phenotyping trial across two locations in Denmark. All accessions were previously genotyped with SPET technology (Jayakodi et al., 2023), allowing the identification of 465,259 single nucleotide polymorphisms (SNPs). Utilizing these high-density datasets, we conducted GWAS integrating a *post-hoc* step consisting of a standard permutation-based test to control for false positives due to model violations. We aimed to identify trait-associated genomic regions with sufficient precision to facilitate candidate gene discovery and to assess whether permutation-derived *p*-values reduce spurious associations and improve association robustness compared to standard methods.

## 2 Materials and methods

### 2.1 Germplasms

The NORFAB panel used in this study contains 196 accessions. The selected accessions were based on maximizing trait diversity, geographical origin, and resistance to northern Europe pathogens. The accessions had a wide geographical distribution, including accessions from north and central Europe, the middle east, and the Indian subcontinent. The material was obtained from donations from universities, seed companies and genebanks and included both cultivar-derived lines and more exotic landrace-type accessions. While some of the lines were found to be sufficiently inbred for direct entry into the panel, others were put through an inbreeding schema prior to inclusion. A full description of the germplasm can be found in Skovbjerg et al., 2023.

### 2.2 DNA extraction, genotyping and filtering

DNA extraction and genotyping was previously described in Jayakodi et al., 2023. The raw reads were trimmed with cutadapt v1.15 (Martin, 2011) and aligned to the Hedin/2 v2 assembly (Jayakodi et al., 2023; Zhang et al., 2026) using bwa mem v0.7.17 (Li, 2013) and genotypes were called using bcftools mpileup v1.8 (Danecek et al., 2021). The variants included were then filtered according to the criteria: Variant quality > 50, Mapping quality > 50, Heterozygous proportion of minor allele< 0.35, Fraction of missing genotypes< 0.2, and a minor allele count of 10. Missing genotypes were imputed using Beagle 5.3 (Browning et al., 2018), with the burnin parameter set to 10, and the iterations parameter set to 100. Chromosome 1 was split up into two parts to facilitate data analysis.

### 2.3 Population structure

Population structure was assessed with ADMIXTURE version 1.3.0 (Alexander et al., 2009). This was done using only variants with a minor allele frequency (MAF) of more than 0.05, which was then further pruned by Plink v2.00a5LM (Purcell et al., 2007) using the indep-pairwise functionality, using a window size of 50 kb, step size of 10 kb, and a *r*^2^ threshold of 0.5. 10 cross-validation folds were used in ADMIXTURE to assess each amount of chosen k from 2 to 20. Population structure was also assessed with principal component analysis, using only variants with MAF greater than 0.05, with the prcomp R function.

### 2.4 Field trials

NORFAB field trials were conducted over two locations in Denmark, at Nordic Seed (55° 57′ 39.4″ N, 10° 14′ 56.9″ E) and Sejet Plant Breeding (55° 49′ 27.7″ N, 9° 56′ 37.4″ E). The trials were conducted from 2018 to 2021 at Sejet Plant Breeding, and 2018 to 2020 at Nordic Seed. Field designs followed a randomized block design, with three blocks per field. Each plot consisted of three rows. Plot sizes and seeds per plot varied across years and locations. All trials were rainfed and received standard treatments of fertilizer, insecticide, herbicide and adjuvant. Sowing occurred at varying times in April, and harvests were carried out at varying times in August to September, depending on local conditions.

Plants were scored for a range of yield-related, morphological, and biotic stress related traits. In this report, we focus on the following traits: Earliness of flowering, end of flowering, duration of flowering, plant height, seed circularity, seed length-to-width ratio, maturation date, branching, sterile tillers, lodging, number of ovules, and internode length. Not all traits were scored in every year, and not all accessions were sown in all location-year combinations. Branching, internode length, ovules, and sterile tillers were recorded as counts; flowering and maturation traits were measured in days; plant height in centimeters; and lodging and seed circularity as an index. From earliness of flowering and end flowering, the duration of flowering was calculated.

### 2.5 Variance components and heritability

Variance components were estimated using lme4breeding v1.0.90 (Bates et al., 2023; Covarrubias-Pazaran, 2025) in R using the model:


y=Tτ+Bβ+Zu+Px+ϵ

Where **y** is the phenotype vector, **τ** is the vector of trial effects, **β** is the vector of block effects nested within trial, **u** is the vector of genotype effects, and **x** is the vector of genotype and trial interaction effects. For this purpose, a trial was taken as each unique combination of year and location. **T**,**B**,**Z**,**P** are design matrices. **τ** was estimated as a fixed effect, while **u**, **x** and **β** were estimated as random effects with:


$$u∼N\left(0,\sigma_{g}^{2}\cdot K\right),\;x∼N\left(0,\sigma_{gxe}^{2}\cdot I\right),\;\beta \;∼\;N\left(0,\;\sigma_{b}^{2}\cdot I\right)$$

With **K** being the genomic relationship matrix and **I** being an identity matrix, and $\sigma_{g}^{2}$, $\sigma_{gxe}^{2}$ and $\sigma_{b}^{2}$ being the additive genetic, genotype-by-environment (GxE) and block variance, respectively, all estimated from the data. **K** was constructed as:


$$K=m\frac{GG^{\prime }}{tr\left(GG^{\prime }\right)}$$

With **G** being the centered and scaled SNP matrix, m being the number of SNPs, and *tr*(·) denoting the trace operator.

For traits scored in a single trial GxE and trial effects were not included.

Statistical significance of each of the effects were determined. Testing significance of fixed effects was done by fitting a reduced model without the effect and any higher order interactions and testing this reduced model against the full model with the anova (Chambers and Hastie, 2017) function in R v4.3.2 (R Core Team, 2023). Statistical significance of random effects or fixed effects part of higher order interactions modelled as random effects was done by permutation. This is because chi-squared approximation of the likelihood ratio test score does not hold when you are testing at the boundary of the parameter space, i.e. whether the variance of the random effect is equal to zero. For each random effect, a reduced model not containing the effect and any higher order interactions was fitted, and the likelihood ratio test score was determined between the full model and the reduced model. Then the phenotype vector was simulated 1000 times under the reduced model. For each simulation, the full model and the reduced model were fitted to the data, and a likelihood ratio score was determined. The *p*-value was then determined as $p=\frac{s+1}{n}$, with s being the number of likelihood ratio test scores larger than the observed, and n being the number of simulations.

This was done in a stepwise fashion, starting from the full model described above, each time removing the least significant effect with a significance level above 0.01, stopping when no effects were non-significant above this level. The significance threshold was chosen to be at 0.01 to go for a simpler, more parsimonious model, especially when the p-value was close to the threshold. Higher-order interactions were also removed if their constituent effects were non-significant. Variance contributions of the random effects in the model were extracted using the VarCorr function from the lme4 package (Bates et al., 2015). To estimate the variance contribution of the fixed effects, we calculated the variance of the fitted values associated with each fixed effect. Specifically, for each fixed effect, we constructed a fitted value vector by multiplying the relevant columns of the fixed-effects design matrix by their estimated coefficients, then computed the variance of these vectors separately.

Best linear unbiased predictors (BLUP) were extracted from this best fitting model. Narrow-sense heritabilities were determined on an entry-mean basis for each trait using Cullis’ method (Cullis et al., 2006) to account for the unbalanced design, using the equation:


$$H^{2}=1-\frac{{\bar{v}}_{\text{ΔBLUP}}}{2\cdot \sigma_{g}^{2}}$$

With $\sigma_{g}^{2}$ being the genetic variance, and ${\bar{v}}_{\text{ΔBLUP}}$ being the average standard error of the BLUPs. Both were extracted from the model described above.

Heritability standard errors were estimated using a bootstrap procedure. For each trait, 100 datasets were simulated under the best-fitting model using the simulate function in R. Each dataset was refitted to the model, and heritability was re-estimated. The standard error was calculated as the standard deviation of the resulting distribution of heritability estimates.

A similar procedure and model was utilized to estimate the best linear unbiased predictors (BLUE) for use in GWAS, as recommended in Holland and Piepho, 2024. The two differences being the genetic effect fitted as a fixed effect, and using lme4 1.1_37 (Bates et al., 2015, Bates et al., 2023) or the *lm* (Wilkinson and Rogers, 1973; Chambers, 1992) base R function, depending on whether the model fitted contained a random effect or not, respectively.

### 2.6 GWAS and permutation

GWAS was run using GEMMA 0.98.5 (Zhou and Stephens, 2012), ISIS EM-BLASSO (Tamba et al., 2017) using the ISIS function in mrMLM 5.0.1 (Zhang et al., 2022) R package and FARMCPU (Liu et al., 2016) using FarmCPUpp (Kusmec and Schnable, 2018). GWAS was run both on single trial values and BLUEs of lines for multi trial traits, with each trial being a unique combination of year and location. Single trial values were averaged across replicates, and BLUEs of lines were calculated as stated above, and only on multi trial traits. Only alleles with a MAF of 0.05 were included in the analysis. Number of effective tests were calculated for each run, that is for each combination of trait and trial, by utilizing the simpleM (Gao et al., 2008) method in a custom R script, setting the number of SNPs per window equal to the number of lines in the particular trait-trial combination. This was then transformed into a p-value threshold using the Šidák (Šidák, 1967) correction.

For all associations with *p* < 10^−4^ for any of the GWAS methods, we performed GEMMA permutation GWAS. Specifically, we calculated the kinship matrix with GEMMA with all SNPs unpermuted, with SNPs with MAF below 0.05 excluded on a trait-trial basis. Then for each SNP association, we permuted the SNP and associated it with the particular trait-trial combination it was associated with up to 1 billion times, stopping prematurely if we found 100 or more associations stronger than the unpermuted one. This was done to avoid further calculations on associations that were not significant. This effectively fixes our value k, the amount of more extreme values observed, to be based on a negative binomial distribution when it is stopped prematurely, and a binomial distribution when it allowed to run to completion. *P*-values are then estimated as $\hat{p}=\frac{k+1}{n+1}\;$, with n being the number of times the permutation was allowed to run. This also fixes the minimum obtainable *p*-value to be equal to $p_{min}=\frac{0+1}{10^{9}+1}\approx 10^{-9}$. No principal components were incorporated into the GWAS analysis. Population structure was instead accounted for using a genomic relationship matrix, which has been shown to adequately capture relatedness among individuals. In this context, the inclusion of principal components in addition to a genomic relationship matrix may be redundant, or even reduce model performance compared to models relying solely on the genomic relationship matrix (Liu et al., 2011; Yao and Ochoa, 2023). Lastly, although FarmCPU and ISIS EM-BLASSO do not explicitly include a full genomic relationship matrix, FarmCPU accounts for population structure with a relationship matrix built from a subset of associated markers (Liu et al., 2016), and ISIS EM-BLASSO does it through a Bayesian LASSO framework on top associated markers (Tamba et al., 2017).

### 2.7 Clumping, QTL genomic span and candidate genes

Associations were grouped by taking the set of permutation-significant associations. These associations were then ordered according to the frequency with which each SNP appeared in the permutation-significant set. Starting with the SNP (lead SNP) that appeared most frequently, all SNPs within a 1 Mb window that were permutation-significant for any trait, or that had an initial, unpermuted *p*-value less than the previously determined threshold, were considered. *R*^2^ values between the lead SNP and all considered SNPs were calculated. SNPs were assigned to the same QTL if they showed high linkage disequilibrium (LD) (*R*^2^ ≥ 0.8). SNPs already assigned to a QTL were excluded from subsequent grouping. *R*^2^ scores were calculated using the R package snpStats v.1.58.0 (Clayton, 2025).

### 2.8 Candidate follow-up

For each QTL, a region was defined as encompassing all SNPs within 5 Mb upstream and downstream of the QTLs candidate SNPs to capture all possible associated SNPs. Pairwise LD values (*R*^2^) between all regional SNPs and the candidate SNPs were calculated using snpStats (Clayton, 2025), and any SNP exhibiting *R*^2^ ≥ 0.5 with at least one candidate SNP were considered to be in medium strong LD. SNP consequences were annotated using the software SnpEff v.4.3t (Cingolani et al., 2012) based on gene annotations and a reference genome (v2) previously described in Jayakodi et al., 2023 and Zhang et al., 2026.

## 3 Results

### 3.1 The NORFAB project

**Figure 1:**
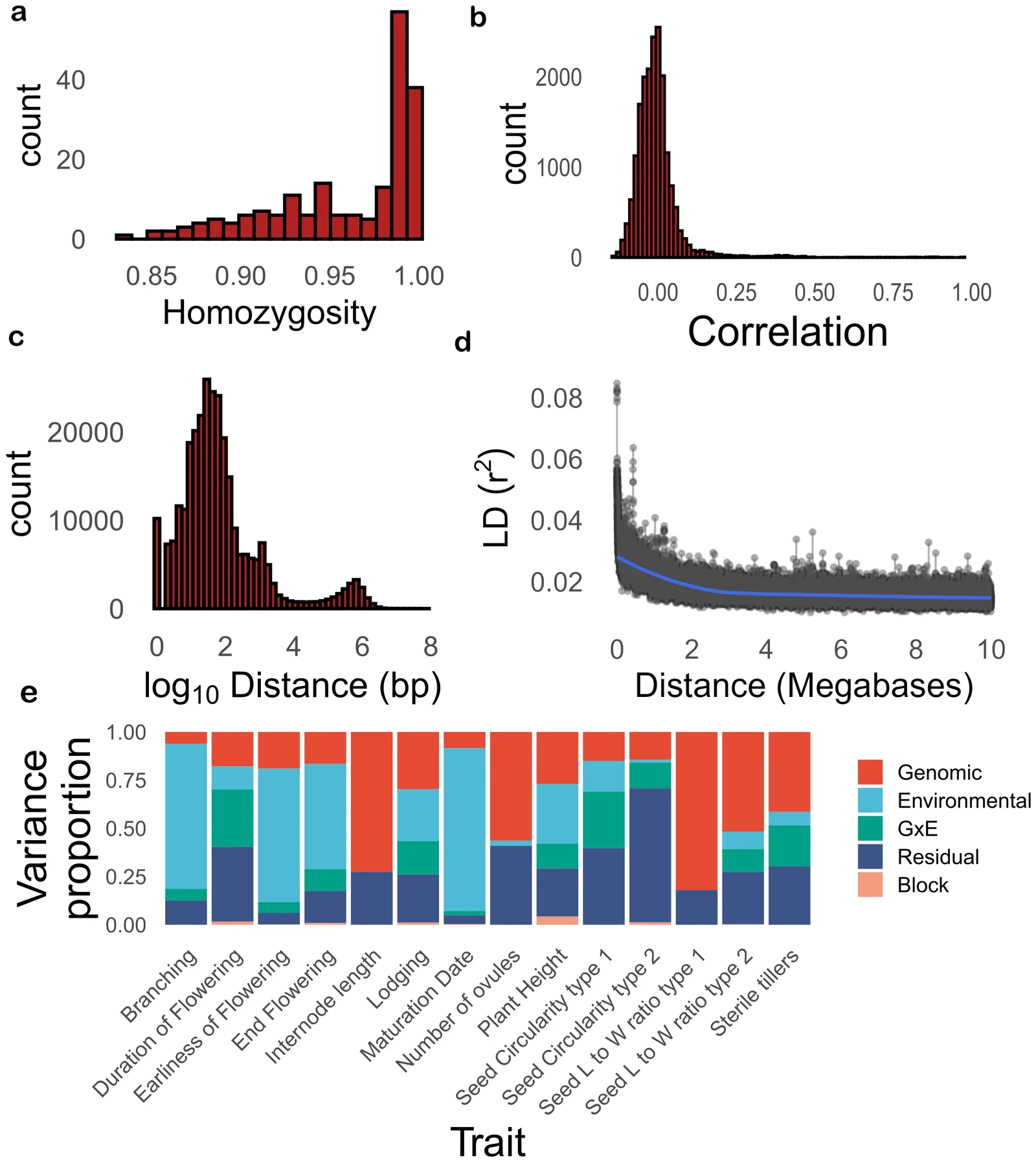
Panel analysis and variance composition. **(A)** Histogram of rates of homozygous calls for all genotypes. **(B)** Genotypic correlations between all genotypes. **(C)** Histogram of log10 distances between all adjacent SNPs. Each bin contains the summary count of all SNP to adjacent SNP distances at a particular distance, shown on the x axis as log10 distances. **(D)** Average LD decay across all panels and chromosomes, as a function of distance between SNPs. Each point is 1000 bp apart, with its y position indicating the average r^2^ value between SNPs separated by the distance on the x-axis. The blue line indicates a smoothed spline fitted to the data. **(E)** Additive variance composition of all traits. Variance is divided into 5 groups, genetic, environmental, GxE, block within fields, and the residual variance. Be aware that Internode Length and Seed L to W ratio type 1 were only scored in a single environment.

The NORFAB panel consists of 196 accessions with a wide distribution of geographical origins (Skovbjerg et al., 2023). Accessions were previously genotyped with the 90K probe set using single primer enrichment technology (SPET) (Jayakodi et al., 2023). After mapping, calling, filtering and imputation, this resulted in a total of 465,259 SNPs. Analysis of homozygosity rates show that the accessions are highly homozygous, with a median and mean rate of homozygosity of 0.98 and 0.96 respectively, and the least homozygous line having a homozygosity rate of 0.83 (**Figure 1A**; **Supplementary file 1** Homozygosity). We assessed inter-accession correlation by extracting covariances from the genetic covariance matrix and converting them to Pearson correlation coefficients. While most lines show close to no relation to each other, a few lines do show high reciprocal relatedness (**Figure 1B**; **Supplementary file 1** Covariances). SNP density reflects the clustered nature of genotypes given a probe-based method, with a median and mean distance between adjacent SNPs of 40 bp and 40 kb, respectively (**Figure 1C**; **Supplementary file 1** Variants). The big difference between median and mean also reflects the long stretches of repetitive genomic regions in faba bean. LD decayed rapidly, with average *R*^2^ within 1 kb windows decaying to half within 17 kb on average, and LD returning to baseline levels at approximately 2 Mb (**Figure 1D**; **Supplementary file 1** LD). The population structure was analyzed with principal component analysis and ADMIXTURE (Alexander et al., 2009). The three admixture components that were found in Skovbjerg et al., 2023 were found to be well-fitting (**Supplementary file 1** ADMIXTURE), with the three admixture components corresponding well to the first two principal components (**Extended data figure 1**).

Accessions were phenotyped in seven different trials at two locations in Denmark, 2018–2020 at Nordic Seed and 2018–2021 at Sejet Plant Breeding. The trials were scored for 11 traits. Seed circularity and seed length-to-width ratio were scored in different ways across different years, and it was decided to split these into separate traits. Not all traits were scored in all trials, and the number of lines and datapoints varies widely between the different traits and trials (**Table 1**; For field trial data, see **Supplementary file 2**).

**Table 1:** Trait heritabilities and observations.

		Nordic Seed			Sejet			
Trait	Heritability	2018	2019	2020	2018	2019	2020	2021
Branching	0.6 (0.055)	329 (88)	450 (119)	631 (205)		328 (110)		
Duration of Flowering	0.503 (0.097)	173 (87)	351 (118)	587 (205)				
Earliness of Flowering	0.893 (0.012)	330 (88)	473 (118)	634 (205)	290 (89)	366 (112)		630 (200)
End Flowering	0.673 (0.064)	325 (87)	473 (118)	636 (205)				
Internode length	0.895 (0.02)		450 (119)					
Lodging	0.766 (0.037)		949 (119)	636 (205)				630 (200)
Maturation Date	0.823 (0.027)	316 (88)	471 (118)	634 (205)		328 (110)		
Number of ovules	0.859 (0.045)		447 (119)	631 (205)				
Plant Height	0.81 (0.026)	330 (88)	473 (118)	634 (205)		366 (112)		630 (200)
Seed Circularity type 1	0.408 (0.13)	330 (88)		588 (199)				
Seed Circularity type 2	0.504 (0.11)						660 (209)	627 (200)
Seed L to W ratio type 1	0.935 (0.013)					326 (110)		
Seed L to W ratio type 2	0.891 (0.013)	330 (88)		588 (199)			660 (209)	627 (200)
Sterile tillers	0.696 (0.099)		450 (119)	631 (205)				

Table listing entry-mean heritabilities calculated using Cullis’ method for all traits across environments and how many total observations across the two environments (Nordic Seed and Sejet) across all tested years (2018-2021). For the heritability column, the estimated heritability is given outside the parentheses while the estimated standard error is given inside the parentheses. The number of observations is split into two numbers, the number of total observations outside the parentheses, and the number of genotypes within the parentheses. Empty cells indicate that was not scored that particular year.

### 3.2 Phenotype analysis

To estimate the effects of genotype, trial, replicate and GxE effects, a linear mixed model was fitted separately for each trait, finding the best fitting model for each trait using backwards selection (see Methods). From this best fitting model, variance components were estimated. Genetic variance explained between 82% (seed L to W ratio type 1) and 6% (branching), with most traits falling within 10-30% (**Figure 1E**; **Supplementary file 1** Variance composition). In most multi-trial traits, the environment exerted the greatest influence on phenotypic variation. Narrow-sense heritabilities were also estimated on an entry-mean basis in this model for each trait using Cullis’ method, and were between 0.4 and 0.94, with most traits falling between 0.6 and 0.9 (**Table 1**; **Supplementary file 1** Variance composition).

To complement the previous model used for estimating heritability and variance components, a best fit model with a fixed effect of genotypes was selected and used to generate BLUEs for all traits assessed across multiple environments (**Supplementary file 1** BLUEs). As expected, many related traits show strong correlations among their BLUE values, including end flowering, maturation date, and earliness of flowering (**Extended data figure 2**). Similarly, seed length-to-width ratio type 1 and 2 are correlated with each other and with seed circularity type 1. In contrast, seed circularity type 2 is not correlated with these traits and is generally weakly correlated with other measured traits. In addition, there are other notable absences of stronger correlations, such as between lodging and plant height, and duration of flowering with end of flowering or maturation date. Nevertheless, most traits display strong correlation with at least one other trait, for instance sterile tillers with branching and plant height with internode length, which underscores their genetic determination.

### 3.3 GWAS

GWAS was run on all traits using three models, FarmCPU, GEMMA, and ISIS EM-BLASSO. It was run on BLUEs of lines as well as single environment trait values for all traits. A minimum MAF of 0.05 was set for each run. The significance threshold was determined by calculating the effective number of tests for each run using the simpleM (Gao et al., 2008) method (**Supplementary file 1** Thresholds). The thresholds exhibited only small variations, with values spanning from *p* ≤ 3.06·10^−7^ to *p* ≤ 3.28·10^−7^. To better control for genomic inflation due to violation of assumptions of the statistical models, we extracted all 8544 associations with a *p*-value<10^−4^ and recomputed *p*-values with a permutation approach using GEMMA (**Supplementary file 1** GWAS results & Permutation results). The *p*-value cutoff of 10^−4^ was selected as it was ~1000 fold lower than the simpleM threshold, making it unlikely that associations below this thresholds would reach genome-wide significance after permutation testing. All significant hits were then grouped. This was done by first ranking SNPs according to the number of times they were associated with the trait at a significance threshold of *p* < 10^−4^. We then grouped SNPs into QTLs in this order if within a conservative window of 1 Mb and they showed high LD (*R*^2^ ≥ 0.8). Associations significant in the original GWAS but not in the permutation step were allowed to be grouped onto a QTL where there already was a permutation-significant association.

A total of 31 associations were identified, corresponding to 21 unique SNPs located within 13 QTLs (**Table 2**; **Supplementary file 1** Permutation GWAS results). No hits were observed for seed circularity (type 1), length to width ratio (both types), number of ovules, or internode length, and neither for the trial at Sejet in 2018. The identified associations spanned nine traits across six individual trials as well as the BLUE summary estimates. Of these 13 QTLs, two were linked to more than one trait. Many QTLs exhibited signals across multiple trials or using different GWAS methods, emphasizing both the robustness and sensitivity of the GWAS approach used. Notably, all QTLs demonstrated some degree of association when analyzed with GEMMA. This pattern likely reflects the underlying design of the permutation-based filtering procedure, which employed GEMMA for *p*-value simulation; consequently, any locus identified as significant under the permutation framework would be expected to exhibit a corresponding signal in the parametric GEMMA GWAS.

**Table 2:** Significant QTLs from post-hoc permutation.

QTL	Chromosome	Position	MAF	Trait	Trial	Method	P.value	Permutation P.value	Slope	Variance Explained
1	chr1_partA	17606861 17607261 17608720 17934798 18368903 18369048	0.41-0.481	Sterile Tillers End Flowering	BLUEs across trials Nordic Seed 2020 Nordic Seed 2018	GEMMA FarmCPU	3.13e-12 - 2.88e-07	2e-08-1.87e-05	0.0663-1.46	24.8-46.3
2	chr1_partA	318637909	0.125	Maturation Date	Nordic Seed 2020	FarmCPU ISIS EM-BLASSO GEMMA	5.74e-14 - 2.56e-07	1.40e-07	1.34-2.88	15.3-29.9
3	chr1_partA	350471259	0.078-0.0995	Lodging	BLUEs across trials Nordic Seed 2020 Nordic Seed 2019	FarmCPU ISIS EM-BLASSO GEMMA	1.9e-19 - 6.49e-08	3.09e-07-1.16e-4	0.66-1.14	12.3-24.3
4	chr1_partB	951256330	0.184-0.188	End Flowering	Nordic Seed 2020 BLUEs across trials	FarmCPU ISIS EM-BLASSO GEMMA	8.05e-23 - 2.54e-07	2.2e-07-2.9e-06	0.989-2.06	10.7-16.6
5	chr2	979322197 979322200	0.342	Seed Circularity type 2	Sejet 2020	FarmCPU GEMMA	2.82e-11 - 2.83e-08	4.7e-08-5e-08	0.0702-0.102	19.3-23.7
6	chr3	187881138 187881139	0.096-0.0964	Lodging	Sejet 2021	FarmCPU ISIS EM-BLASSO GEMMA	1.26e-12 - 1.91e-05	2.86e-07-2.94e-07	0.372-1.36	7.51-19.9
7	chr3	211434134	0.389	End Flowering	Nordic Seed 2018	GEMMA	1.34e-06	1.87e-07	2.08	34.7
8	chr3	546790466	0.093	End Flowering	Nordic Seed 2018	GEMMA	2.70e-08	3.19e-07	3.8	10.3
9	chr3	1189751296	0.125	Earliness of Flowering	Nordic Seed 2020	FarmCPU GEMMA	2.15e-07 - 1.24e-06	1.18e-07	1.27-2.69	11.2-20.8
10	chr3	1593291732	0.47-0.475	Maturation Date End Flowering	Nordic Seed 2018	FarmCPU ISIS EM-BLASSO GEMMA	1.39e-11 - 1.34e-06	1.61e-07-2.57e-4	0.578-2.01	10.2-30.2
11	chr5	357034768	0.128	Duration of Flowering	Nordic Seed 2019	FarmCPU ISIS EM-BLASSO GEMMA	9.74e-11 - 3.76e-07	1.84e-07	1.74-3.21	15.2-18.8
12	chr5	535097234	0.266-0.273	Plant Height	BLUEs across trials Sejet 2019	ISIS EM-BLASSO GEMMA	1.5e-09 - 2.01e-07	9.9e-08-2.12e-4	2.84-5.91	22.7-32.4
13	chr5	631619013 631619046	0.0944-0.101	Earliness of Flowering	Nordic Seed 2020 BLUEs across trials	FarmCPU ISIS EM-BLASSO GEMMA	3.56e-10 - 4.55e-06	1.75e-07-1.42e-06	0.891-2.51	2.93-9.55

Table includes numbering of QTLs (based on position), the chromosome, positions of SNPs associated with the QTL, MAF of these SNPs based on all accessions, which traits were associated, in which trials they were associated, which methods detected the associations, P-value range from the parametric methods, P-value range from the permutation method, range of slopes for all methods, and the range of variance explained by single SNPs of the traits based on the aforementioned slopes.

Comparing our results to historically mapped QTLs (Mouritzen et al., 2025), we found several overlaps. Firstly, a study by Ohm et al., 2024 identified a SNP on chr1_partA at position 17’608’434, overlapping with QTL 1. In their study, the SNP was associated with maturation time, plant height, and seed yield, while it was associated with sterile tillers in our analysis. Although these are not the same traits, a certain degree of similarity is found between these traits in our data, as plant height was moderately negatively correlated (r=-0.49) with sterile tillers (**Extended data figure 2**). The association with sterile tillers and end of flowering is not completely novel, as, although it has not been observed before in GWAS, the genomic region has previously been found to be associated with these traits by Skovbjerg et al., 2023 in a 7-panel MAGIC panel using a different SNP set. In this study, the region originally stood out for being strongly associated with differentiation of faba bean subpopulations. Other QTLs reporting interesting overlaps include QTL 8, where Bornhofen et al., 2024 identified a genomic region associated with earliness of flowering on chromosome 3 at position 1’187’038’964, around 2.7 Mb from QTL 8, also associated with earliness and end flowering in this study. All 13 QTLs had some degree of overlap with previously mapped QTLs. However, these overlaps should be interpreted with care since mapped traits differ, and some previously reported QTLs span hundreds of Mb. A full overview of overlapping QTLs can be found in **Supplementary file 1** Overlapping QTLs.

Although the grouping procedure allowed for QTLs up to 1 Mb in length, most identified QTLs (apart from QTL 1) spanned less than 100 bp, which might demonstrate the high resolution of our mapping approach, although this is of course limited by the clustered nature of polymorphisms derived from SPET genotyping. To more accurately estimate the genomic intervals associated with each QTL, we examined local LD structure. Specifically, by using a much denser SNP set based on resequencing data (Bornhofen et al., 2024), we identified all SNPs in this new, denser set within an even wider window of 5 Mb upstream and downstream around each QTL that were in medium-strong LD (*R*^2^ ≥ 0.5) with the associated SNPs.

Overall, top SNPs from each QTL were present in the resequencing dataset in 8 out of 13 QTLs, with 10506 SNPs being determined to be in LD with these within the SNP set (**Supplementary file 1** SNP consequence). From these SNPs, we were able to define the approximate regions these QTL were associated with (**Supplementary file 1** Candidate Span, **Extended data figures 3–10**). The regions the QTL spanned were much wider in this approach, with spans from ~390 kb for QTL 4 to ~9.7 Mb for QTL 6. However, this changes drastically if a higher threshold for LD is used, such as *R*^2^ ≥ 0.8 as was used for initial grouping of the GWAS results, bringing the number of SNPs down to 1636, with most of these being associated with QTL 4 (221) and QTL 1 (1396), 19 SNPs divided among the other 6 QTLs, bringing the candidate regions to a much more manageable size, and narrowing down the number of candidate genes to 15 (**Table 3**). The consequence of each SNP in LD with the associated SNP was also annotated based on which genomic feature it occupies (**Supplementary file 1** SNP consequence). Missense or splice region variants were annotated for SNPs with LD ≥ 0.8 for QTLs 1, 5, 6 and 10, highlighting possible causal variants (**Table 3**).

**Table 3:** Candidate genes and possible causal variants.

Transcript	Chromosome	Start	End	Annotation	QTL	Trait	Variant Type	Nucleotide Change	Amino Acid Change	LD	Distance to QTL
Vfaba.Hedin2.R2.1g000058.1	chr1_partA	17402419	17402856	Ribosome Recycling Factor	1	Sterile Tillers End Flowering					
Vfaba.Hedin2.R2.1g000059.1	chr1_partA	17606672	17609877	Protein Nrt1/ Ptr Family 6.1	1	Sterile Tillers End Flowering					
Vfaba.Hedin2.R2.1g000059.2	chr1_partA	17606880	17607431	Protein Nrt1/ Ptr Family 6.1	1	Sterile Tillers End Flowering					
Vfaba.Hedin2.R2.1g000060.1	chr1_partA	17932951	17937256	Straw Transaldolase	1	Sterile Tillers End Flowering					
Vfaba.Hedin2.R2.1g000061.1	chr1_partA	18365163	18368857	Peptidyl-Prolyl Cis-Trans Isomerase Fkbp19, Chloroplastic	1	Sterile Tillers End Flowering					
Vfaba.Hedin2.R2.1g000062.1	chr1_partA	19266613	19270314	Protein Flowering Locus T 1	1	Sterile Tillers End Flowering	Missense	c.232T>C	p.Ser78Pro	0.851	900815
Vfaba.Hedin2.R2.1g008790.1	chr1_partB	951105969	951106760	Glutathione S-Transferase T3	4	End Flowering					
Vfaba.Hedin2.R2.1g008791.1	chr1_partB	951307208	951308178	Spx Domain-Containing Protein 2	4	End Flowering					
Vfaba.Hedin2.R2.2g014531.2	chr2	979319246	979322732	Oxysterol-Binding Protein-Related Protein 1c	5	Seed Circularity type 2	Missense	c.348C>A	p.Asn116Lys	0.906	185
Vfaba.Hedin2.R2.3g018095.2	chr3	187880407	187882997	L10 Interacting Myb Domain Containing Protein	6	Lodging	Missense	c.1153A>G	p.Arg385Gly	1	0
Vfaba.Hedin2.R2.3g018156.1	chr3	211433990	211439071	Serine Carboxypeptidase-Like 49	7	End Flowering					
Vfaba.Hedin2.R2.3g022356.1	chr3	1593290792	1593300797	Rrm Domain Containing Protein	10	Maturation Date End Flowering	Splice region Missense	c.2696+8A>G c.1136C>T	p.Ser379Leu	0.943 0.93	3753 5406
Vfaba.Hedin2.R2.5g029398.1	chr5	357034459	357039423	Ammonium Transporter 2	11	Duration of Flowering					
Vfaba.Hedin2.R2.5g030028.1	chr5	535097021	535102328	Phenylalanine--Trna Ligase, Chloroplastic/Mitochondrial	12	Plant Height					

Transcripts in which QTLs have SNPs associated with QTL top SNPs in the resequencing dataset. For each transcript is annotated the chromosome, the start and end of the transcript, the functional annotation, which QTL it is associated with, which traits the QTL was originally associated with, and if any associated SNPs are missense, stop or splice region mutants, that is annotated along with the nucleotide and amino acid change, where possible, along with those SNPs LD with lead SNPs, and their distance to lead SNPs. Be aware that for the transcript with QTL 6 the candidate SNP is the actual top SNP, so LD is 1 and distance is 0.

This revealed interesting candidates for several QTLs. For QTL 1 we could annotate both *NRT1* and *FLOWERING LOCUS T* as being associated with the QTL. *NRT1* has previously reported roles in nitrate, peptide and plant hormone transport impacting plant growth, nitrogen use efficiency, and seed yield (Wang and Tsay, 2011; Fang et al., 2013; Chiba et al., 2015), while *FLOWERING LOCUS T* is well characterized as a mobile signaling for flowering (Wickland and Hanzawa, 2015). Furthermore, *FLOWERING LOCUS T* is annotated to have a missense mutation (serine to proline) in high LD with our top SNP. In QTL 10, a gene from a family with established roles in flowering time and developmental maturity was identified, *Vfaba.Hedin2.R2.3g022356*, which encodes a protein containing an RNA Recognition Motif (RRM). RRM-containing proteins have been implicated in the regulation of flowering, with several members shown to repress key floral regulators such as *FLOWERING LOCUS M* and *FLOWERING LOCUS C* (Bäurle et al., 2007; Zhao et al., 2022). This gene was annotated to have a missense mutation (serine to leucine) and a mutation in the splice region of the intron, both in high LD with our top SNP. Overall, several of the putative candidate genes identified exhibit strong biological plausibility and warrant further investigation through functional validation studies.

### 3.4 Evaluation of permutation approach

**Figure 2:**
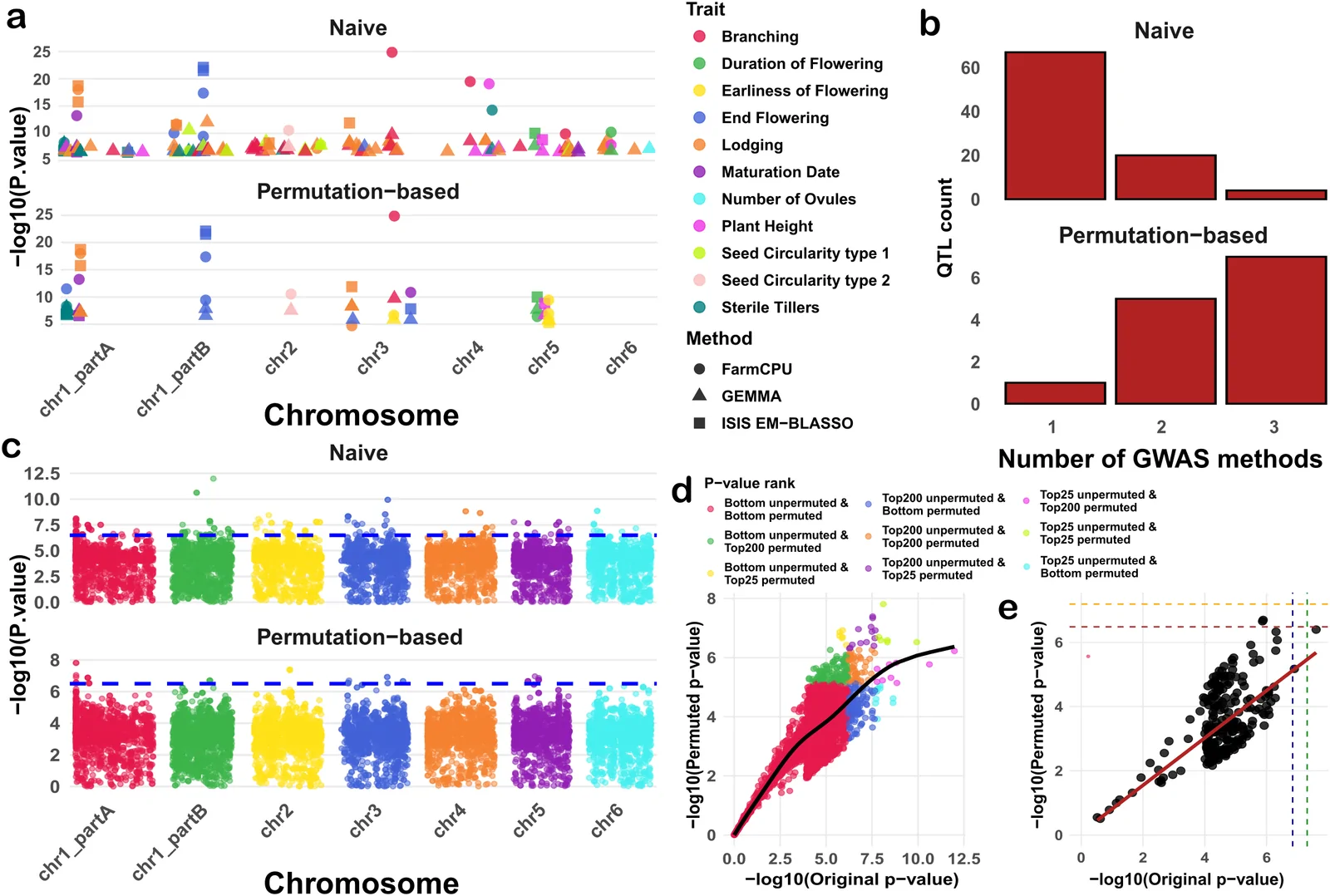
GWAS and permutation *post-hoc* adjustment results. **(A)** Manhattan plot comparing non-permuted *post-hoc* filtered results (naïve) with permutation-based results for all traits. Only significant QTLs for each, respective procedure are shown. The p-values shown are the original, parametric p-values. **(B)** Comparison of naïve and permutation-based results on the number of GWAS methods that were significant for each QTL. **(C)** Manhattan plot of naïve and permutation-based results. Shown are only parametric p-values from GEMMA (naïve) or the permutation-derived p-values. Results are from all traits, with the dashed, blue line representing the mean p-value significance threshold across traits. **(D)** GEMMA parametric p-values (unpermuted) plotted against permutation-derived p-values for all traits. Only p-values originally below 10^−4^ in any method are shown, and p-values are -log10 transformed. P-values are divided into groups, based on whether they are in the top 25, top 200, or the bottom rest of each of the two p-values associated with each point, resulting in 9 combinations. To the points is fitted a smooth spline constrained to be monotonously increasing. **(E)** GEMMA parametric p-values (unpermuted) plotted against permutation-derived p-values for End of Flowering in Nordic Seed 2018. Only p-values originally below 10−4 in any of the three GWAS methods (FarmCPU, GEMMA, ISIS EM-BLASSO) are shown, and p-values are -log10 transformed. To the points is fitted a smooth spline constrained to be monotonously increasing. Four p-value thresholds are shown as dashed lines. The yellow and red horizontal lines are the 0.01 and 0.05 thresholds estimated using simpleM, while the green and blue vertical lines are the 0.01 and 0.05 thresholds estimated using PermGWAS2.

To evaluate the added value of permutation-corrected *p*-values, we developed a comparison GWAS set filtered using a “naïve” approach, where *p*-values are not recalculated with permutation, but otherwise treated the same (**Supplementary file 1** Naïve GWAS results). This set had a total of 119 associations, corresponding to 105 unique SNPs within 91 QTLs, a distinctly larger number than what was present in the set filtered using the permutation-based approach (**Figure 2A**). The immediate quality of the permutation-based hits seemed higher, with the number of GWAS methods associated with each QTL much higher than in the naïve set (**Figure 2B**). A similar, but less strong signature was also seen for the number of traits, trials and SNPs per QTL (**Extended data figures 11–13**). Comparing *p*-values obtained from GEMMA with those derived from our permutation-based procedure shows a general upward shift in *p*-values, but with significant heterogeneity in this effect across the associations (**Figure 2C**). This non-uniform rescaling produces a Manhattan plot with a greater separation between the general population of SNPs and the significance threshold and a more distinct set of prominent peaks.

In the following section, classifications of true and false positives and negatives are defined relative to the GEMMA framework rather than to the underlying biological truth, as the true causal status of individual SNP-trait associations is unknown and cannot be used to directly classify results as true or false discoveries. Instead, we treat permutation-derived *p*-values as an empirical reference and evaluate the parametric *p*-values produced by GEMMA’s linear mixed model against this reference. In this, discrepancies are interpreted as caused by violations of the statistical assumptions underlying the model not affecting the empirical distribution generated by permutation, within Monte Carlo error. Consequently, classifications such as false positives and false negatives refer to disagreement with this permutation-based reference, rather than to the unknown biological truth.

When plotting permutation-derived *p*-values against parametric GEMMA *p*-values, there is an overall correlation, but with significant dispersion around this trend (**Figure 2D**). Such variability implies that relying solely on the original parametric *p*-values can inflate both type I and type II error rates. To illustrate this, we stratified associations into groups based on whether they rank within the top 25, top 200, or the remaining associations according to either the original or permutation-adjusted *p*-values, resulting in nine distinct categories in the two-dimensional plot (**Figure 2D**). Selecting the top 200 associations using the original *p*-values would incorrectly include the blue and cyan groups while excluding the green and yellow groups, thereby increasing both type I and type II errors, respectively. A similar pattern emerges when selecting the top 25 associations, where the pink and cyan points are erroneously included and the purple and yellow points are omitted.

This pattern is consistent with previous findings in the literature, which have demonstrated that GWAS methods are particularly vulnerable to violations of statistical assumptions, particularly due to the winner’s curse effect. To address this issue, John et al., 2024, developed the PermGWAS2 method, utilizing a few permutations of all SNPs to set a novel, genome-wide significance threshold for each individual GWAS run. Through this approach, false positive rates can be effectively controlled, rendering the method especially robust when dealing with skewed phenotype distributions.

To evaluate this approach against ours, we calculated PermGWAS2-based *p*-value thresholds for each GWAS run (**Supplementary file 1** Permutation thresholds). Applying these thresholds yields 42 and 20 significant associations at *α* = 0.05 and *α* = 0.01, respectively. In comparison, our method produced 19 and 3 associations at the same thresholds, with 12 and 2 overlapping associations between the two approaches (**Table 4**; **Supplementary file 1** Permutation overlaps 0.01 & 0.05). Examination of individual GWAS runs indicates that the primary difference is in strictness of the methods, with minimal variation between permutation-based and parametric p-values for many traits (**Extended data figures 14-65**). Despite these similarities, substantial numerical differences persist for some GWAS runs, for example for end of flowering in the Nordic Seed 2018 trial (**Figure 2D**). Adjusting the false positive rate alone here would be insufficient to mitigate type II errors. Several strongly associated SNPs were undervalued by parametric *p*-values, while other weaker associations are overestimated for this trait. Consequently, any method that aims to reduce type I error by recalibrating parametric *p*-value thresholds inevitably risks an excessive loss of statistical power for some traits.

**Table 4:** PermGWAS2 and post-hoc permutation method comparison.

Method	Hits	Overlaps	Computation time	Memory	CPU	GPU
PermGWAS2	42	12	4.3	64 Gb	AMD EPYC 9575F	Nvidia H200
Post-hoc Permutation	19	12	914	8 Gb	AMD EPYC 9654	NA

Comparison between PermGWAS2 and *post-hoc* permutation on how many significant hits each method had, how many overlaps that were between associations, the computation time (in hours), the approximate amount of memory used, and what hardware (CPU and GPU) the methods were performed on. The *post-hoc* permutation approach did not use any GPU for its algorithm.

These results are consistent with previous observations (Asif et al., 2021), where reduced concordance between parametric and permutation-based p-values at lower MAFs and smaller sample sizes were reported. In effect, because the permGWAS2 threshold is estimated across all SNPs, it may be miscalibrated for traits that exhibit a less perfect correlation between parametric and permutation-based p-values, becoming overly permissive at low MAF and overly conservative at high MAF. Our approach avoids this bias, though it is computationally more demanding, especially at larger sample sizes. Our approach took a total of 914 hours, compared to only 4.3 for PermGWAS2 (**Table 4**). However, this difference should be interpreted in light of the GPU acceleration and higher memory footprint of PermGWAS2, which contribute to overall computational resource costs (**Table 4**). Furthermore, while PermGWAS2 estimates variance components once, GEMMA, which we used for our permutation approach, re-estimates variance components for each SNP over several iterations.

Despite the higher computational demand, runtime remains practical and feasible for small to moderate datasets, such as the NORFAB panel. The increase in statistical power may justify these costs, particularly in smaller samples where detection is otherwise limited. Moreover, these computation costs should be considered relative to upstream and downstream expenses, such as field trials and functional validation.

## 4 Discussion

A key result of this study is that *post-hoc* recalculation of *p*-values for top GWAS signals through a permutation-based approach substantially reduced spurious associations caused by model violations. We demonstrated that this *post hoc* refinement increased the proportion of QTL detected by multiple methods, but also across more trials on average, suggesting that loci discovered here might be more likely to have an effect across diverse environments. This potentially make them more relevant across more breeding contexts, either as stable markers or markers conferring a GxE effect. In practical terms, permutation-filtering enhances the credibility of marker-trait associations which might also improve their potential value in breeding pipelines. We show our method is distinct from the PermGWAS2 method, with distinct results, and that our method has a potential improvement in type II errors and even in type I errors for traits with a strong decoupling of parametric and permutation-derived *p*-values. However, we acknowledge that our method is substantially slower, especially for larger sample sizes, in which case PermGWAS2 is a very fast and powerful method compared to regular GWAS.

Through our permutation-based approach, we identified a set of robust and biologically meaningful QTLs associated with diverse agronomic traits in faba bean. The consistency of these associations across multiple traits, environments, and analytical methods supports their validity and biological relevance. Moreover, the overlap of our findings with QTLs reported in previous faba bean GWAS strengthens their general validity. Several associations involve SNPs located within or near genes with plausible functional hypotheses or supported by previous literature, such as the *NTR1*-family genes. This example is particularly interesting for functional validation and follow-up, as the transport function and expression profile of this specific *NTR1*-family member remains unclear. In contrast, associations involving genes with well-characterized function, such as the RRM domain containing gene or *FLOWERING LOCUS T*, may offer less novelty for functional exploration beyond validation but remain potentially valuable as reliable targets for breeding and their strong candidate status further support the robustness of our approach.

Nevertheless, several limitations of our study warrant consideration. First, sample sizes were modest for some traits, which constrains statistical power in GWAS given the need to correct for multiple testing. A lower amount of samples might also be more subject to false positives, given the higher effect of what has been termed the winner’s curse or the Beavis effect (Beavis and Wilkinson, 1994; Paterson and Press, 1998; Xu, 2003; Palmer and Pe’er, 2017). This likely contributed to the small number of QTLs retained after permutation filtering, which was lower than the number of independent GWAS scans performed. This also implies that the method might be more likely to remove spurious associations in smaller samples. Although this means this effect would be attenuated in larger samples, the associated computational demands may also render the method impractical before this point. This might also be attributed to other effects, such as more complex trait architecture, such as many low-effect variants, non-additive effects, or even GxE effects. Traits more strongly influenced by these components may exhibit reduced GWAS quality, given the violation of the assumption of independence. An additional consideration is that the phenotypic dataset is unbalanced. Although statistical modeling has been applied to mitigate this issue, the imbalance may still influence the estimated phenotypic values used in the GWAS. As such, there is a risk that detected associations reflect some sort of correlation with the underlying data imbalance rather than a real genetic signal, or that genuine relationships are obscured compared to what might be observed under a fully balanced design.

Lastly, our procedure also showed it was not method-agnostic, with all QTLs showing association with GEMMA in the parametric GWAS. This observation is important because it suggest that the *post-hoc* permutation remains inherently dependent on some aspect of the GEMMA framework. In other words, the permutation procedure does not operate entirely independently but instead inherits certain constraints from the underlying model. While, in principle, similar permutation strategies could be adopted for other GWAS approaches, the ease of implementation is likely to vary considerably. For example, in iterative multi-locus methods such as FarmCPU, permutation would need to be applied across multiple iterative steps for all variants tested, substantially increasing computational demands.

Altogether, these findings highlight both the progress and challenges in refining GWAS pipelines for polygenic and environmentally sensitive traits in faba bean. We showed that a *post-hoc* permutation recalculation has the potential to improve robustness of results, particularly in smaller sample sizes and when traits exhibit behaviors where there is less concordance between parametric and permutation-based *p*-values, at the cost of additional computational time. However, continued methodological innovation is needed to better model trait architecture and environmental complexity to improve discovery and predictive power, particularly in more complex traits. Future developments in permutation-based approaches should also prioritize computational efficiency, given that GWAS computational cost scales roughly linearly with the number of markers and cubically with the number of samples. Advancing toward scalable, high-performance frameworks will be essential to ensure that improved statistical rigor does not come at the expense of practical feasibility as datasets grow in size and complexity.

## 5 Conclusion

This study demonstrates that *post-hoc* permutation testing of top hits in GWAS can improve robustness by reducing spurious associations arising from model violations. By re-ranking top signals rather than merely redefining significance thresholds, the approach increases concordance across methods and environments, suggesting that retained loci are more likely to represent biologically meaningful and potentially breeding-relevant effects. The identification of QTLs that overlap with previous studies and are located near genes with either strong literature support or a plausible functional link to the measured traits strengthens confidence in our findings and points to promising targets for both functional characterizations and breeding applications. However, these gains in statistical rigor come with increased computational demands and have so far only been demonstrated within a GEMMA-based GWAS framework, which may limit practical scalability and generalizability to very large datasets and more computationally costly GWAS frameworks. While these constraints may be less critical in smaller datasets, where model violations are more pronounced, they nevertheless underscore the need for continued methodological innovation to improve computational efficiency and extend permutation-based refinement to a broader range of GWAS models.

## Data Availability

The datasets presented in this study can be found in online repositories. The names of the repository/repositories and accession number(s) can be found in the article/**Supplementary Material**.
