# Supplementary material for: Post-hoc permutation GWAS refinement reveals robust marker-trait associations in faba bean
Source: Front Plant Sci. 2026 Aug 10;17:1895808. doi: 10.3389/fpls.2026.1895808 (PMC13500635; doi:10.3389/fpls.2026.1895808)
Supplement: Supplementary file 1 [file DataSheet1.pdf]

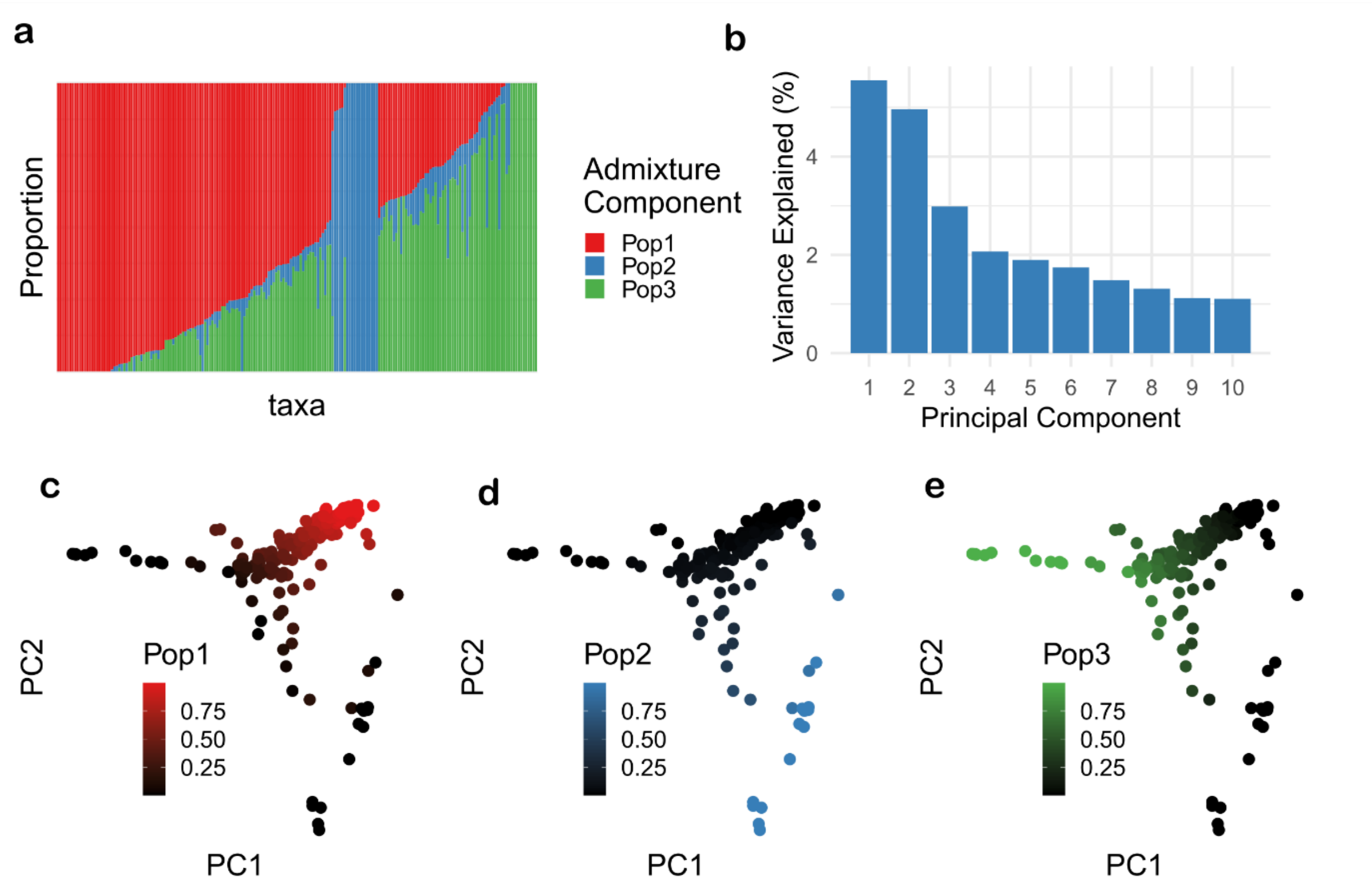

Extended data figure 1: Population structure. (a) Admixture proportions of the three estimated admixture components of all lines. Each column in the plot corresponds to a single accession. (b) Variance explained by the 10 first principal components of the genotype matrix. (c-e) All accessions placed on the 2-dimensional space formed by the two first principal components, with each accession colored by the proportion of the first (c), second (d) or third (e) admixture component.

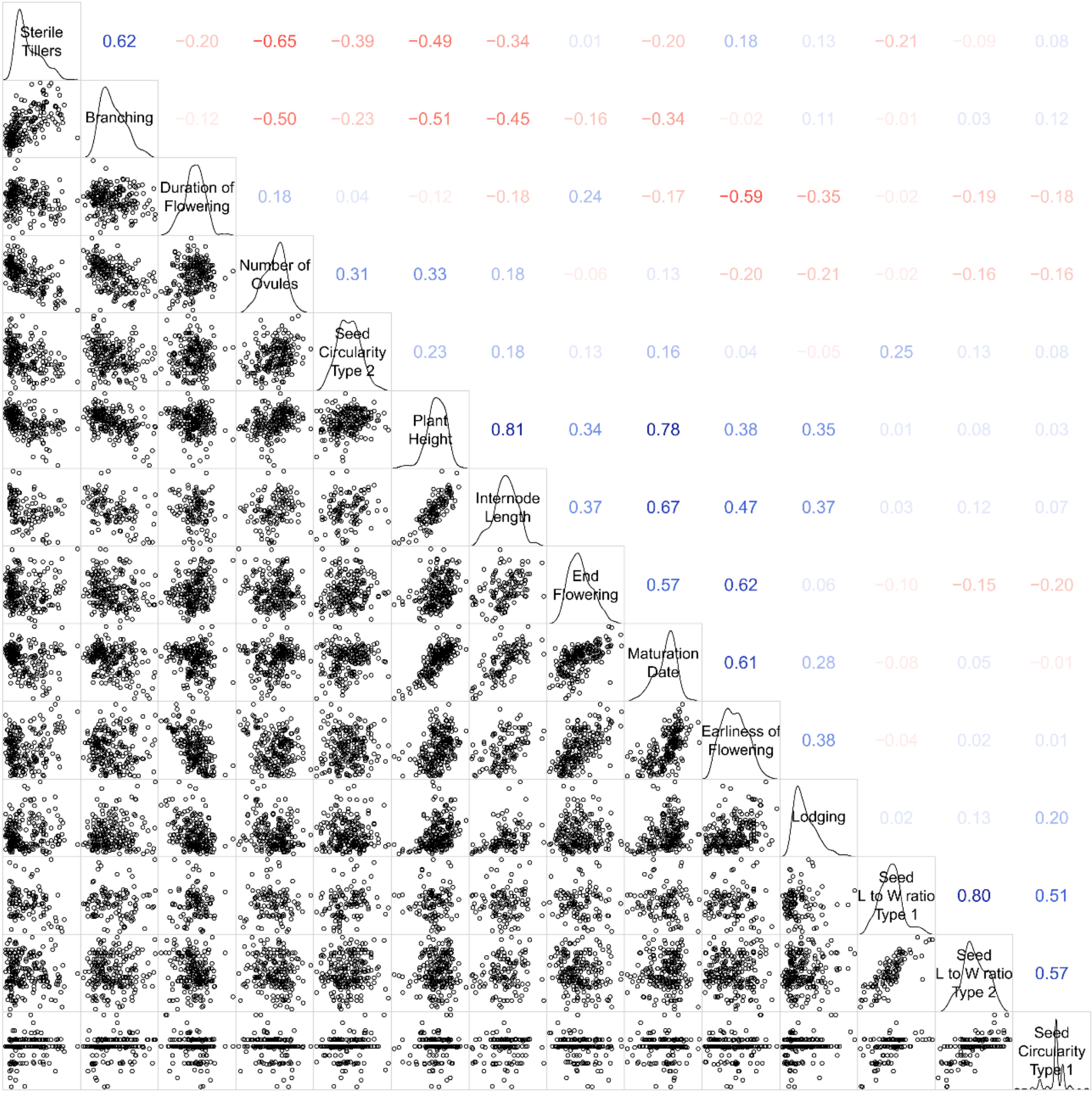

Extended data figure 2: Correlogram of BLUEs for all traits. On the diagonal all trait distributions are shown as a smoothed histogram. On the upper triangular part is shown the correlations between all combinations of traits, colored by blue or red for positive or negative correlations, respectively, the color being stronger the stronger the positive or negative correlation is. On the lower diagonal is shown the actual dual trait distribution, with the respective trait whose column and row we are in corresponding to the x and y dimensions of the scatterplot, respectively.

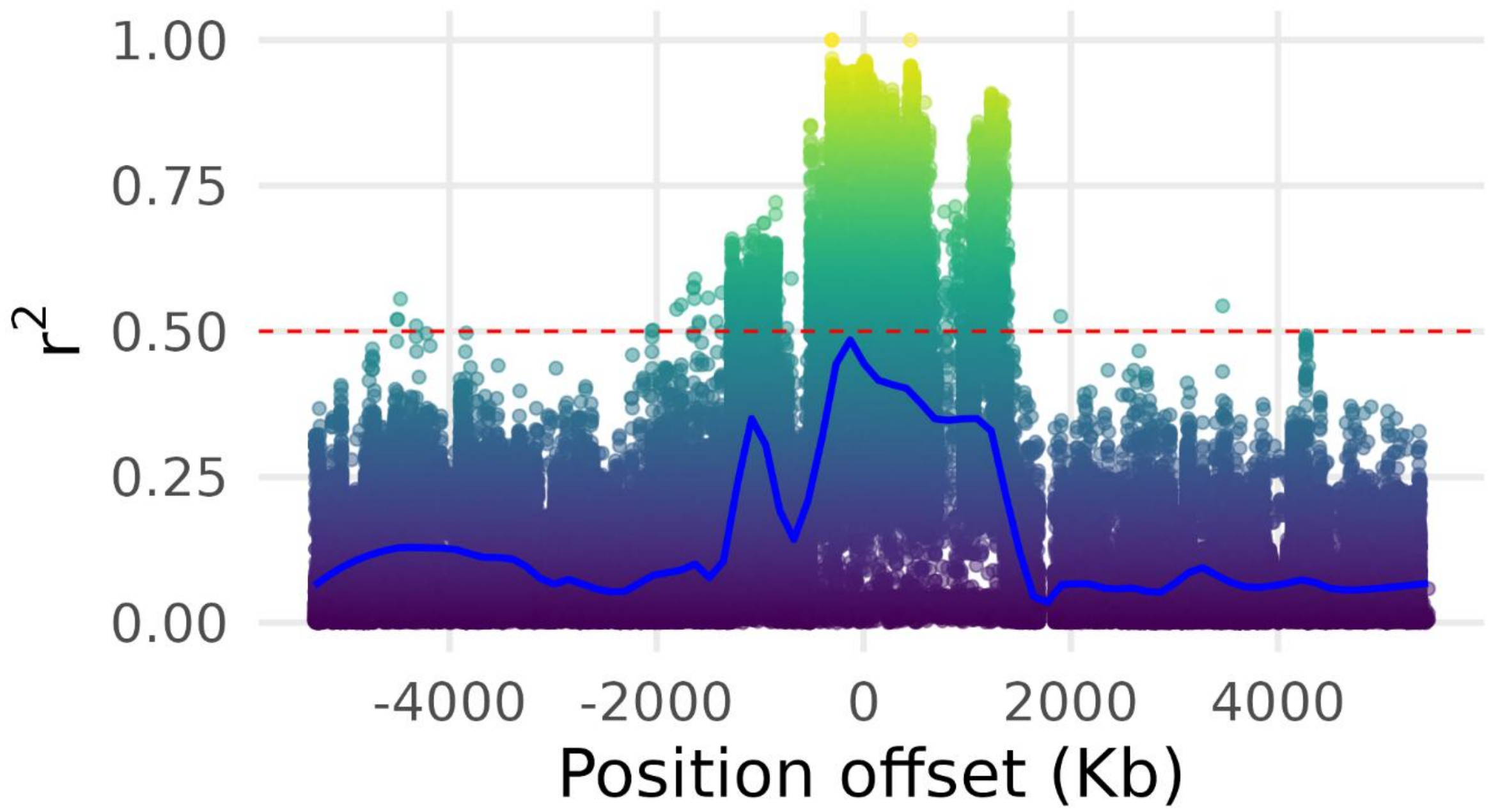

Extended data figure 3: LD pattern around QTL 1. Max R^2 values between each SNP in the resequencing dataset and the original top SNPs, with each dot corresponding to a SNP. The higher or lower the R^2 value the more yellow or purple the color, respectively. A dashed red horizontal line is drawn at an R^2 value of 0.5. In blue, a smoothed line representing a spline function fitted to the points.

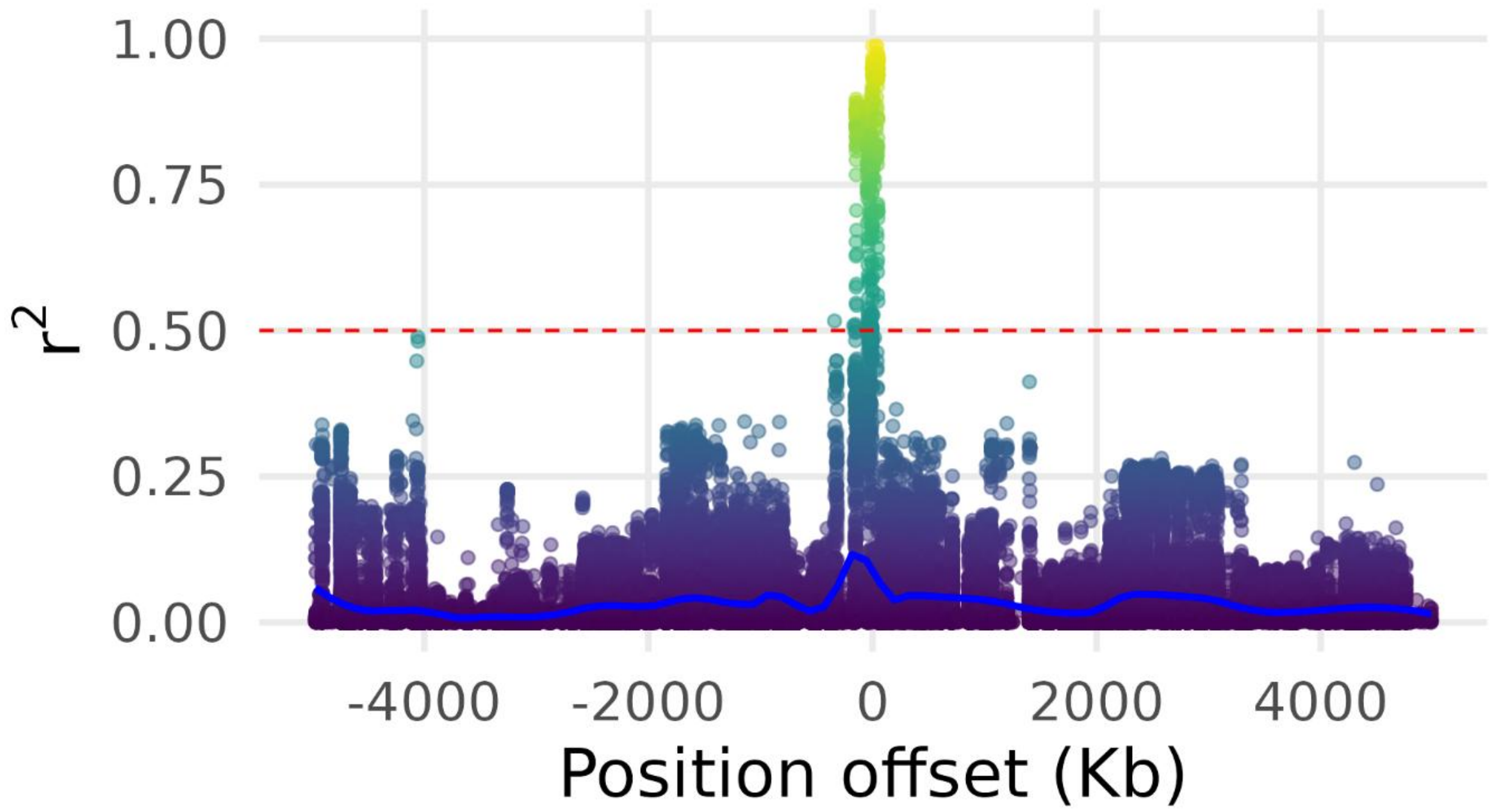

Extended data figure 4: LD pattern around QTL 4. Max R^2 values between each SNP in the resequencing dataset and the original top SNPs, with each dot corresponding to a SNP. The higher or lower the R^2 value the more yellow or purple the color, respectively. A dashed red horizontal line is drawn at an R^2 value of 0.5. In blue, a smoothed line representing a spline function fitted to the points.

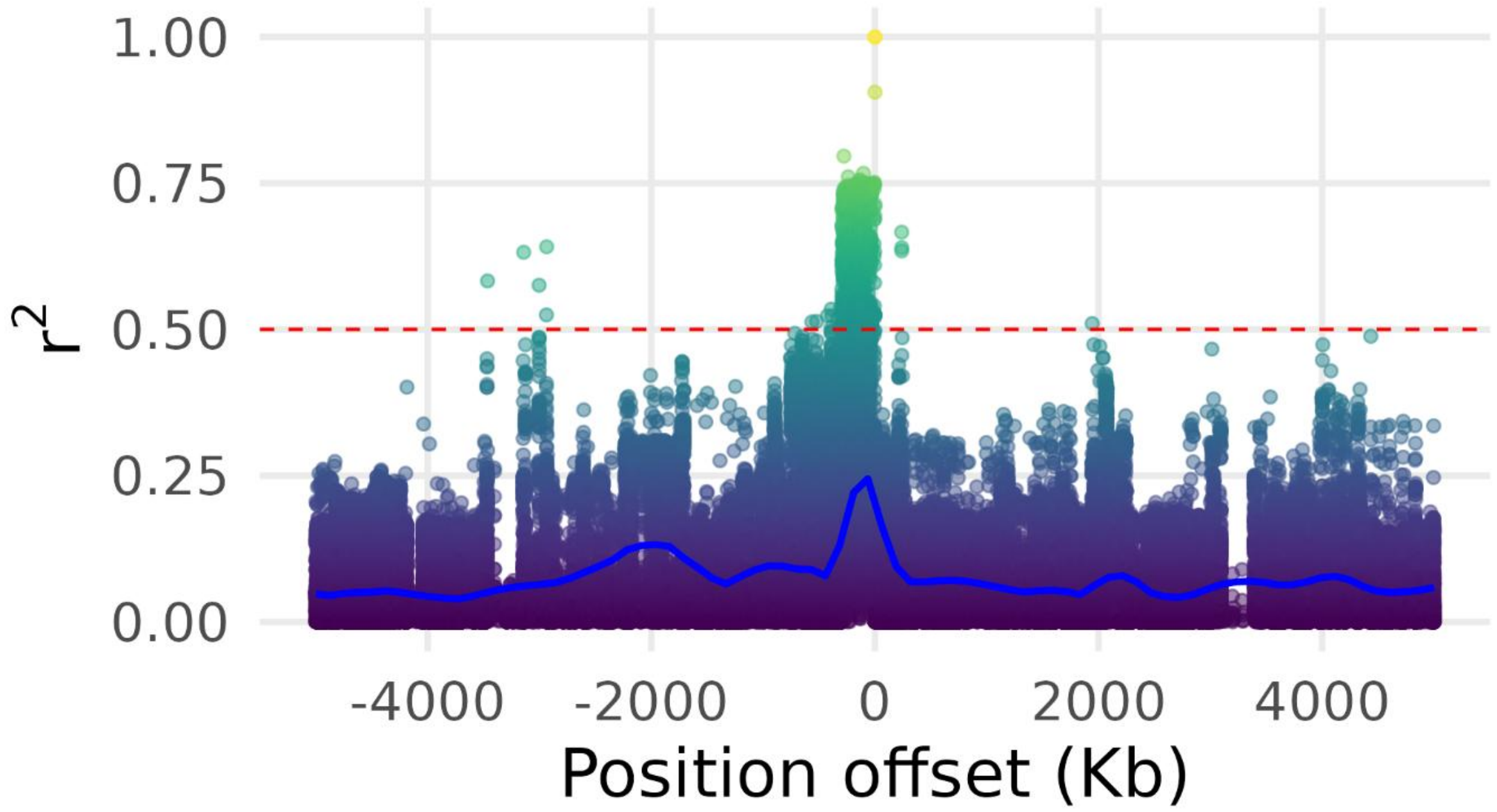

Extended data figure 5: LD pattern around QTL 5. Max R^2 values between each SNP in the resequencing dataset and the original top SNPs, with each dot corresponding to a SNP. The higher or lower the R^2 value the more yellow or purple the color, respectively. A dashed red horizontal line is drawn at an R^2 value of 0.5. In blue, a smoothed line representing a spline function fitted to the points.

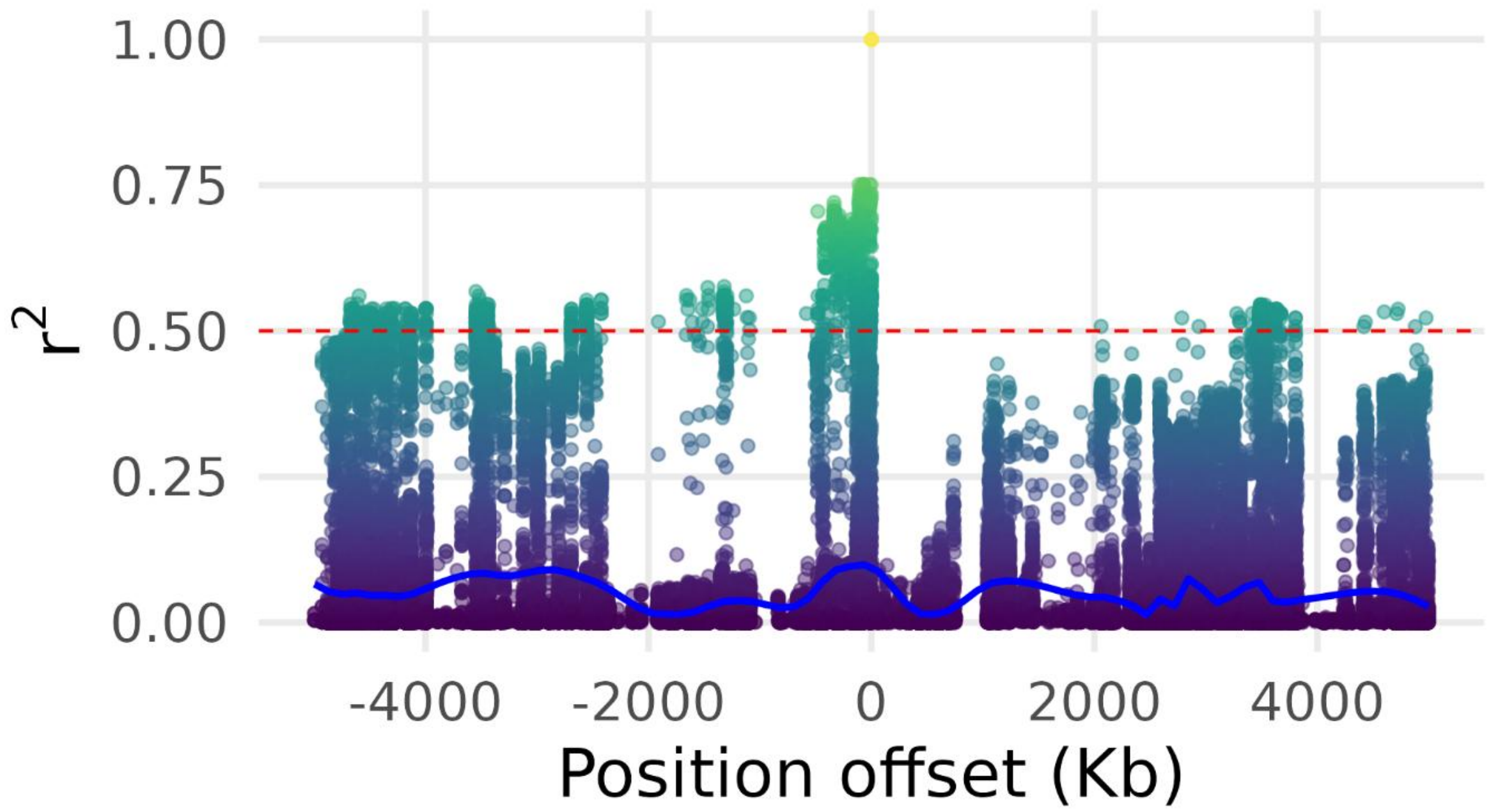

Extended data figure 6: LD pattern around QTL 6. Max R^2 values between each SNP in the resequencing dataset and the original top SNPs, with each dot corresponding to a SNP. The higher or lower the R^2 value the more yellow or purple the color, respectively. A dashed red horizontal line is drawn at an R^2 value of 0.5. In blue, a smoothed line representing a spline function fitted to the points.

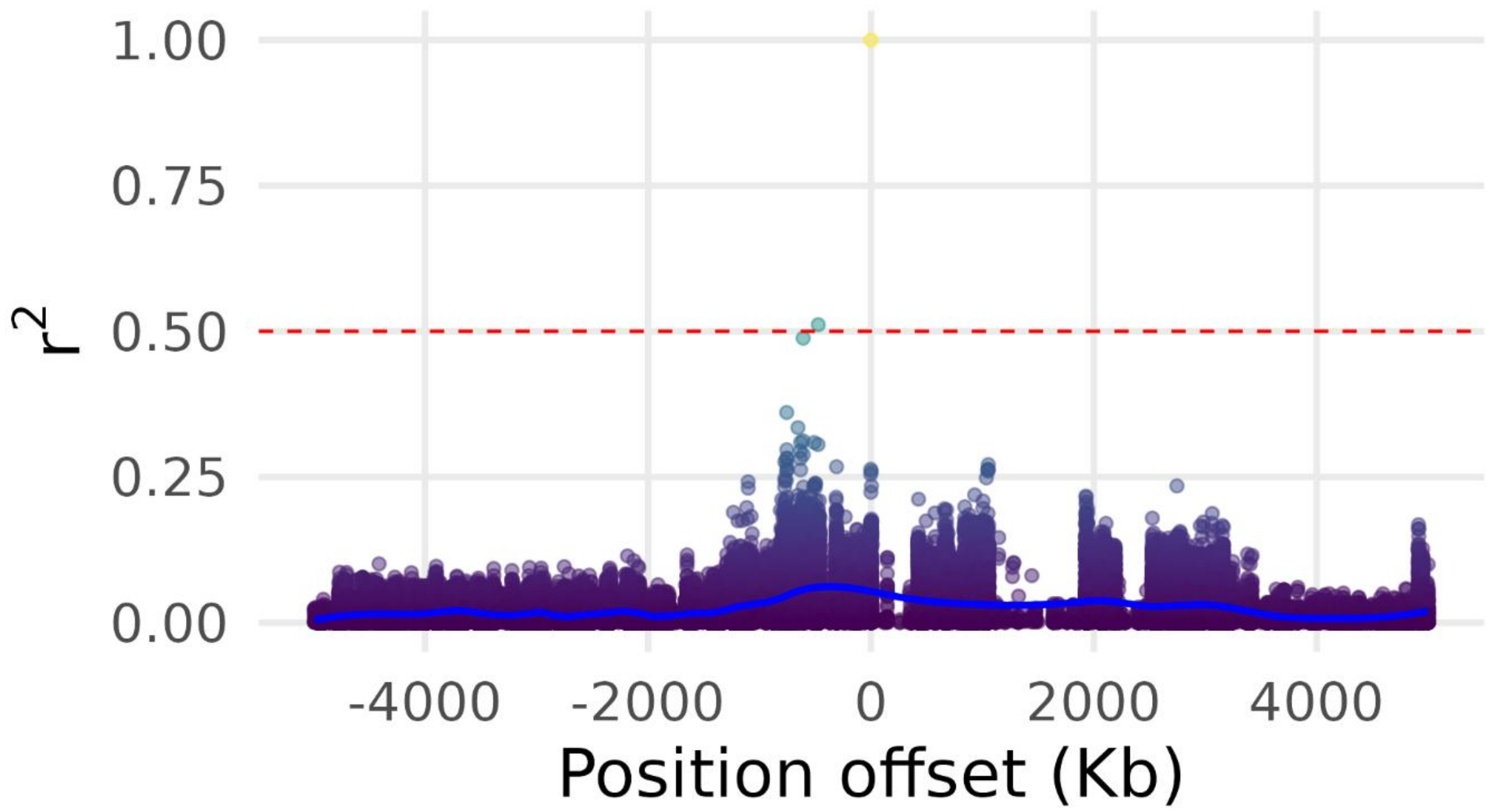

Extended data figure 7: LD pattern around QTL 7. Max R^2 values between each SNP in the resequencing dataset and the original top SNPs, with each dot corresponding to a SNP. The higher or lower the R^2 value the more yellow or purple the color, respectively. A dashed red horizontal line is drawn at an R^2 value of 0.5. In blue, a smoothed line representing a spline function fitted to the points.

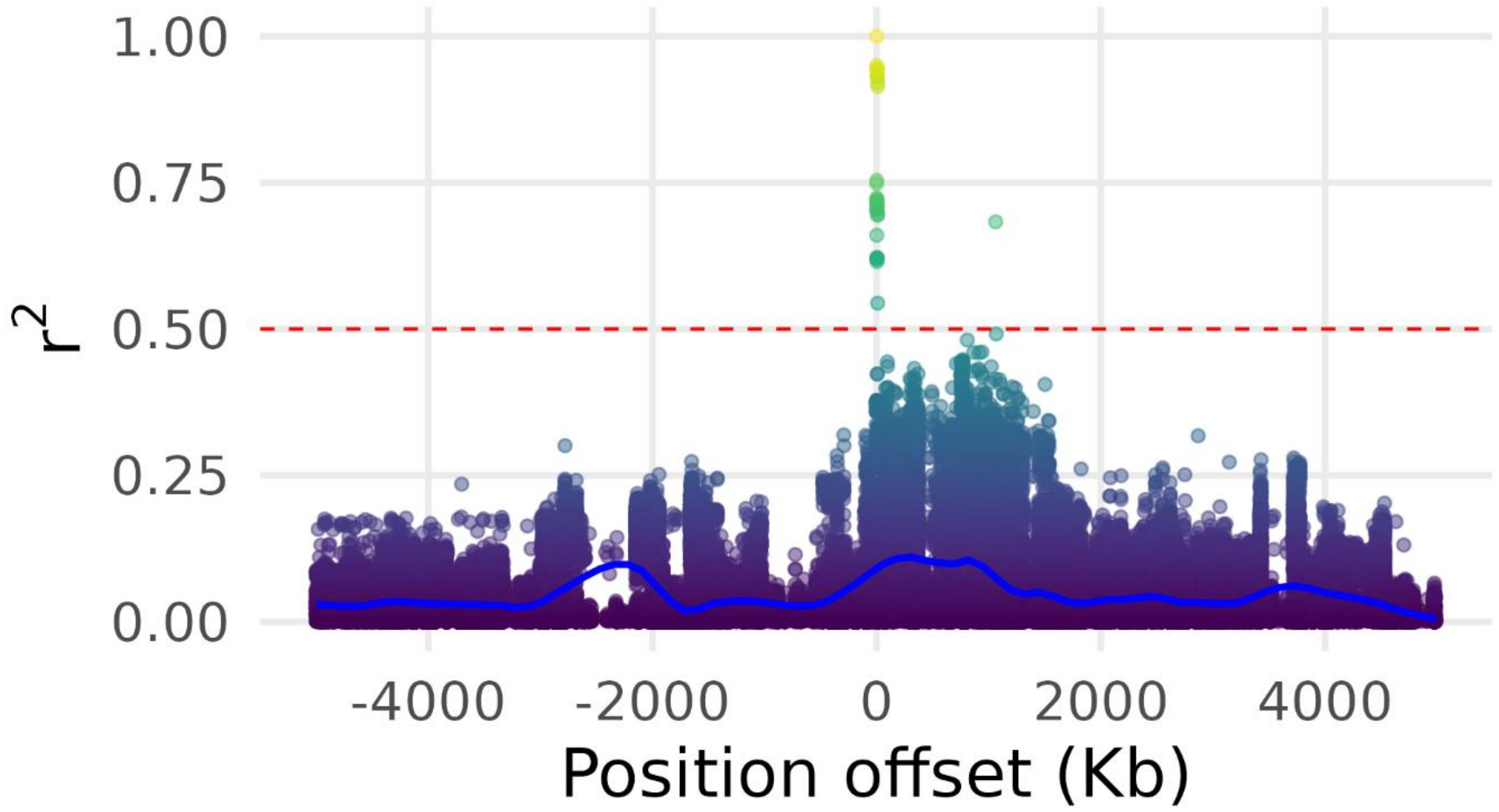

Extended data figure 8: LD pattern around QTL 10. Max R^2 values between each SNP in the resequencing dataset and the original top SNPs, with each dot corresponding to a SNP. The higher or lower the R^2 value the more yellow or purple the color, respectively. A dashed red horizontal line is drawn at an R^2 value of 0.5. In blue, a smoothed line representing a spline function fitted to the points.

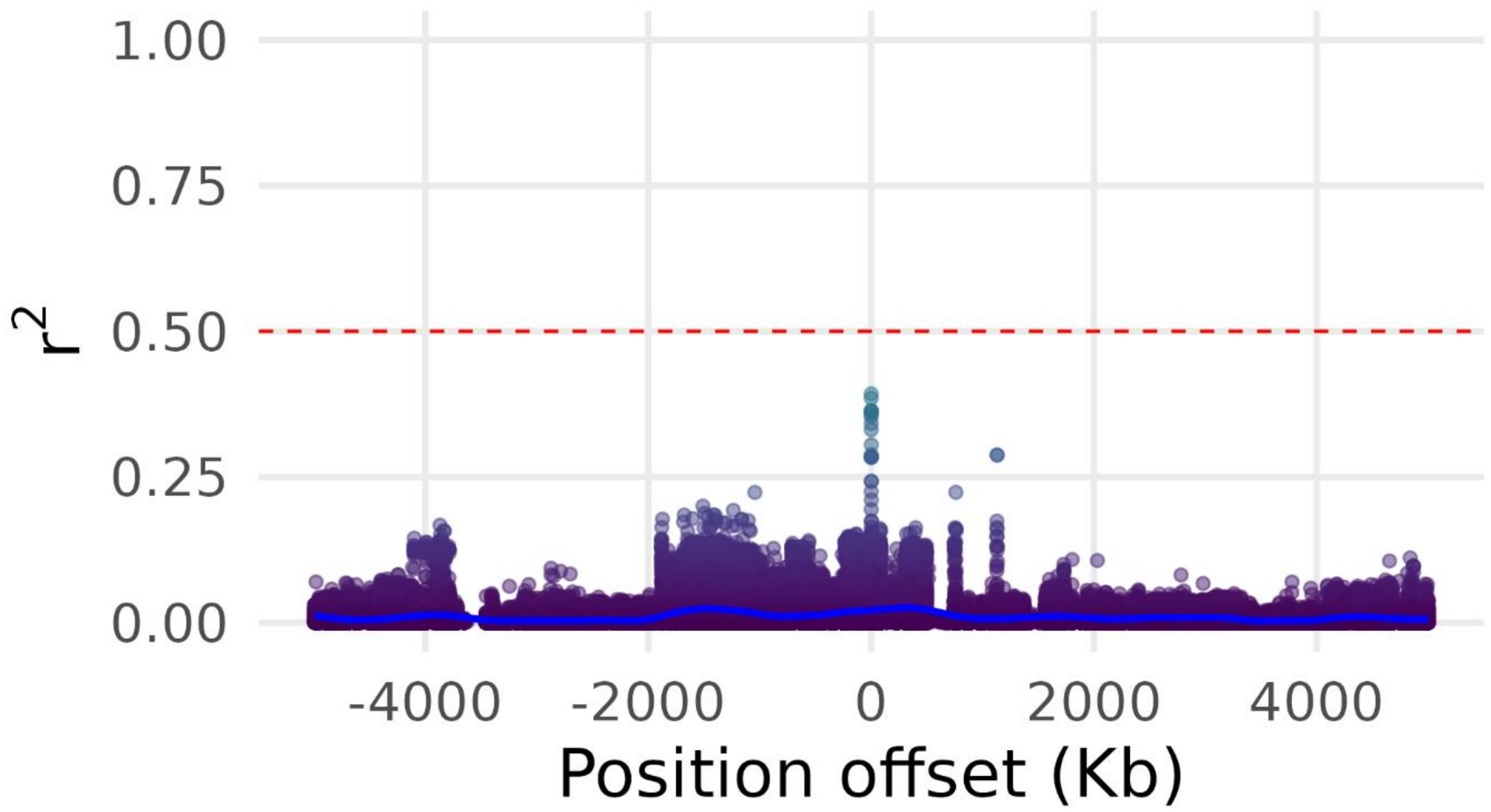

Extended data figure 9: LD pattern around QTL 11. Max R^2 values between each SNP in the resequencing dataset and the original top SNPs, with each dot corresponding to a SNP. The higher or lower the R^2 value the more yellow or purple the color, respectively. A dashed red horizontal line is drawn at an R^2 value of 0.5. In blue, a smoothed line representing a spline function fitted to the points.

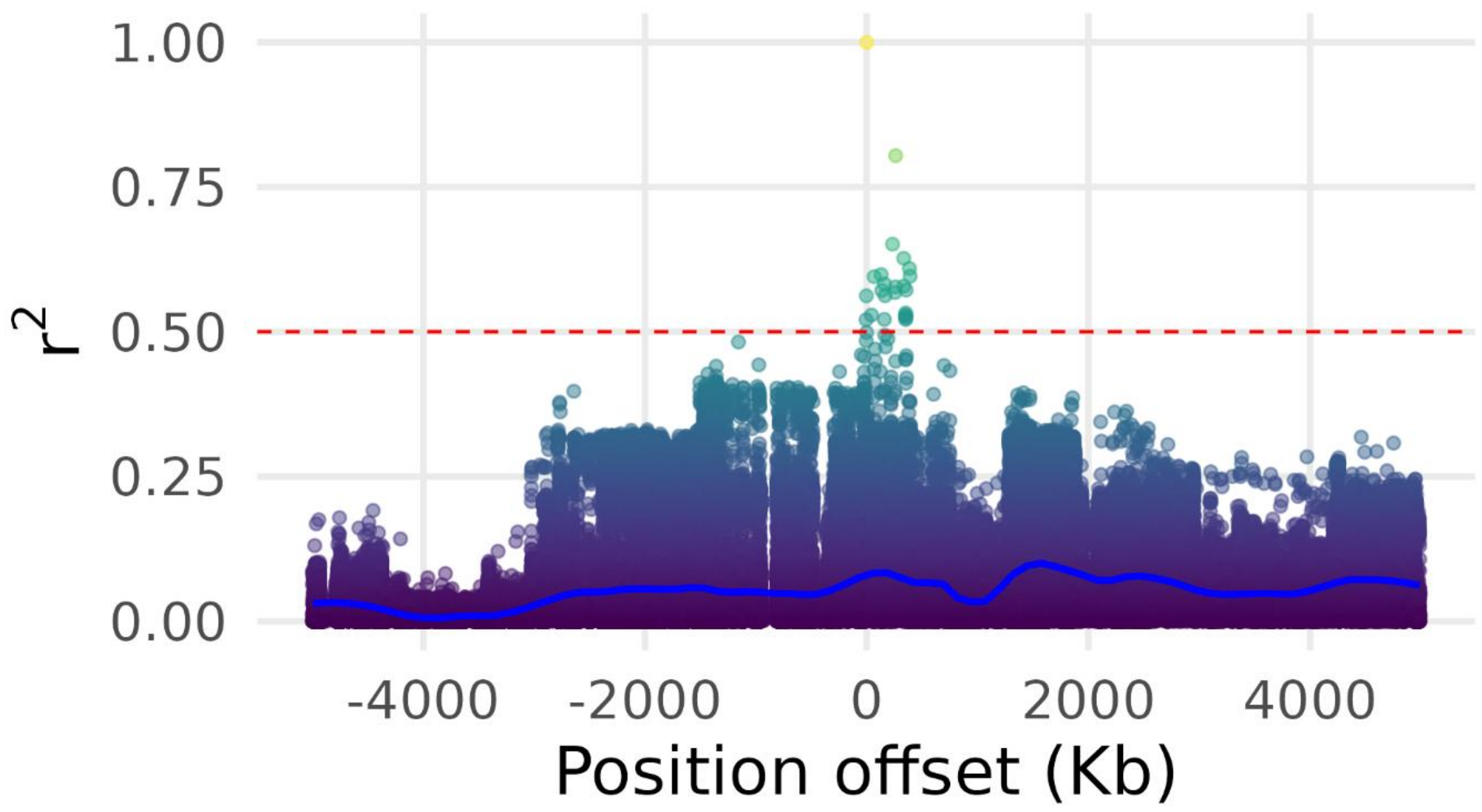

Extended data figure 10: LD pattern around QTL 12. Max R^2 values between each SNP in the resequencing dataset and the original top SNPs, with each dot corresponding to a SNP. The higher or lower the R^2 value the more yellow or purple the color, respectively. A dashed red horizontal line is drawn at an R^2 value of 0.5. In blue, a smoothed line representing a spline function fitted to the points.

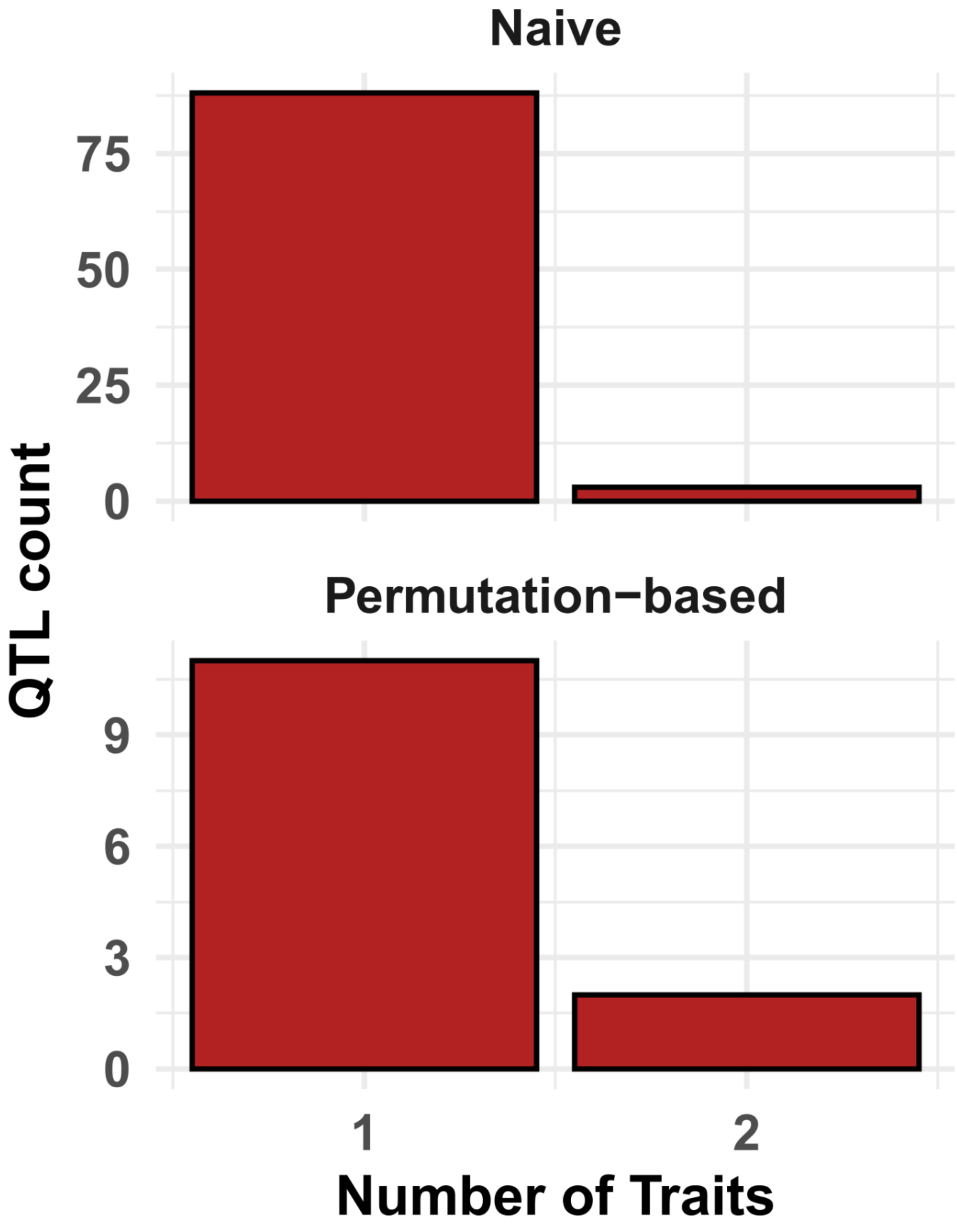

Extended data figure 11: Number of traits per QTL across different methods. Comparison of naïve and permutation-based results on the number of traits that were significant for each QTL.

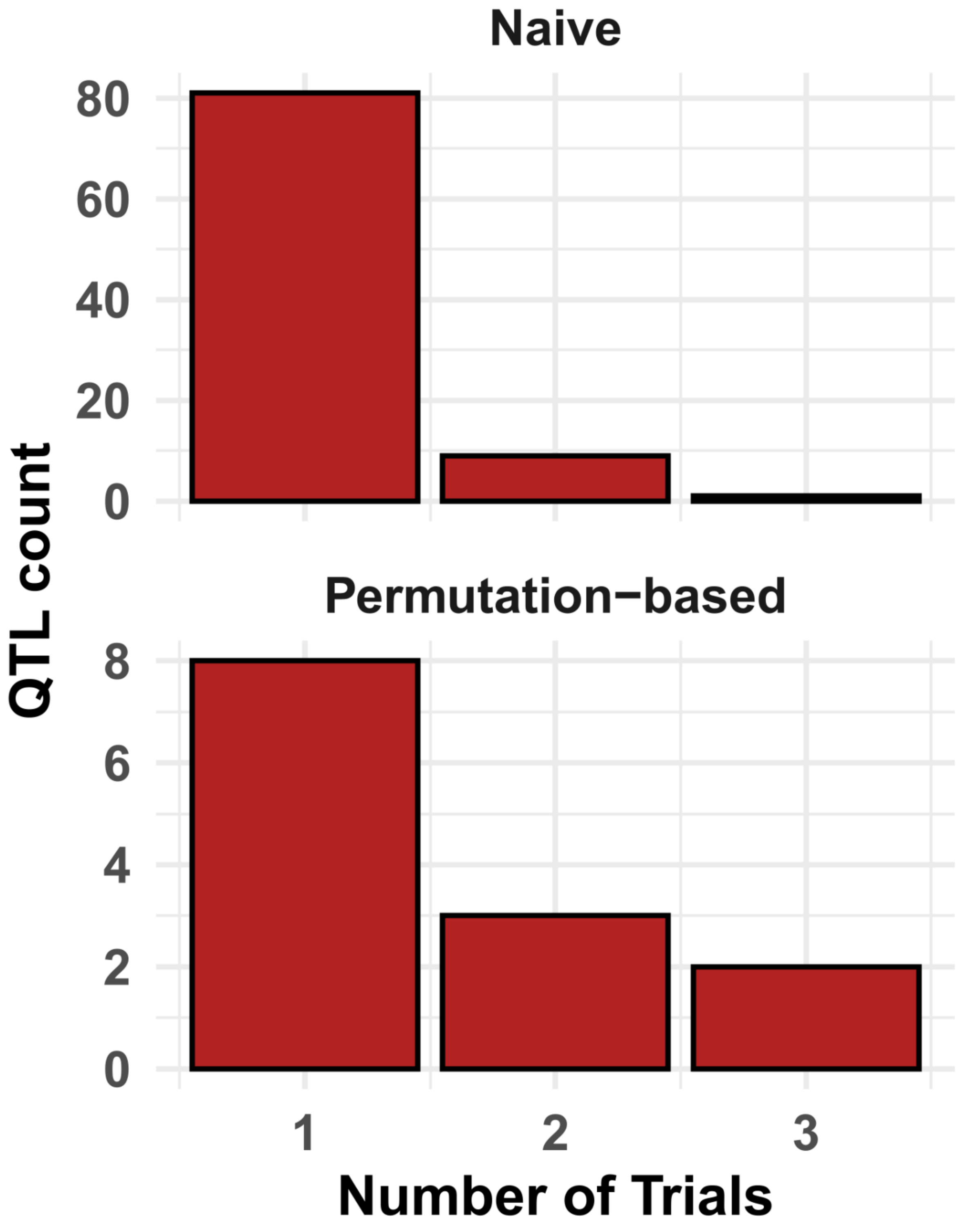

Extended data figure 12: Number of trials per QTL across different methods. Comparison of naïve and permutation-based results on the number of trials that were significant for each QTL.

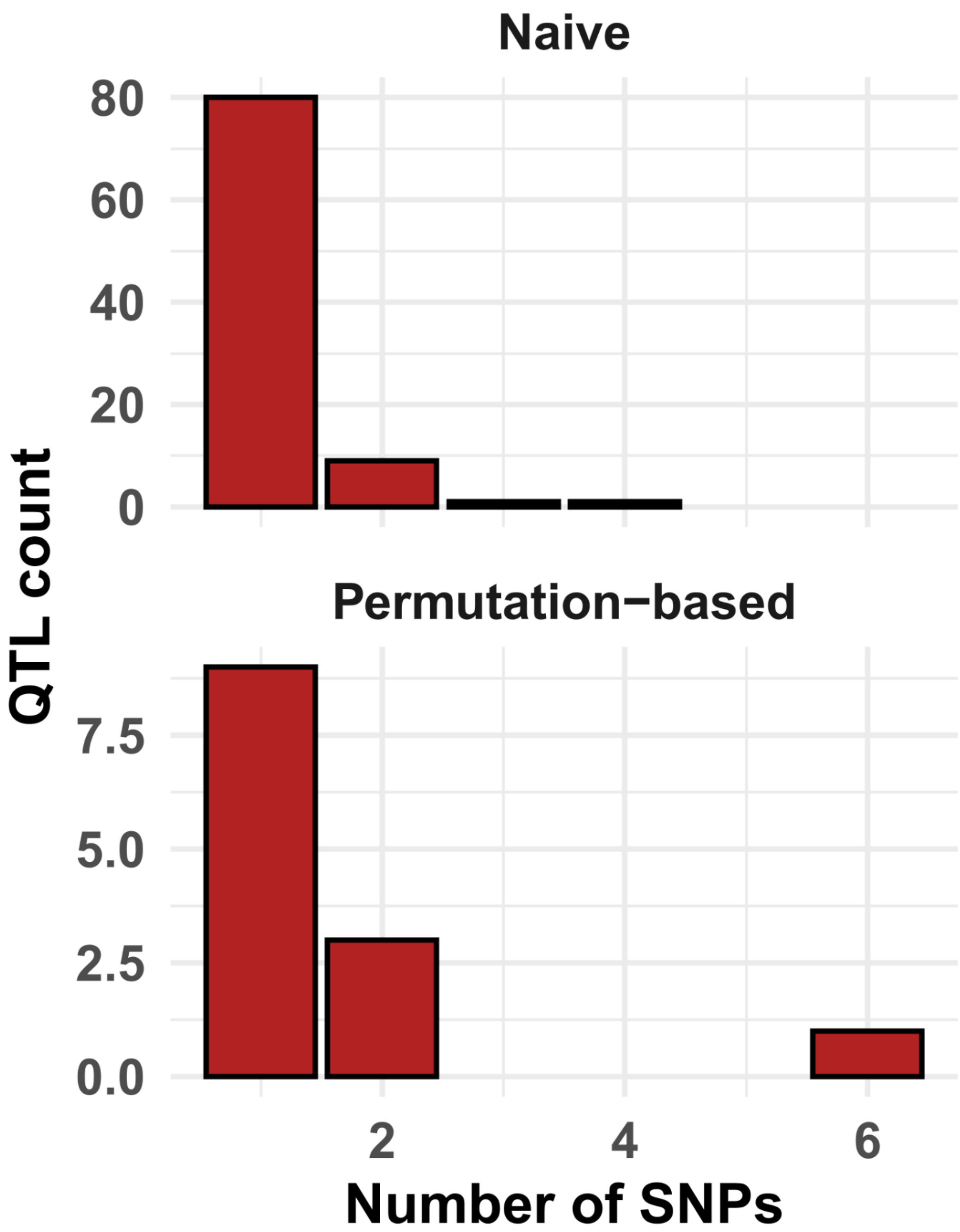

Extended data figure 13: Number of SNPs per QTL across different methods. Comparison of naïve and permutation-based results on the number of SNPs that were significant for each QTL.
