# Supplementary material for: Post-hoc permutation GWAS refinement reveals robust marker-trait associations in faba bean
Source: Front Plant Sci. 2026 Aug 10;17:1895808. doi: 10.3389/fpls.2026.1895808 (PMC13500635; doi:10.3389/fpls.2026.1895808)
Supplement: Supplementary file 2 [file DataSheet2.pdf]

Extended data figures 14-65: Scatterplots of GEMMA parametric p-values (unpermuted) and permutation-derived p-values. Scatterplots are for all trait and trial combinations except End of Flowering in Nordic Seed 2018, which is given in the paper. Traits are ordered alphabetically in this file.

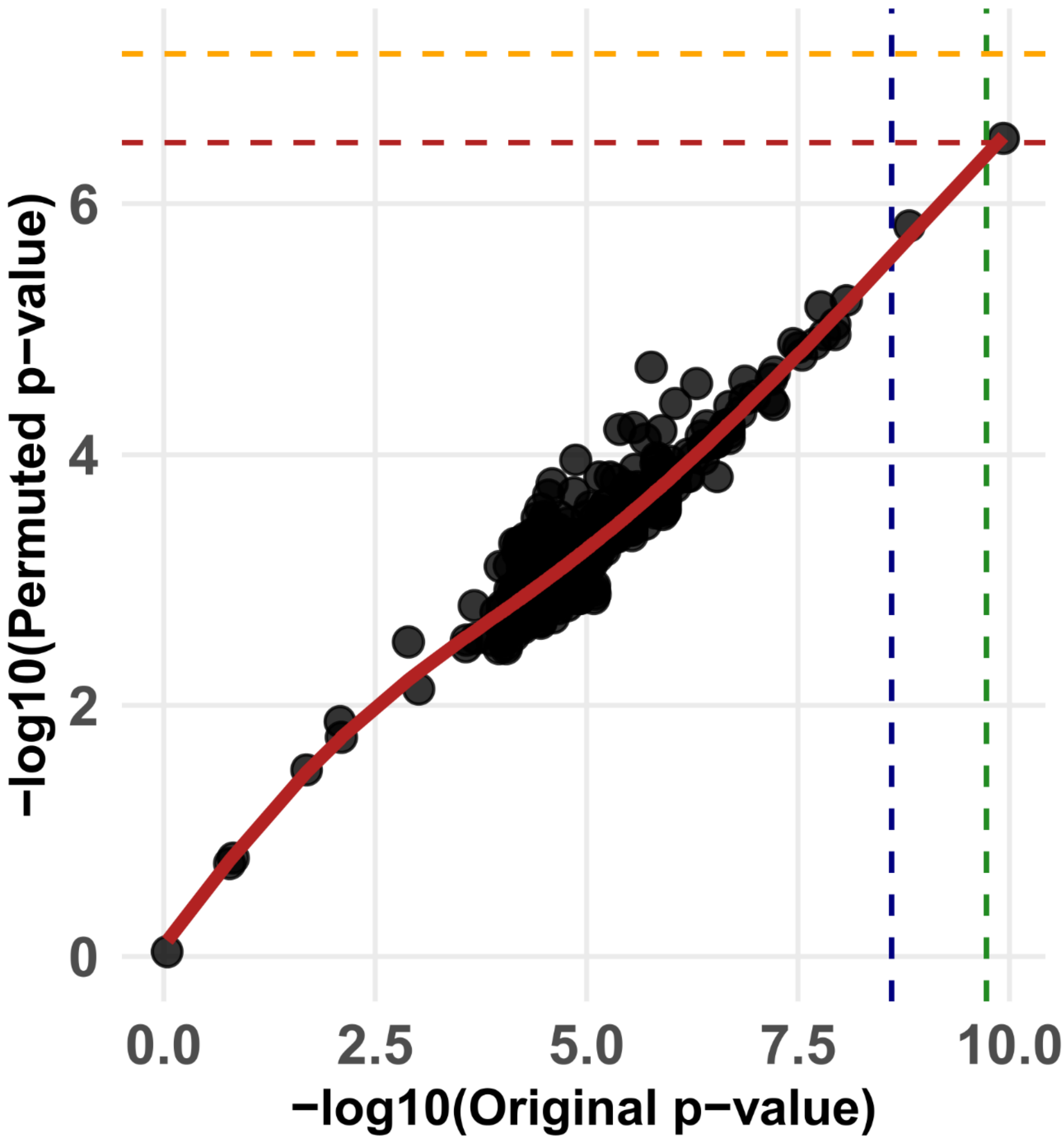

Extended data figure 14: Scatterplot of GEMMA parametric p-values (unpermuted) and permutation-derived p-values for Branching in Nordic Seed 2018. Only p-values originally below 10^{-4} in any of the three GWAS methods (FarmCPU, GEMMA, ISIS EM-BLASSO) are shown. Both p-value sets are -log10 transformed. To the points is fitted a smooth spline constrained to be monotonously increasing. 4 p-value thresholds are shown as dashed lines, the yellow and red horizontal ones are the 0.01 and 0.05 thresholds estimated using simpleM, while the green and blue vertical ones are the 0.01 and 0.05 thresholds estimated using PermGWAS2.

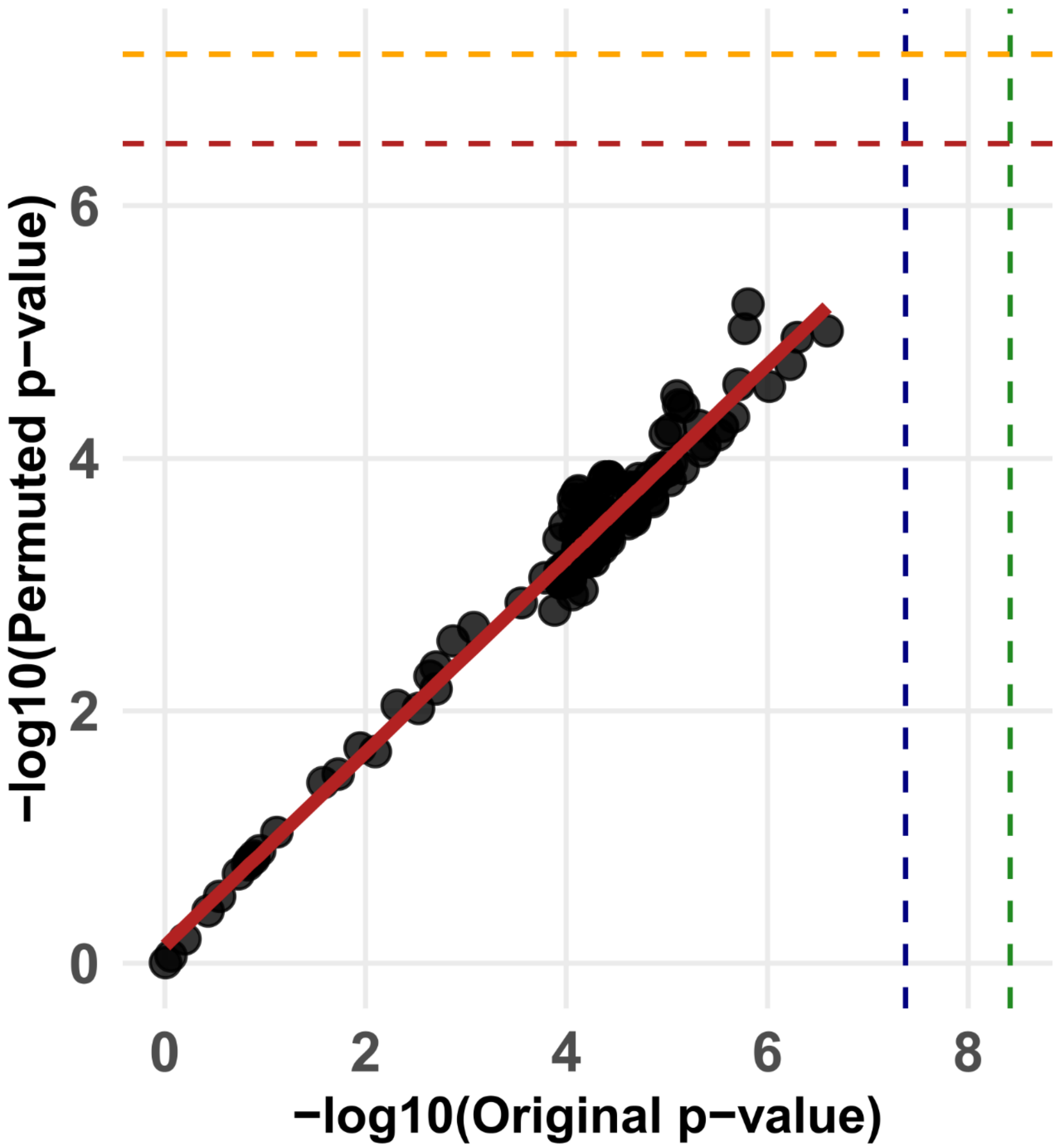

Extended data figure 15: Scatterplot of GEMMA parametric p-values (unpermuted) and permutation-derived p-values for Branching in Nordic Seed 2019. Only p-values originally below 10^{-4} in any of the three GWAS methods (FarmCPU, GEMMA, ISIS EM-BLASSO) are shown. Both p-value sets are -log10 transformed. To the points is fitted a smooth spline constrained to be monotonously increasing. 4 p-value thresholds are shown as dashed lines, the yellow and red horizontal ones are the 0.01 and 0.05 thresholds estimated using simpleM, while the green and blue vertical ones are the 0.01 and 0.05 thresholds estimated using PermGWAS2.

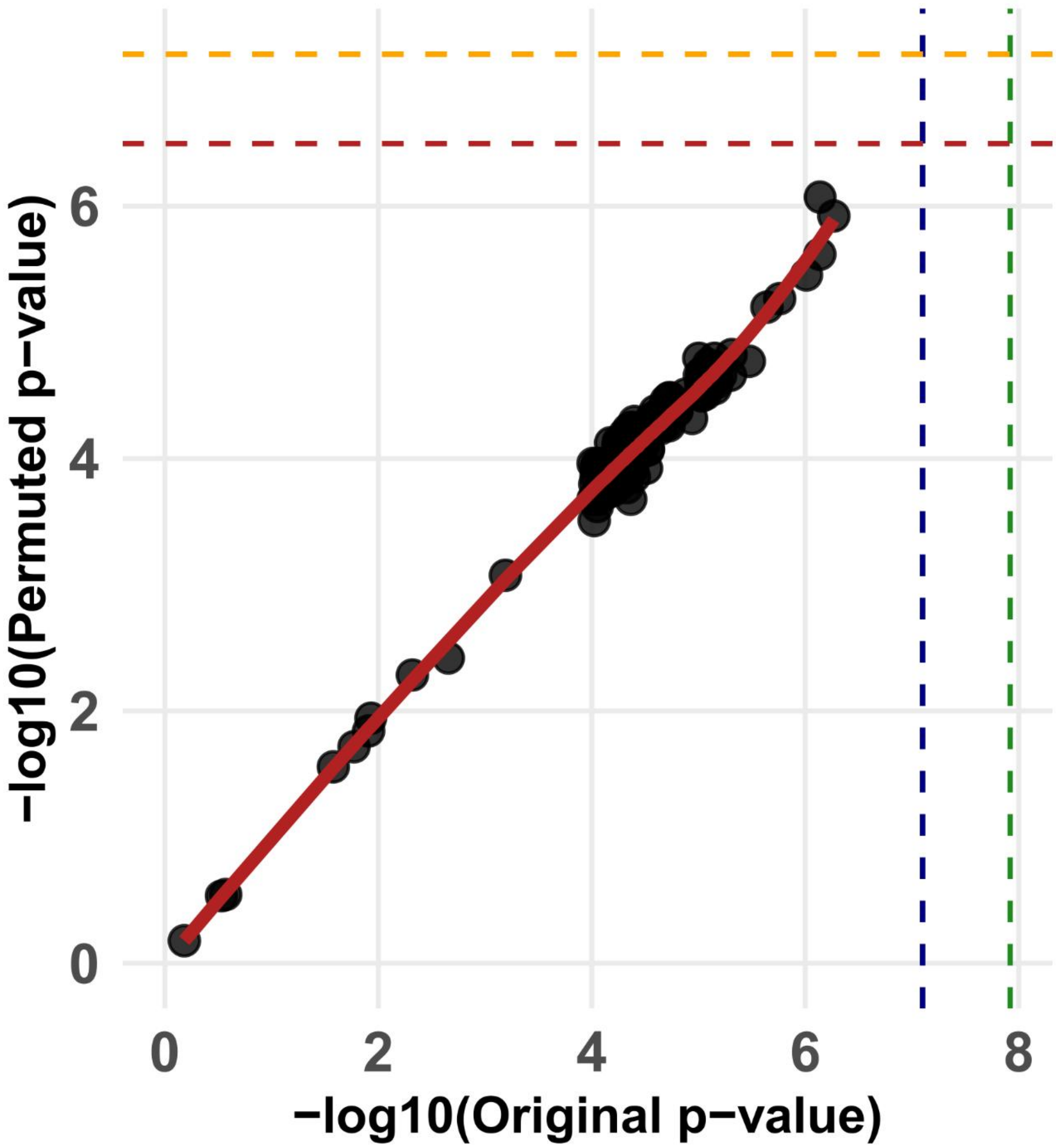

Extended data figure 16: Scatterplot of GEMMA parametric p-values (unpermuted) and permutation-derived p-values for Branching in Nordic Seed 2020. Only p-values originally below 10^{-4} in any of the three GWAS methods (FarmCPU, GEMMA, ISIS EM-BLASSO) are shown. Both p-value sets are -log10 transformed. To the points is fitted a smooth spline constrained to be monotonously increasing. 4 p-value thresholds are shown as dashed lines, the yellow and red horizontal ones are the 0.01 and 0.05 thresholds estimated using simpleM, while the green and blue vertical ones are the 0.01 and 0.05 thresholds estimated using PermGWAS2.

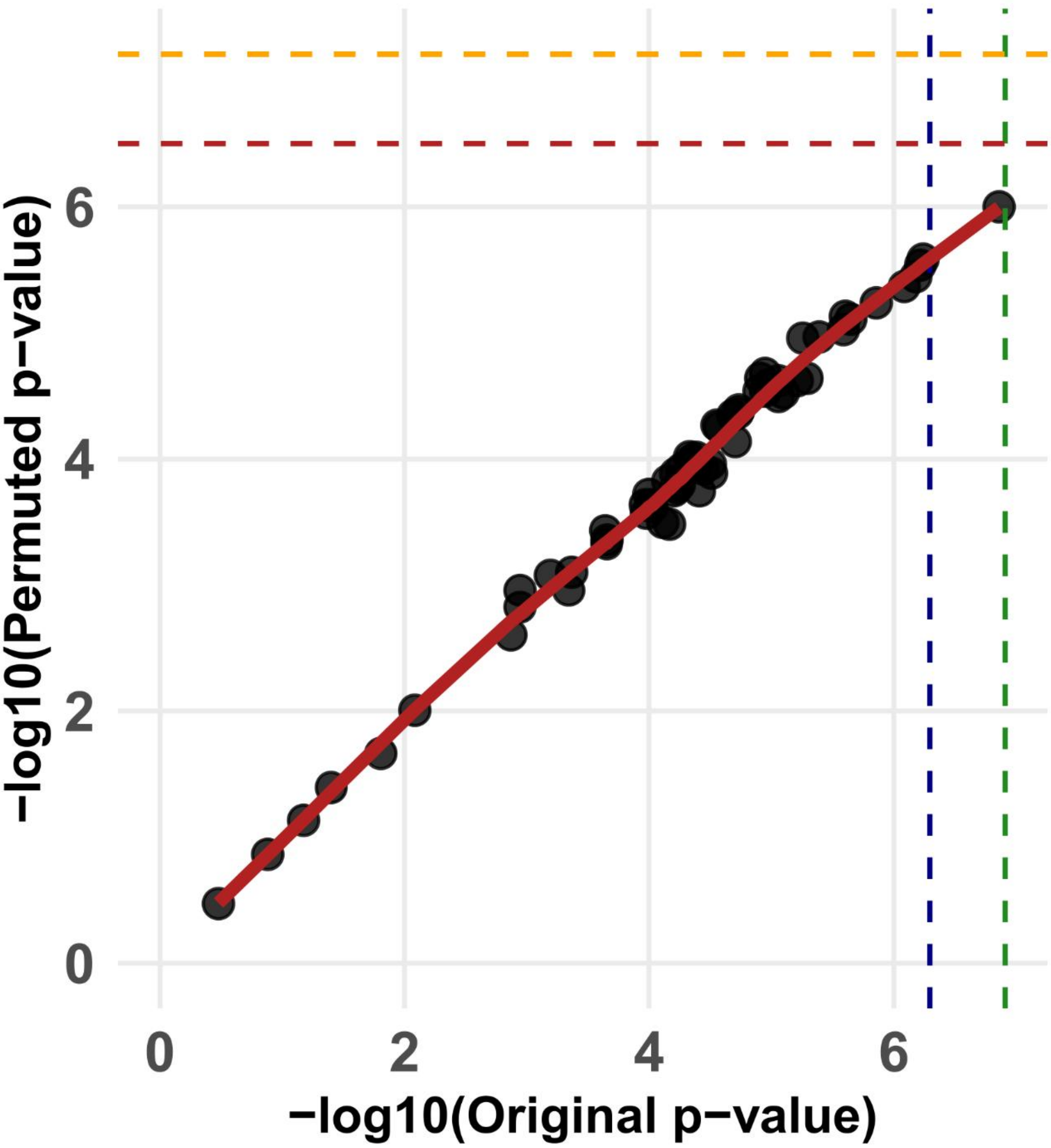

Extended data figure 17: Scatterplot of GEMMA parametric p-values (unpermuted) and permutation-derived p-values for Branching in Sejet 2019. Only p-values originally below 10^{-4} in any of the three GWAS methods (FarmCPU, GEMMA, ISIS EM-BLASSO) are shown. Both p-value sets are -log10 transformed. To the points is fitted a smooth spline constrained to be monotonously increasing. 4 p-value thresholds are shown as dashed lines, the yellow and red horizontal ones are the 0.01 and 0.05 thresholds estimated using simpleM, while the green and blue vertical ones are the 0.01 and 0.05 thresholds estimated using PermGWAS2.

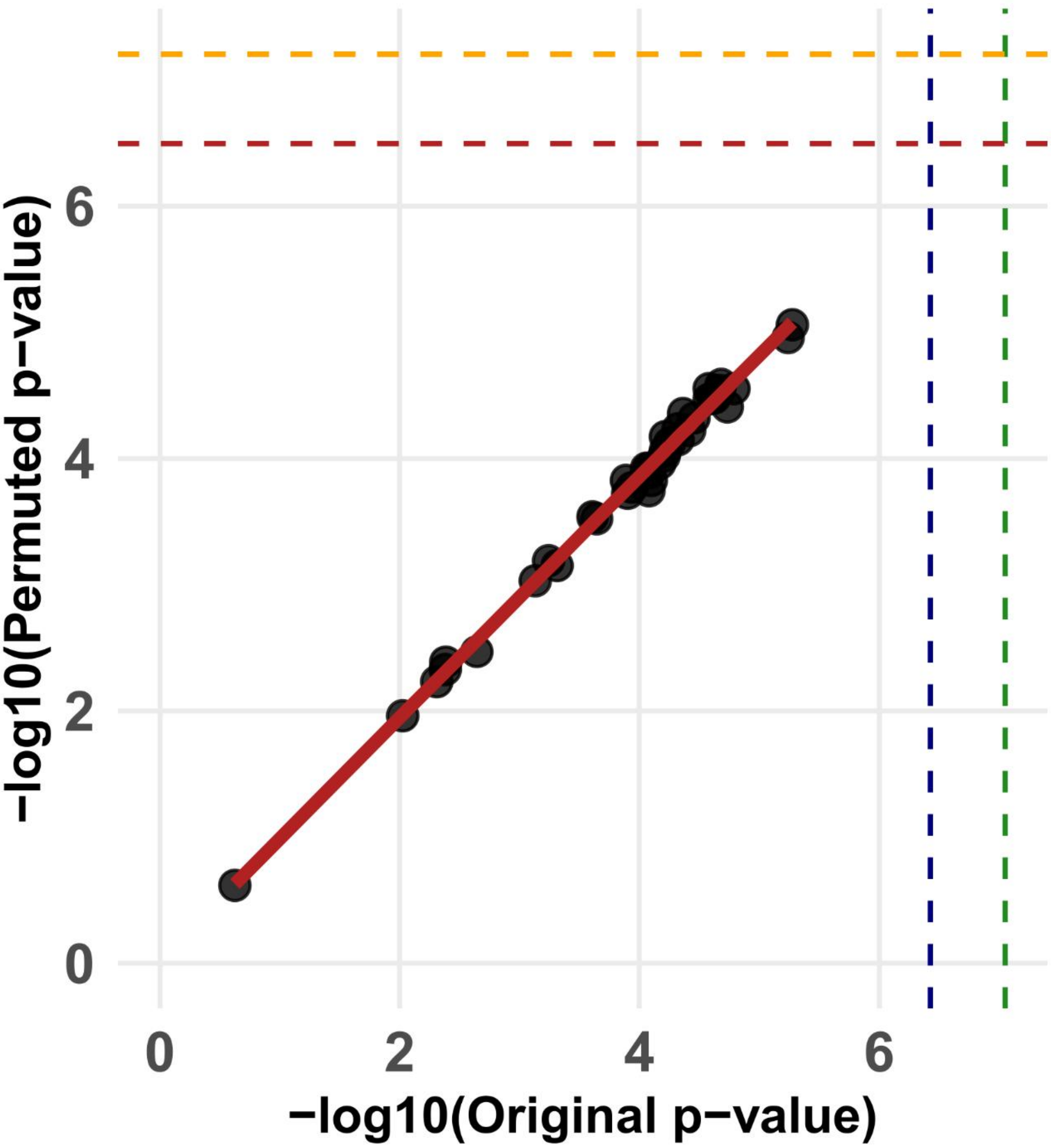

Extended data figure 18: Scatterplot of GEMMA parametric p-values (unpermuted) and permutation-derived p-values for Duration of Flowering (BLUEs across trials). Only p-values originally below 10^{-4} in any of the three GWAS methods (FarmCPU, GEMMA, ISIS EM-BLASSO) are shown. Both p-value sets are -log10 transformed. To the points is fitted a smooth spline constrained to be monotonously increasing. 4 p-value thresholds are shown as dashed lines, the yellow and red horizontal ones are the 0.01 and 0.05 thresholds estimated using simpleM, while the green and blue vertical ones are the 0.01 and 0.05 thresholds estimated using PermGWAS2.

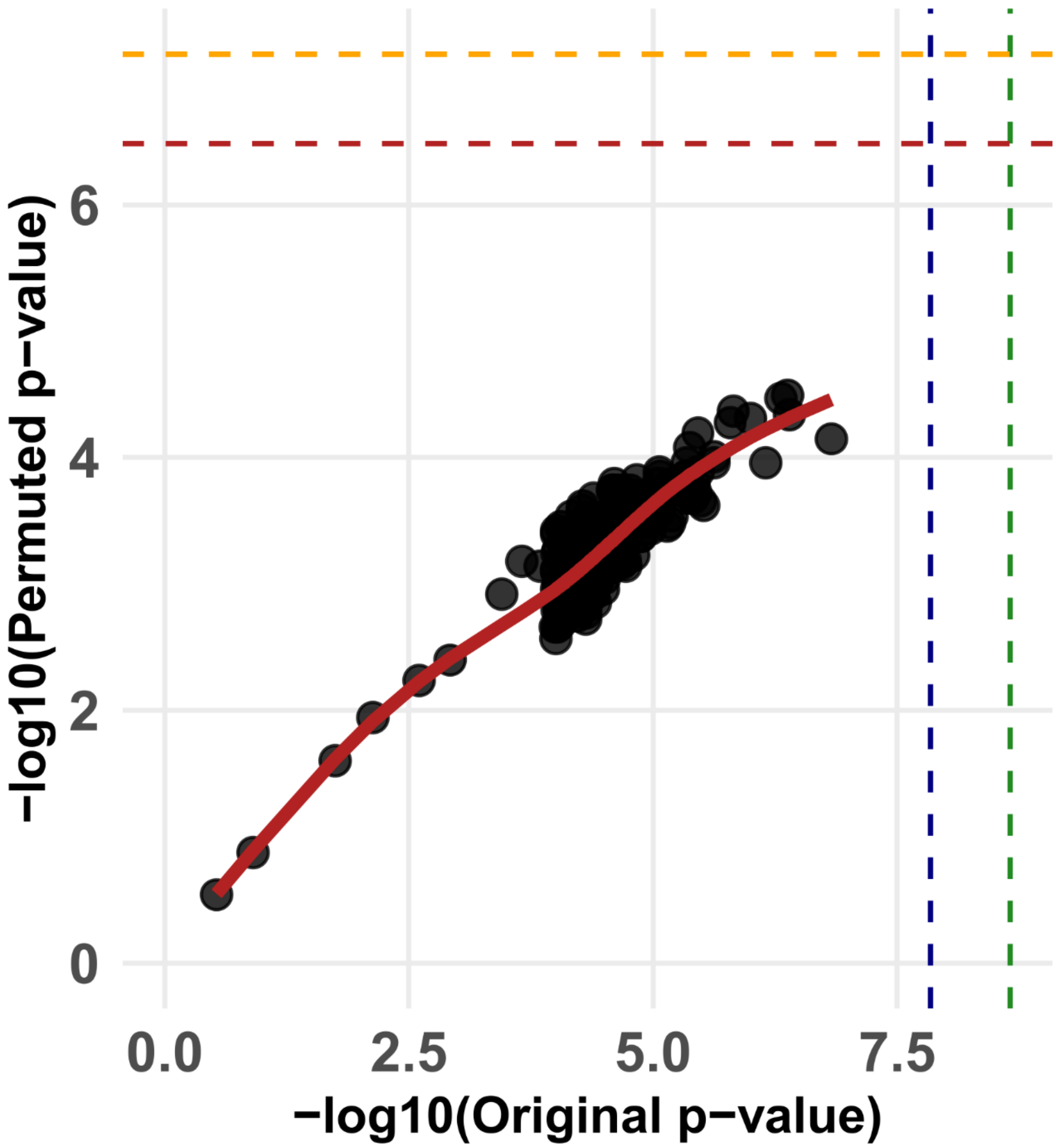

Extended data figure 19: Scatterplot of GEMMA parametric p-values (unpermuted) and permutation-derived p-values for Duration of Flowering in Nordic Seed 2018. Only p-values originally below 10^{-4} in any of the three GWAS methods (FarmCPU, GEMMA, ISIS EM-BLASSO) are shown. Both p-value sets are -log10 transformed. To the points is fitted a smooth spline constrained to be monotonously increasing. 4 p-value thresholds are shown as dashed lines, the yellow and red horizontal ones are the 0.01 and 0.05 thresholds estimated using simpleM, while the green and blue vertical ones are the 0.01 and 0.05 thresholds estimated using PermGWAS2.

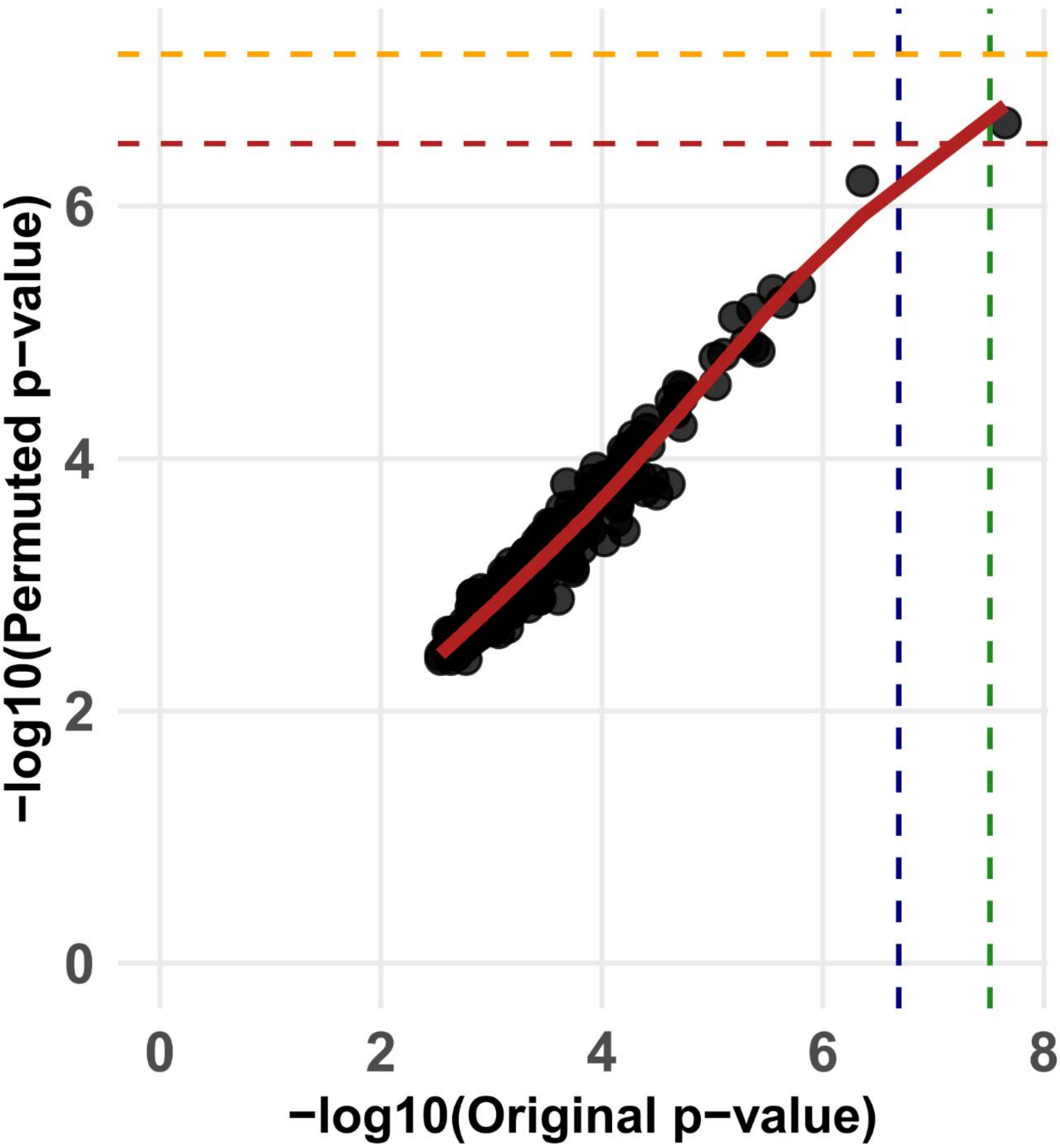

Extended data figure 20: Scatterplot of GEMMA parametric p-values (unpermuted) and permutation-derived p-values for Duration of Flowering in Nordic Seed 2019. Only p-values originally below 10^{-4} in any of the three GWAS methods (FarmCPU, GEMMA, ISIS EM-BLASSO) are shown. Both p-value sets are -log10 transformed. To the points is fitted a smooth spline constrained to be monotonously increasing. 4 p-value thresholds are shown as dashed lines, the yellow and red horizontal ones are the 0.01 and 0.05 thresholds estimated using simpleM, while the green and blue vertical ones are the 0.01 and 0.05 thresholds estimated using PermGWAS2.

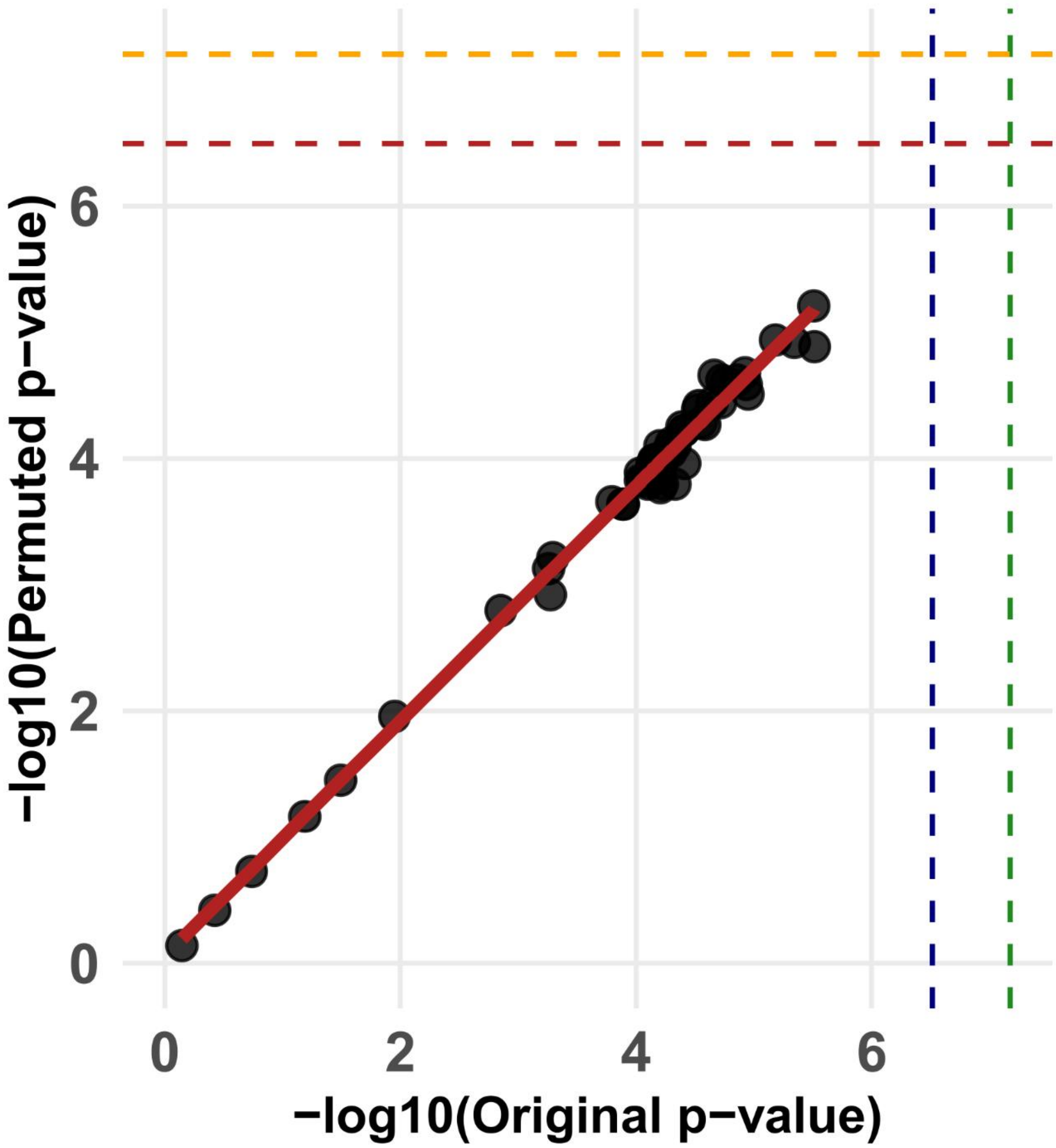

Extended data figure 21: Scatterplot of GEMMA parametric p-values (unpermuted) and permutation-derived p-values for Duration of Flowering in Nordic Seed 2020. Only p-values originally below 10^{-4} in any of the three GWAS methods (FarmCPU, GEMMA, ISIS EM-BLASSO) are shown. Both p-value sets are -log10 transformed. To the points is fitted a smooth spline constrained to be monotonously increasing. 4 p-value thresholds are shown as dashed lines, the yellow and red horizontal ones are the 0.01 and 0.05 thresholds estimated using simpleM, while the green and blue vertical ones are the 0.01 and 0.05 thresholds estimated using PermGWAS2.

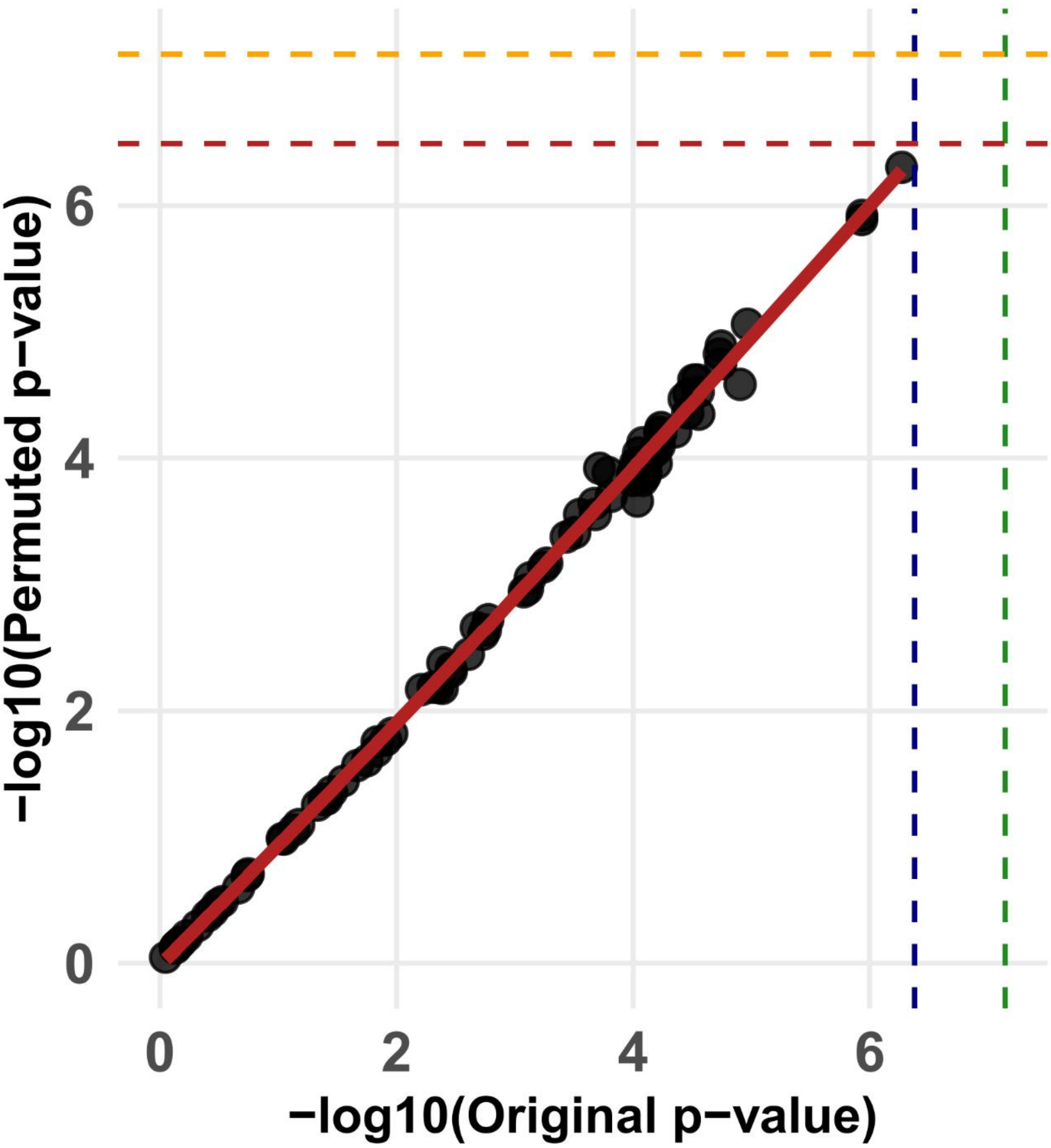

Extended data figure 22: Scatterplot of GEMMA parametric p-values (unpermuted) and permutation-derived p-values for Earliness of Flowering (BLUEs across trials). Only p-values originally below 10^{-4} in any of the three GWAS methods (FarmCPU, GEMMA, ISIS EM-BLASSO) are shown. Both p-value sets are -log10 transformed. To the points is fitted a smooth spline constrained to be monotonously increasing. 4 p-value thresholds are shown as dashed lines, the yellow and red horizontal ones are the 0.01 and 0.05 thresholds estimated using simpleM, while the green and blue vertical ones are the 0.01 and 0.05 thresholds estimated using PermGWAS2.

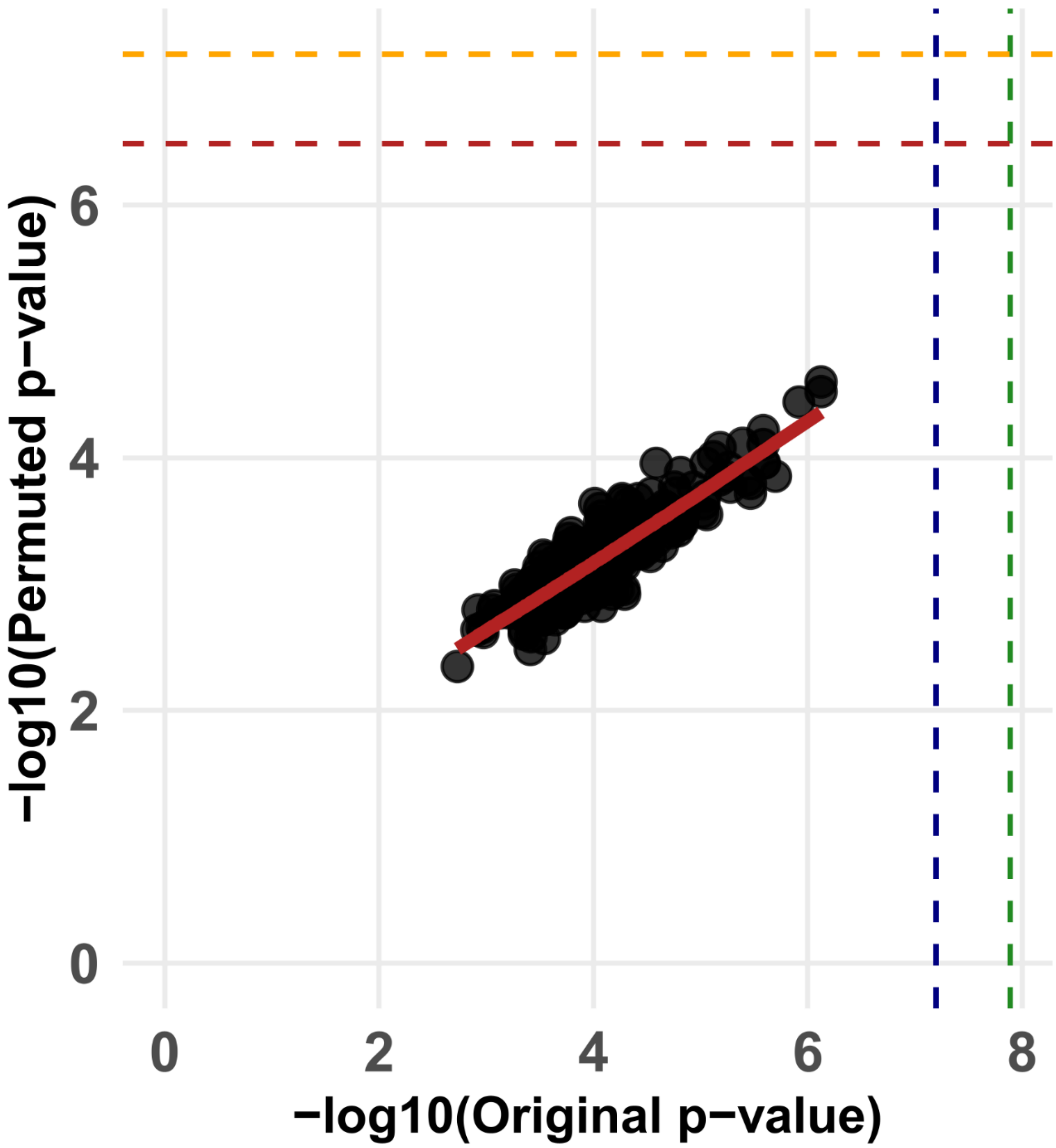

Extended data figure 23: Scatterplot of GEMMA parametric p-values (unpermuted) and permutation-derived p-values for Earliness of Flowering in Nordic Seed 2018. Only p-values originally below 10^{-4} in any of the three GWAS methods (FarmCPU, GEMMA, ISIS EM-BLASSO) are shown. Both p-value sets are -log10 transformed. To the points is fitted a smooth spline constrained to be monotonously increasing. 4 p-value thresholds are shown as dashed lines, the yellow and red horizontal ones are the 0.01 and 0.05 thresholds estimated using simpleM, while the green and blue vertical ones are the 0.01 and 0.05 thresholds estimated using PermGWAS2.

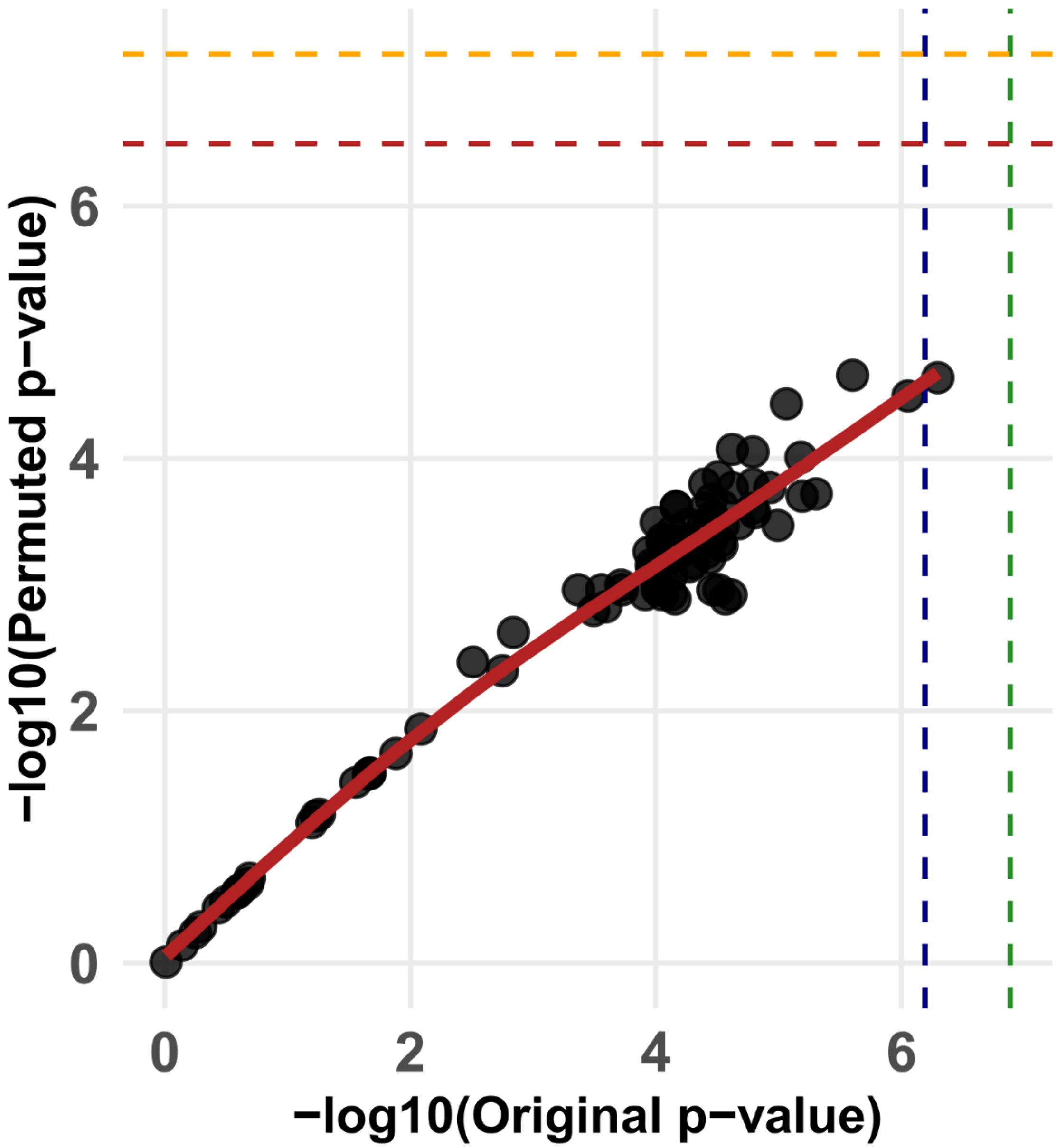

Extended data figure 24: Scatterplot of GEMMA parametric p-values (unpermuted) and permutation-derived p-values for Earliness of Flowering in Nordic Seed 2019. Only p-values originally below 10^{-4} in any of the three GWAS methods (FarmCPU, GEMMA, ISIS EM-BLASSO) are shown. Both p-value sets are -log10 transformed. To the points is fitted a smooth spline constrained to be monotonously increasing. 4 p-value thresholds are shown as dashed lines, the yellow and red horizontal ones are the 0.01 and 0.05 thresholds estimated using simpleM, while the green and blue vertical ones are the 0.01 and 0.05 thresholds estimated using PermGWAS2.

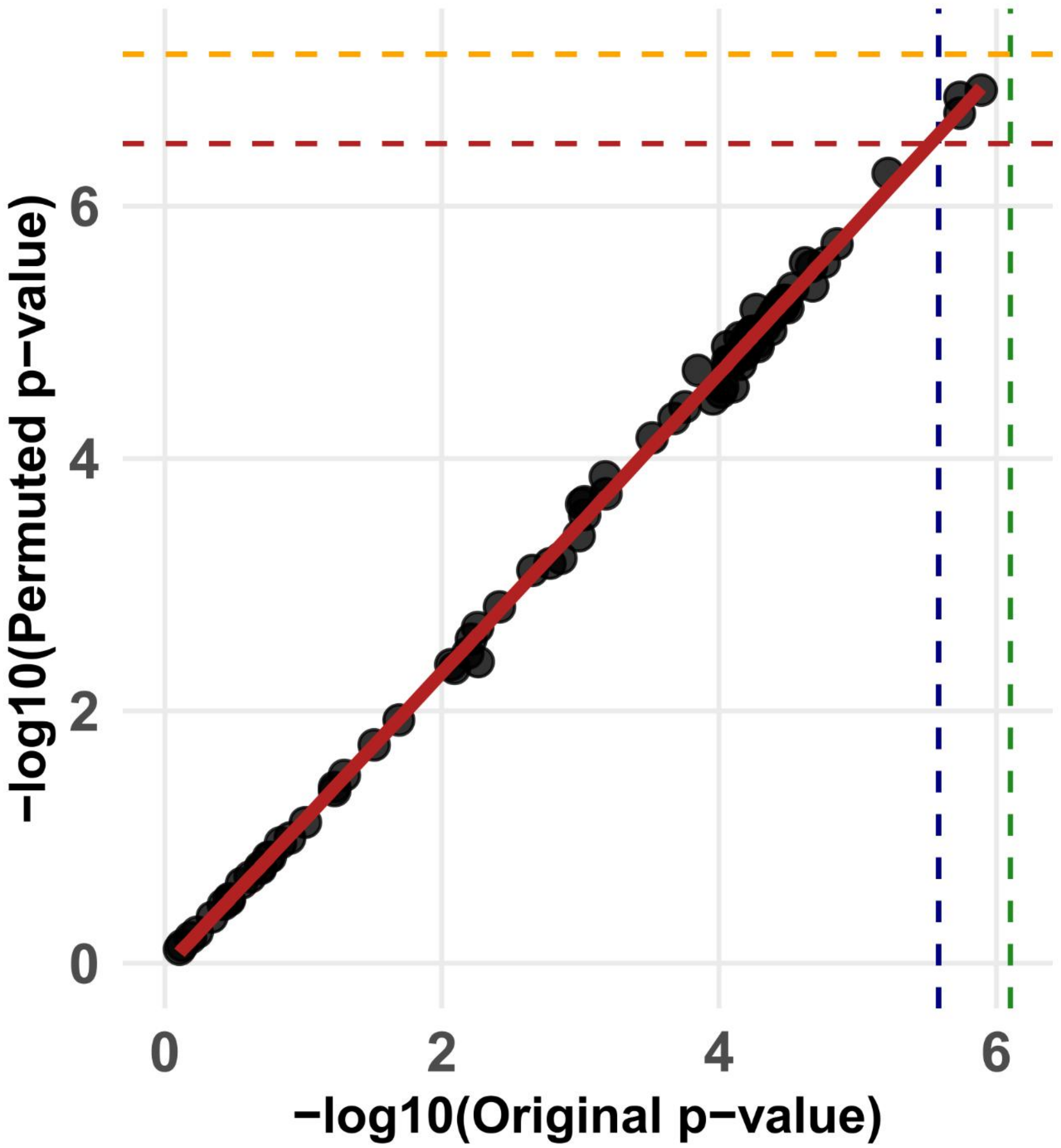

Extended data figure 25: Scatterplot of GEMMA parametric p-values (unpermuted) and permutation-derived p-values for Earliness of Flowering in Nordic Seed 2020. Only p-values originally below 10^{-4} in any of the three GWAS methods (FarmCPU, GEMMA, ISIS EM-BLASSO) are shown. Both p-value sets are -log10 transformed. To the points is fitted a smooth spline constrained to be monotonously increasing. 4 p-value thresholds are shown as dashed lines, the yellow and red horizontal ones are the 0.01 and 0.05 thresholds estimated using simpleM, while the green and blue vertical ones are the 0.01 and 0.05 thresholds estimated using PermGWAS2.

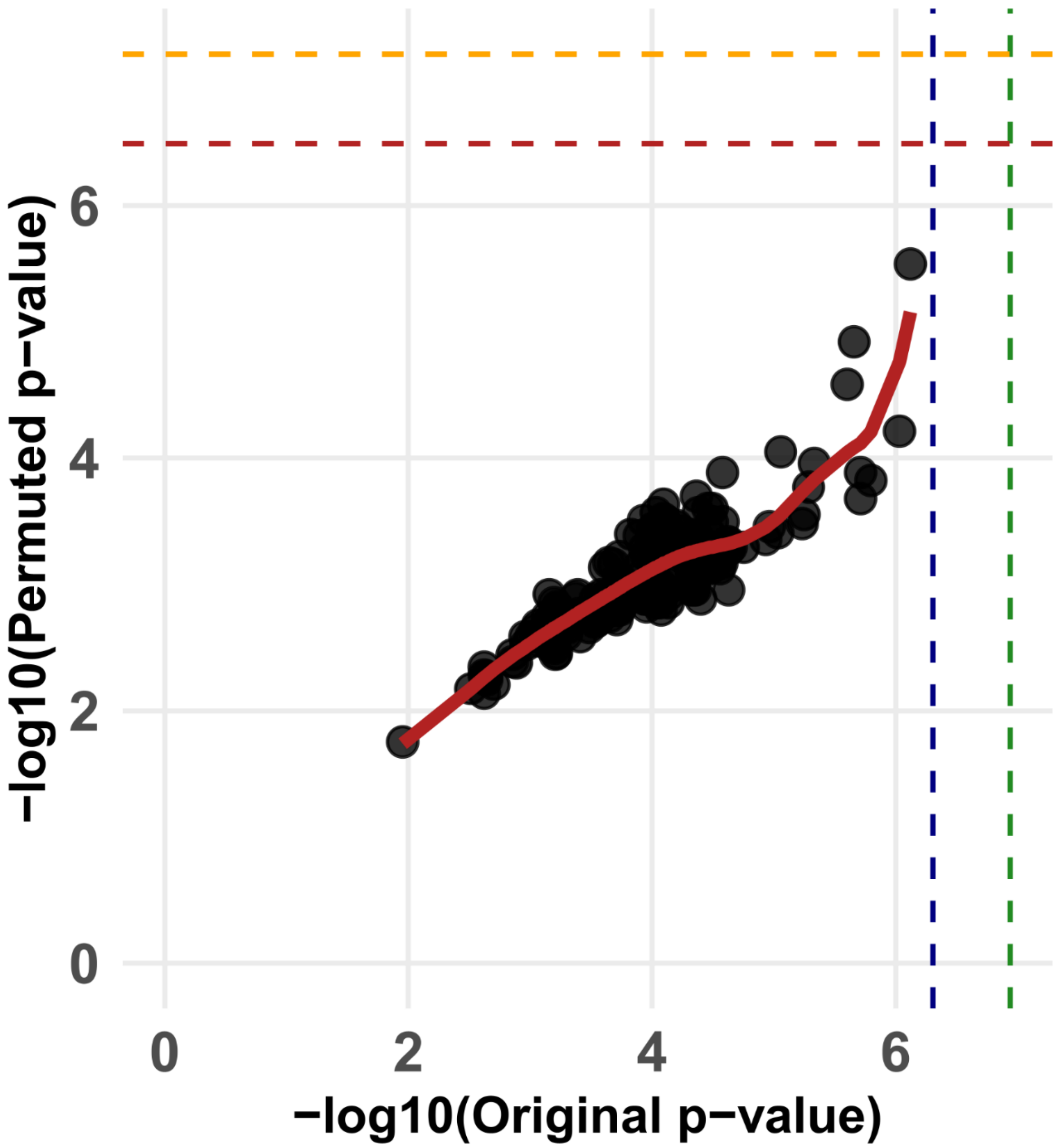

Extended data figure 26: Scatterplot of GEMMA parametric p-values (unpermuted) and permutation-derived p-values for Earliness of Flowering in Sejet 2018. Only p-values originally below 10^{-4} in any of the three GWAS methods (FarmCPU, GEMMA, ISIS EM-BLASSO) are shown. Both p-value sets are -log10 transformed. To the points is fitted a smooth spline constrained to be monotonously increasing. 4 p-value thresholds are shown as dashed lines, the yellow and red horizontal ones are the 0.01 and 0.05 thresholds estimated using simpleM, while the green and blue vertical ones are the 0.01 and 0.05 thresholds estimated using PermGWAS2.

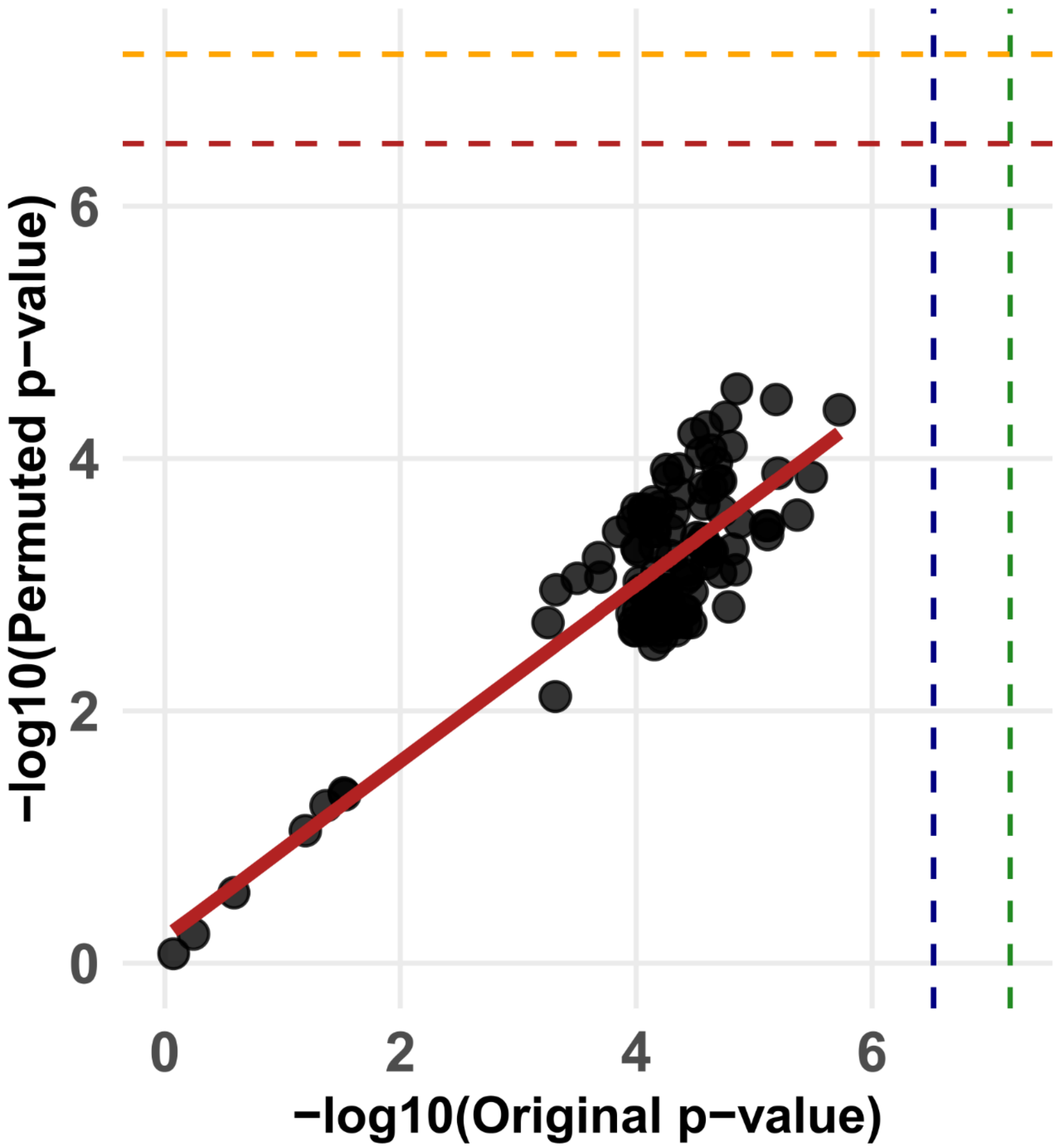

Extended data figure 27: Scatterplot of GEMMA parametric p-values (unpermuted) and permutation-derived p-values for Earliness of Flowering in Sejet 2019. Only p-values originally below 10^{-4} in any of the three GWAS methods (FarmCPU, GEMMA, ISIS EM-BLASSO) are shown. Both p-value sets are -log10 transformed. To the points is fitted a smooth spline constrained to be monotonously increasing. 4 p-value thresholds are shown as dashed lines, the yellow and red horizontal ones are the 0.01 and 0.05 thresholds estimated using simpleM, while the green and blue vertical ones are the 0.01 and 0.05 thresholds estimated using PermGWAS2.

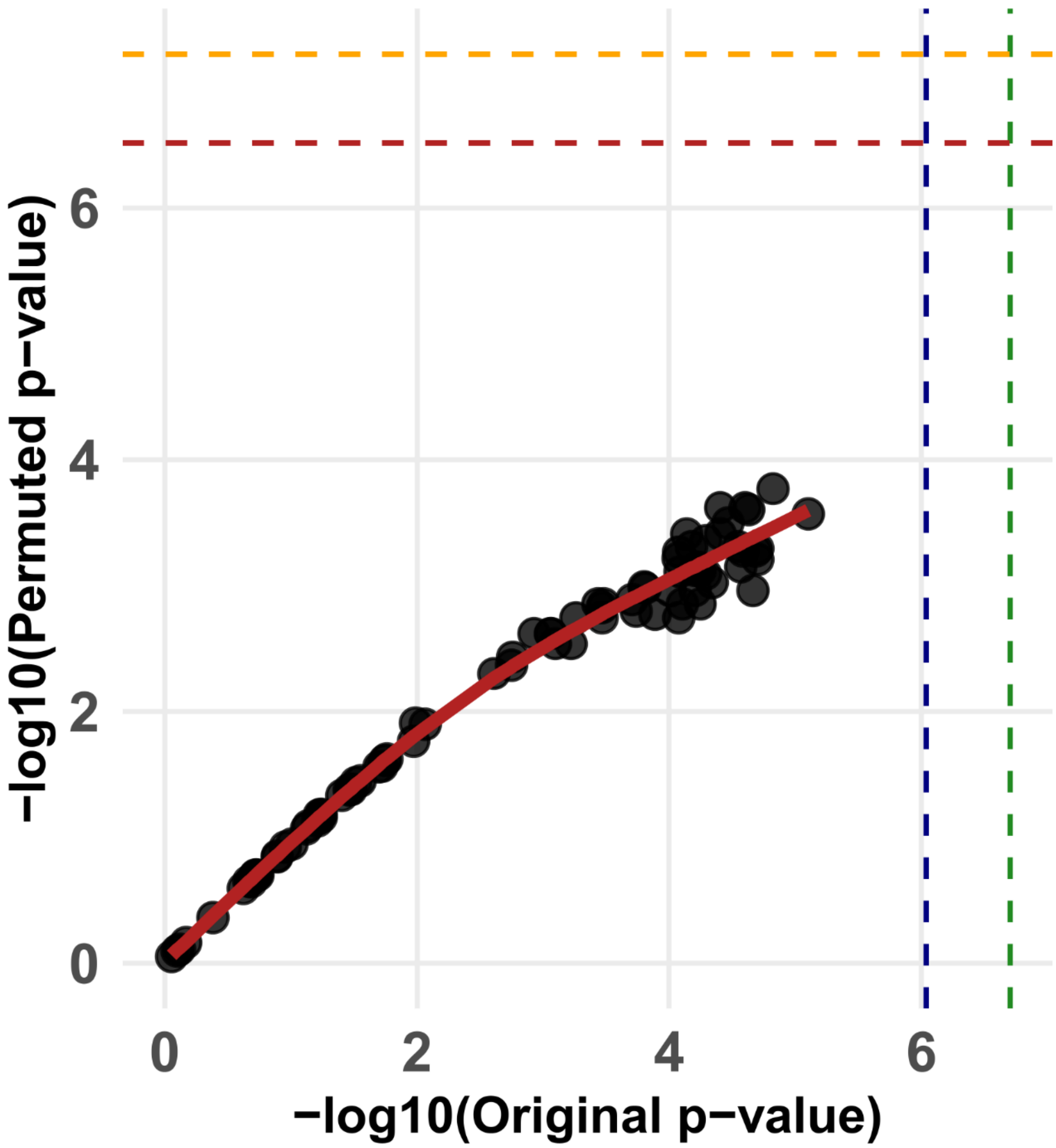

Extended data figure 28: Scatterplot of GEMMA parametric p-values (unpermuted) and permutation-derived p-values for Earliness of Flowering in Sejet 2021. Only p-values originally below 10^{-4} in any of the three GWAS methods (FarmCPU, GEMMA, ISIS EM-BLASSO) are shown. Both p-value sets are -log10 transformed. To the points is fitted a smooth spline constrained to be monotonously increasing. 4 p-value thresholds are shown as dashed lines, the yellow and red horizontal ones are the 0.01 and 0.05 thresholds estimated using simpleM, while the green and blue vertical ones are the 0.01 and 0.05 thresholds estimated using PermGWAS2.

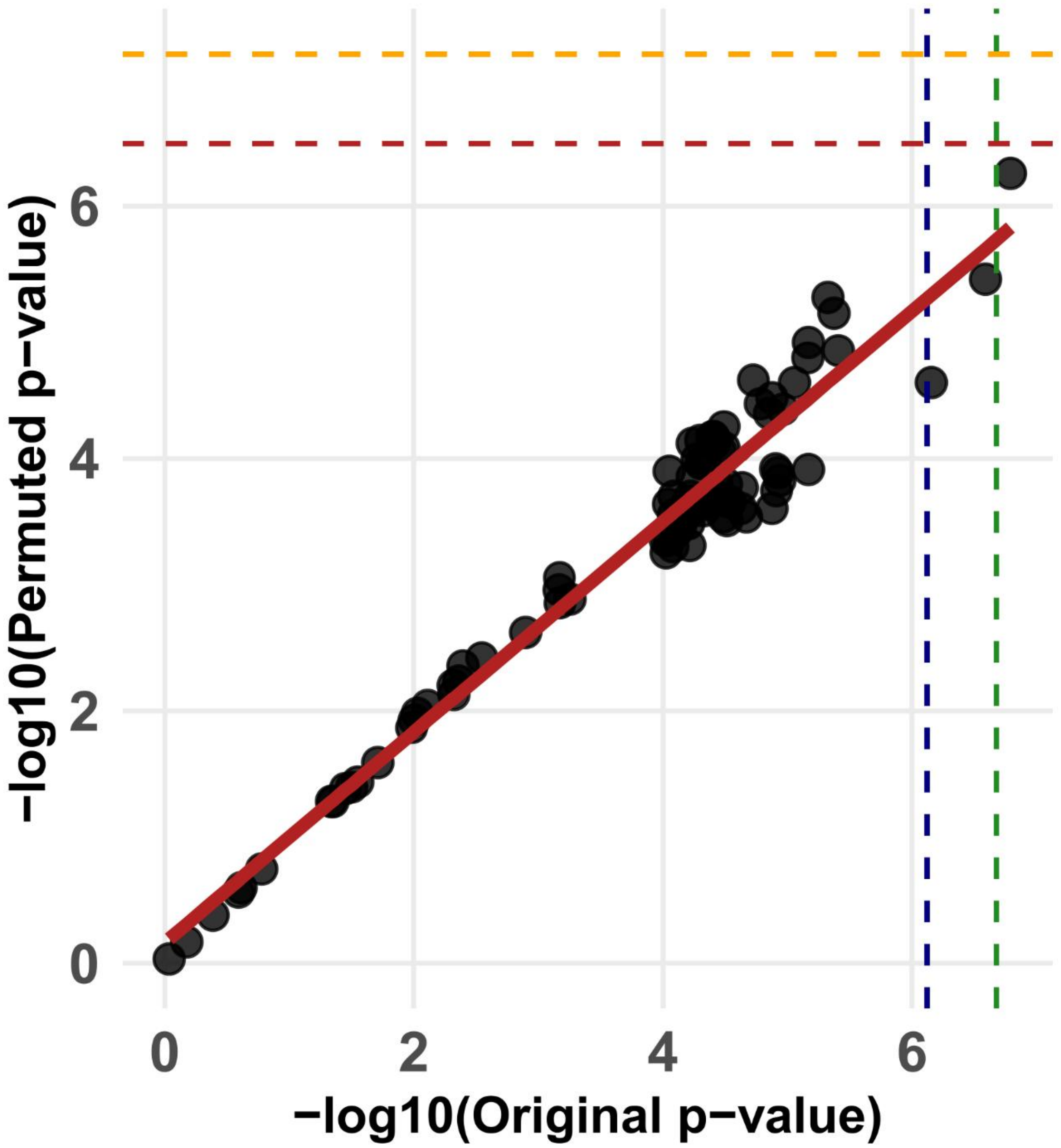

Extended data figure 29: Scatterplot of GEMMA parametric p-values (unpermuted) and permutation-derived p-values for End Flowering (BLUEs across trials). Only p-values originally below 10^{-4} in any of the three GWAS methods (FarmCPU, GEMMA, ISIS EM-BLASSO) are shown. Both p-value sets are -log10 transformed. To the points is fitted a smooth spline constrained to be monotonously increasing. 4 p-value thresholds are shown as dashed lines, the yellow and red horizontal ones are the 0.01 and 0.05 thresholds estimated using simpleM, while the green and blue vertical ones are the 0.01 and 0.05 thresholds estimated using PermGWAS2.

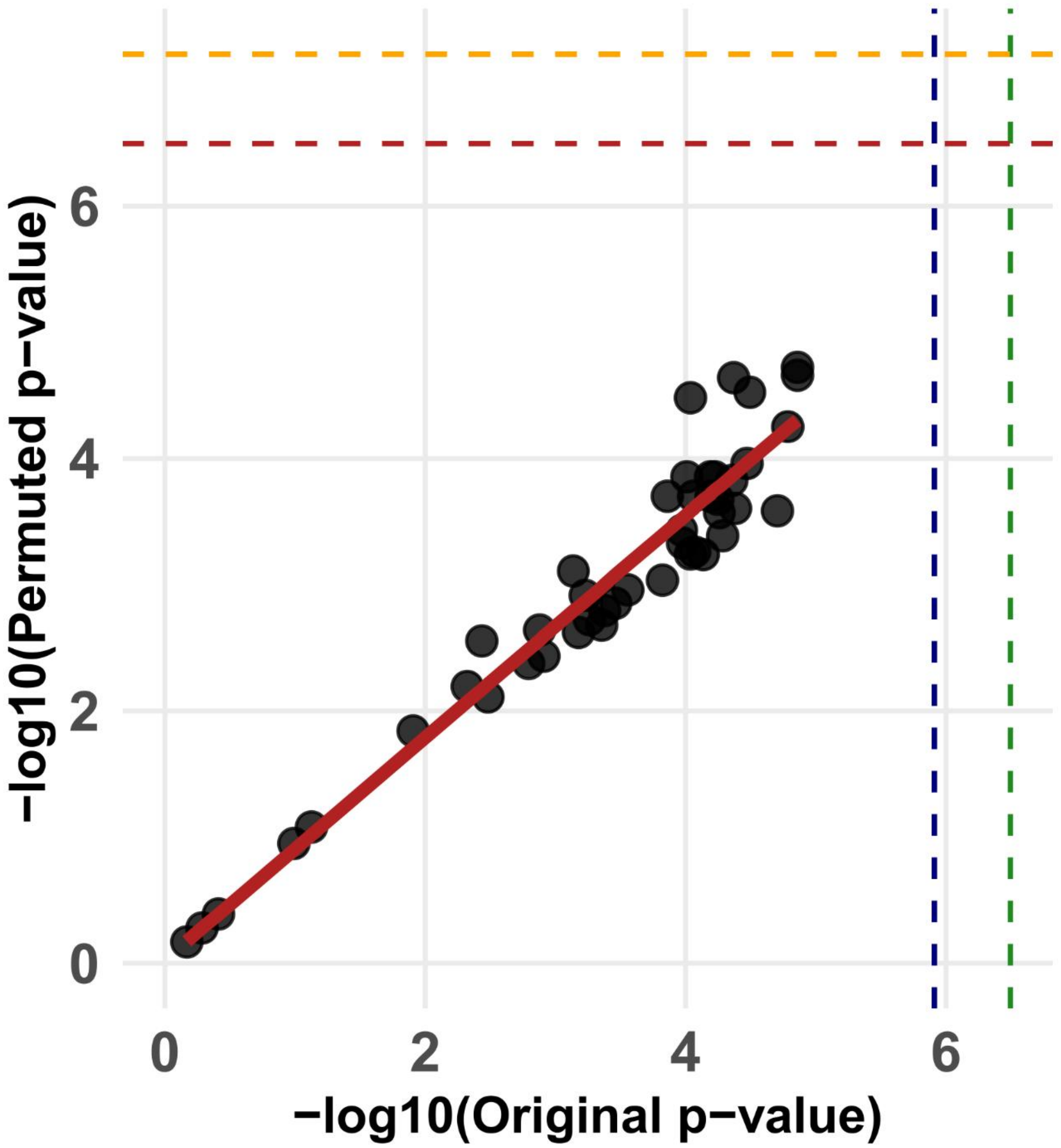

Extended data figure 30: Scatterplot of GEMMA parametric p-values (unpermuted) and permutation-derived p-values for End Flowering in Nordic Seed 2019. Only p-values originally below 10^{-4} in any of the three GWAS methods (FarmCPU, GEMMA, ISIS EM-BLASSO) are shown. Both p-value sets are -log10 transformed. To the points is fitted a smooth spline constrained to be monotonously increasing. 4 p-value thresholds are shown as dashed lines, the yellow and red horizontal ones are the 0.01 and 0.05 thresholds estimated using simpleM, while the green and blue vertical ones are the 0.01 and 0.05 thresholds estimated using PermGWAS2.

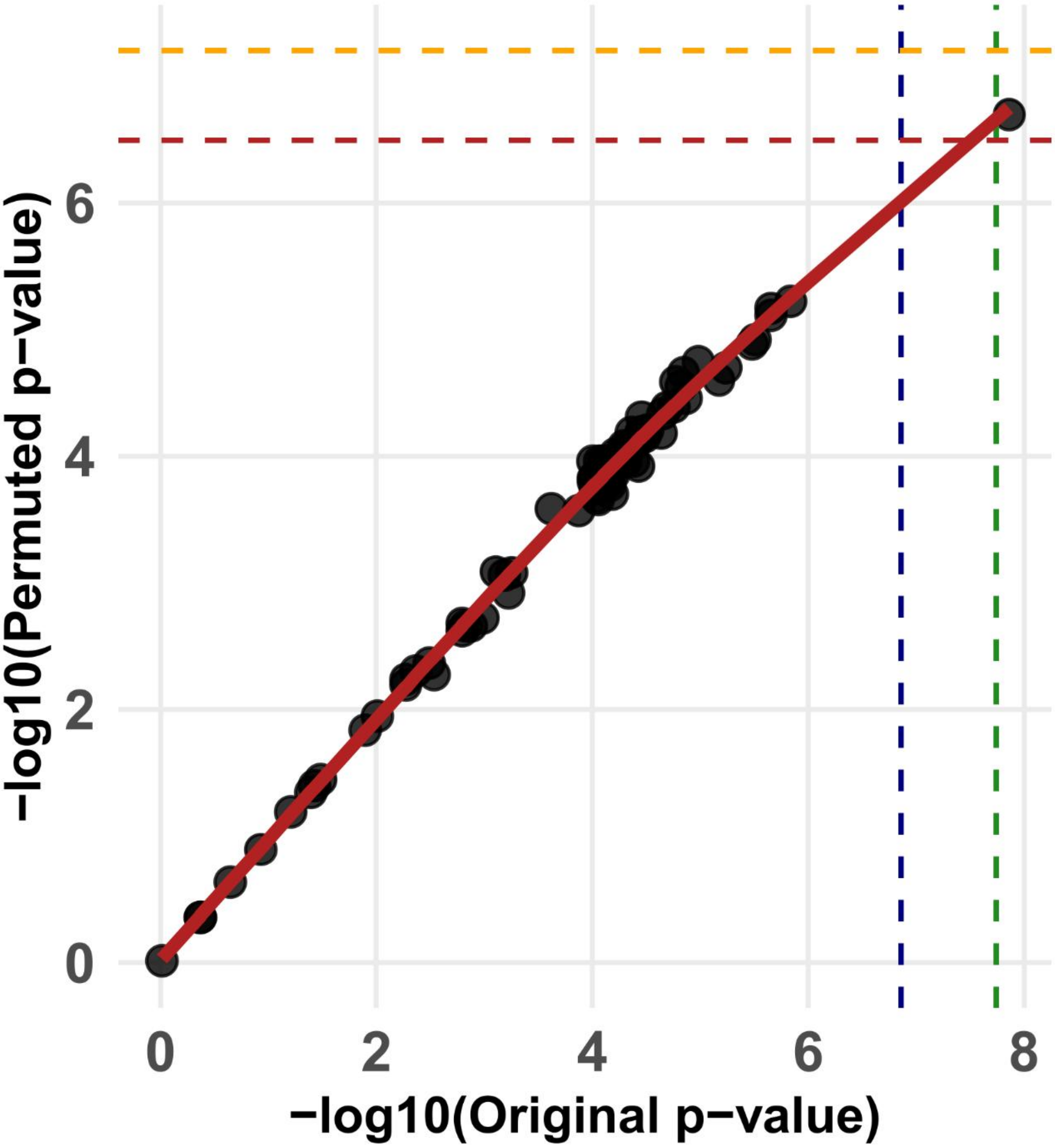

Extended data figure 31: Scatterplot of GEMMA parametric p-values (unpermuted) and permutation-derived p-values for End Flowering in Nordic Seed 2020. Only p-values originally below 10^{-4} in any of the three GWAS methods (FarmCPU, GEMMA, ISIS EM-BLASSO) are shown. Both p-value sets are -log10 transformed. To the points is fitted a smooth spline constrained to be monotonously increasing. 4 p-value thresholds are shown as dashed lines, the yellow and red horizontal ones are the 0.01 and 0.05 thresholds estimated using simpleM, while the green and blue vertical ones are the 0.01 and 0.05 thresholds estimated using PermGWAS2.

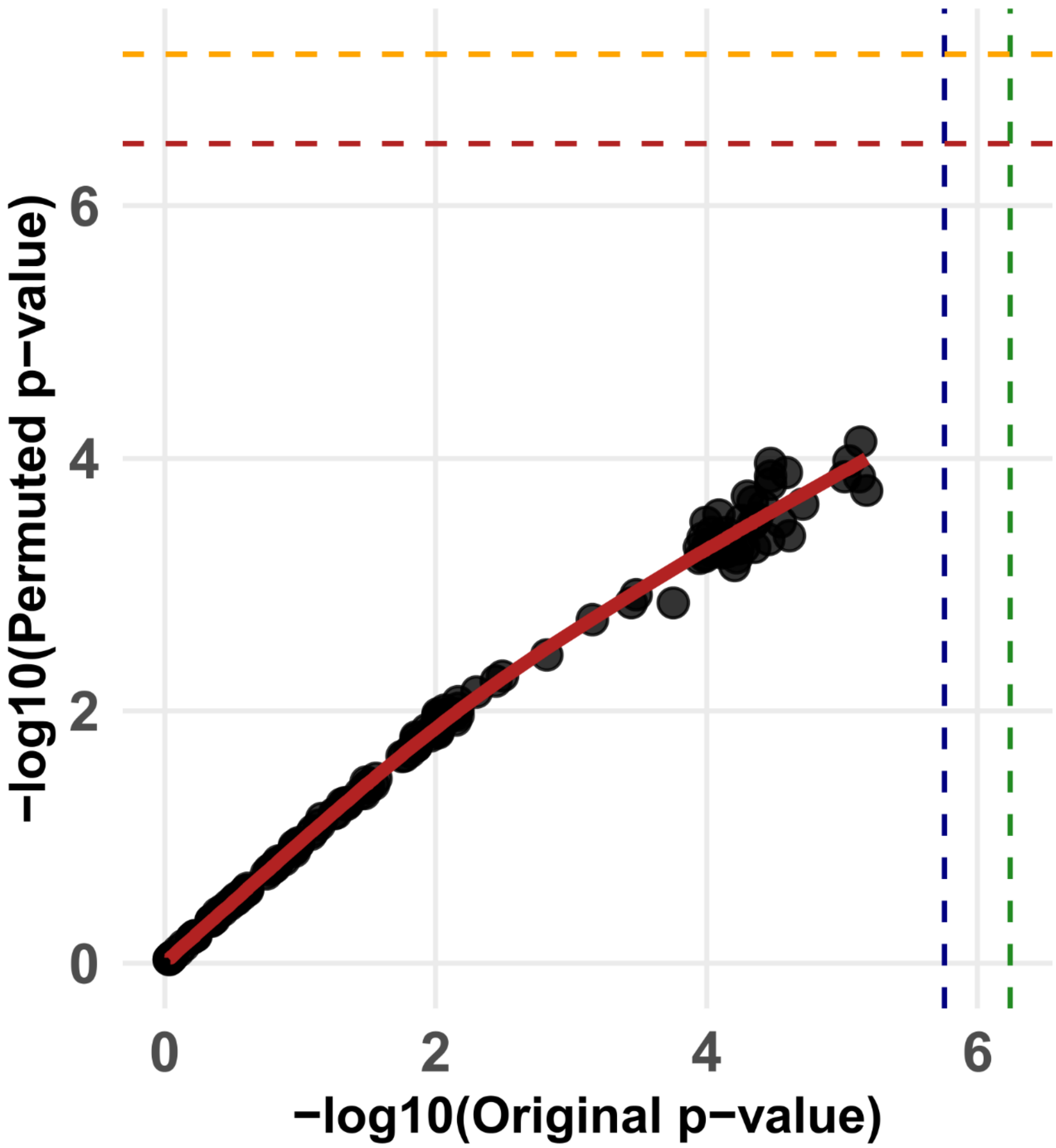

Extended data figure 32: Scatterplot of GEMMA parametric p-values (unpermuted) and permutation-derived p-values for Internode Length in Nordic Seed 2019. Only p-values originally below 10^{-4} in any of the three GWAS methods (FarmCPU, GEMMA, ISIS EM-BLASSO) are shown. Both p-value sets are -log10 transformed. To the points is fitted a smooth spline constrained to be monotonously increasing. 4 p-value thresholds are shown as dashed lines, the yellow and red horizontal ones are the 0.01 and 0.05 thresholds estimated using simpleM, while the green and blue vertical ones are the 0.01 and 0.05 thresholds estimated using PermGWAS2.

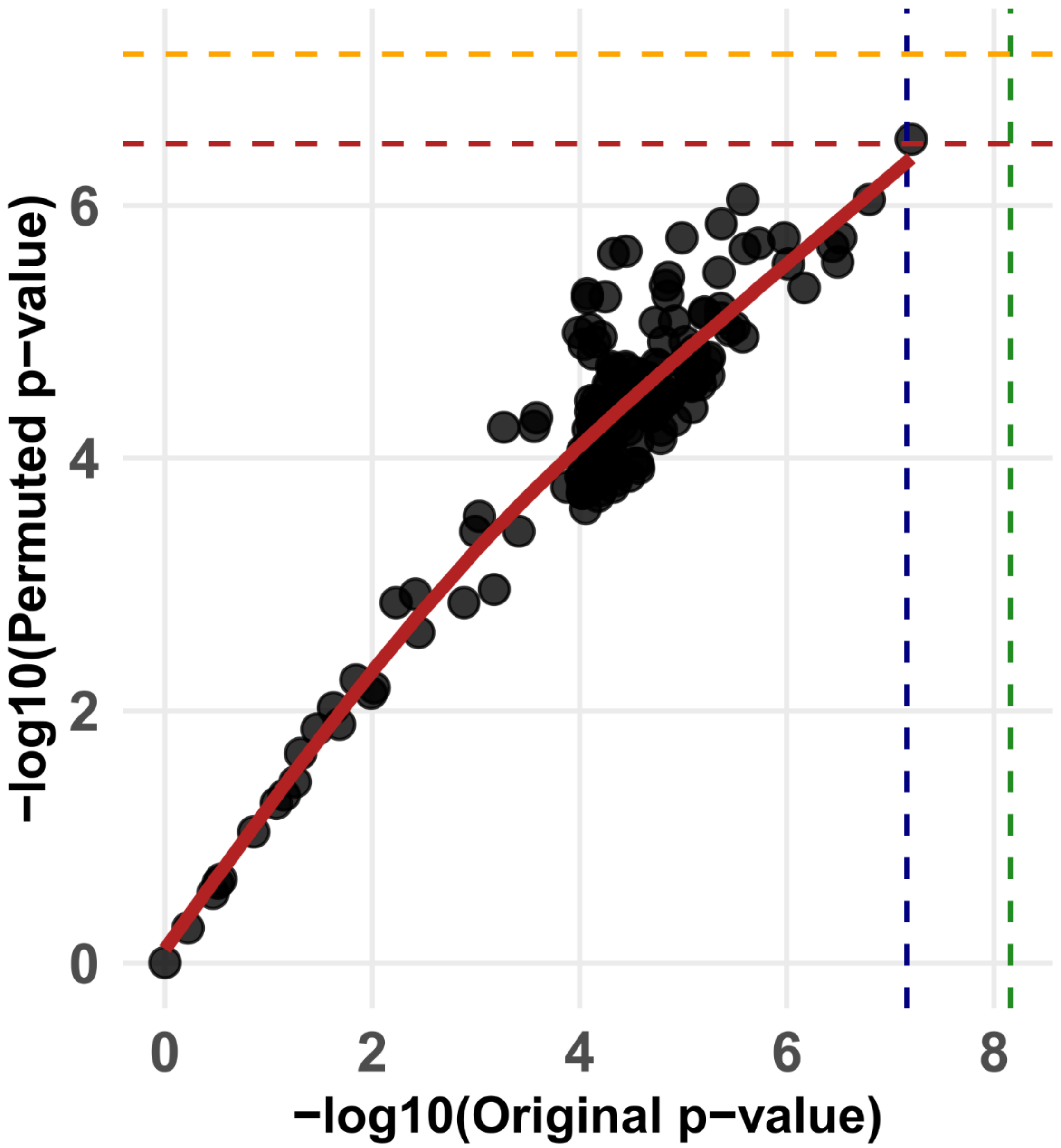

Extended data figure 33: Scatterplot of GEMMA parametric p-values (unpermuted) and permutation-derived p-values for Lodging (BLUEs across trials). Only p-values originally below 10^{-4} in any of the three GWAS methods (FarmCPU, GEMMA, ISIS EM-BLASSO) are shown. Both p-value sets are -log10 transformed. To the points is fitted a smooth spline constrained to be monotonously increasing. 4 p-value thresholds are shown as dashed lines, the yellow and red horizontal ones are the 0.01 and 0.05 thresholds estimated using simpleM, while the green and blue vertical ones are the 0.01 and 0.05 thresholds estimated using PermGWAS2.

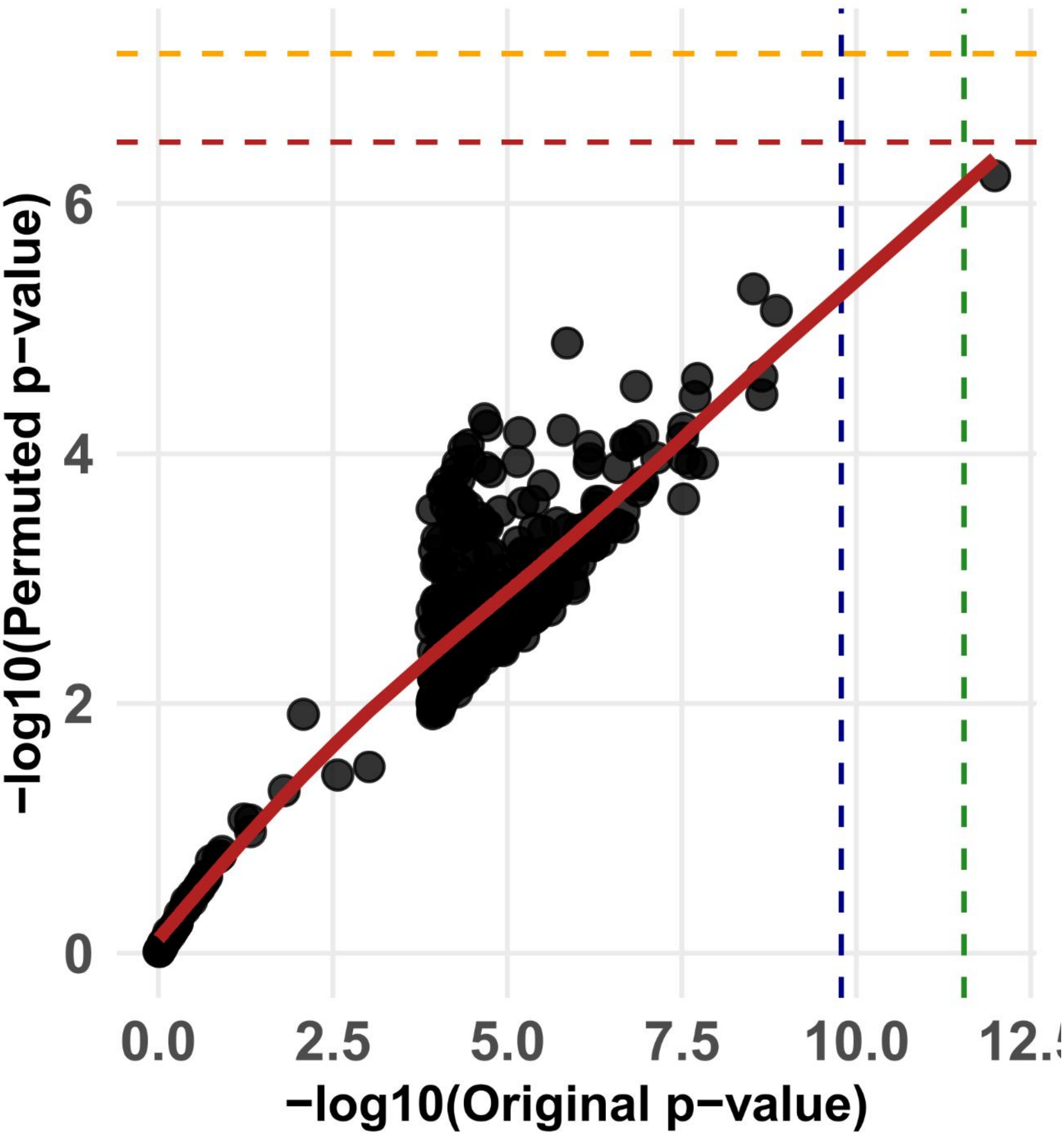

Extended data figure 34: Scatterplot of GEMMA parametric p-values (unpermuted) and permutation-derived p-values for Lodging in Nordic Seed 2019. Only p-values originally below 10^{-4} in any of the three GWAS methods (FarmCPU, GEMMA, ISIS EM-BLASSO) are shown. Both p-value sets are -log10 transformed. To the points is fitted a smooth spline constrained to be monotonously increasing. 4 p-value thresholds are shown as dashed lines, the yellow and red horizontal ones are the 0.01 and 0.05 thresholds estimated using simpleM, while the green and blue vertical ones are the 0.01 and 0.05 thresholds estimated using PermGWAS2.

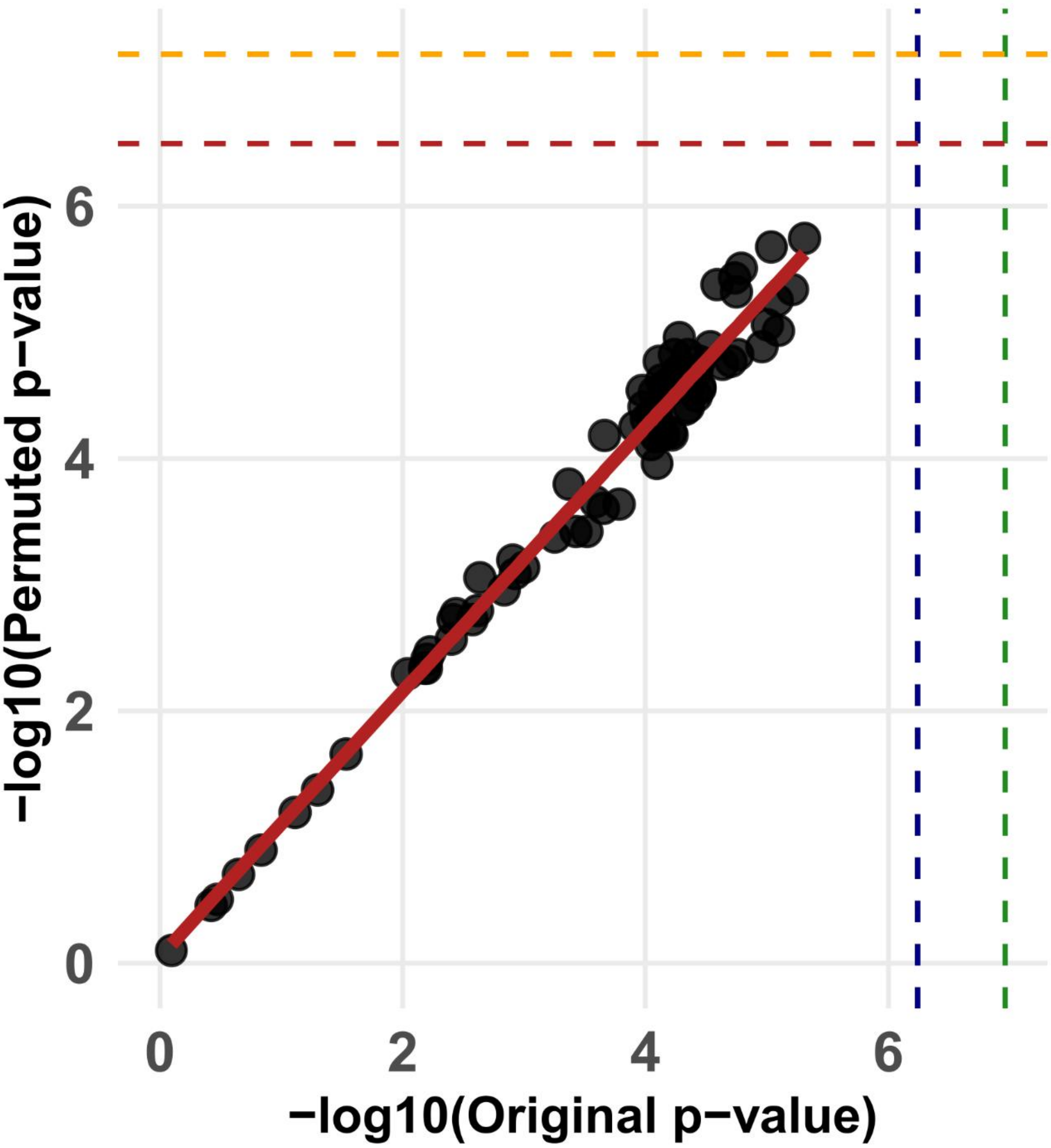

Extended data figure 35: Scatterplot of GEMMA parametric p-values (unpermuted) and permutation-derived p-values for Lodging in Nordic Seed 2020. Only p-values originally below 10^{-4} in any of the three GWAS methods (FarmCPU, GEMMA, ISIS EM-BLASSO) are shown. Both p-value sets are -log10 transformed. To the points is fitted a smooth spline constrained to be monotonously increasing. 4 p-value thresholds are shown as dashed lines, the yellow and red horizontal ones are the 0.01 and 0.05 thresholds estimated using simpleM, while the green and blue vertical ones are the 0.01 and 0.05 thresholds estimated using PermGWAS2.

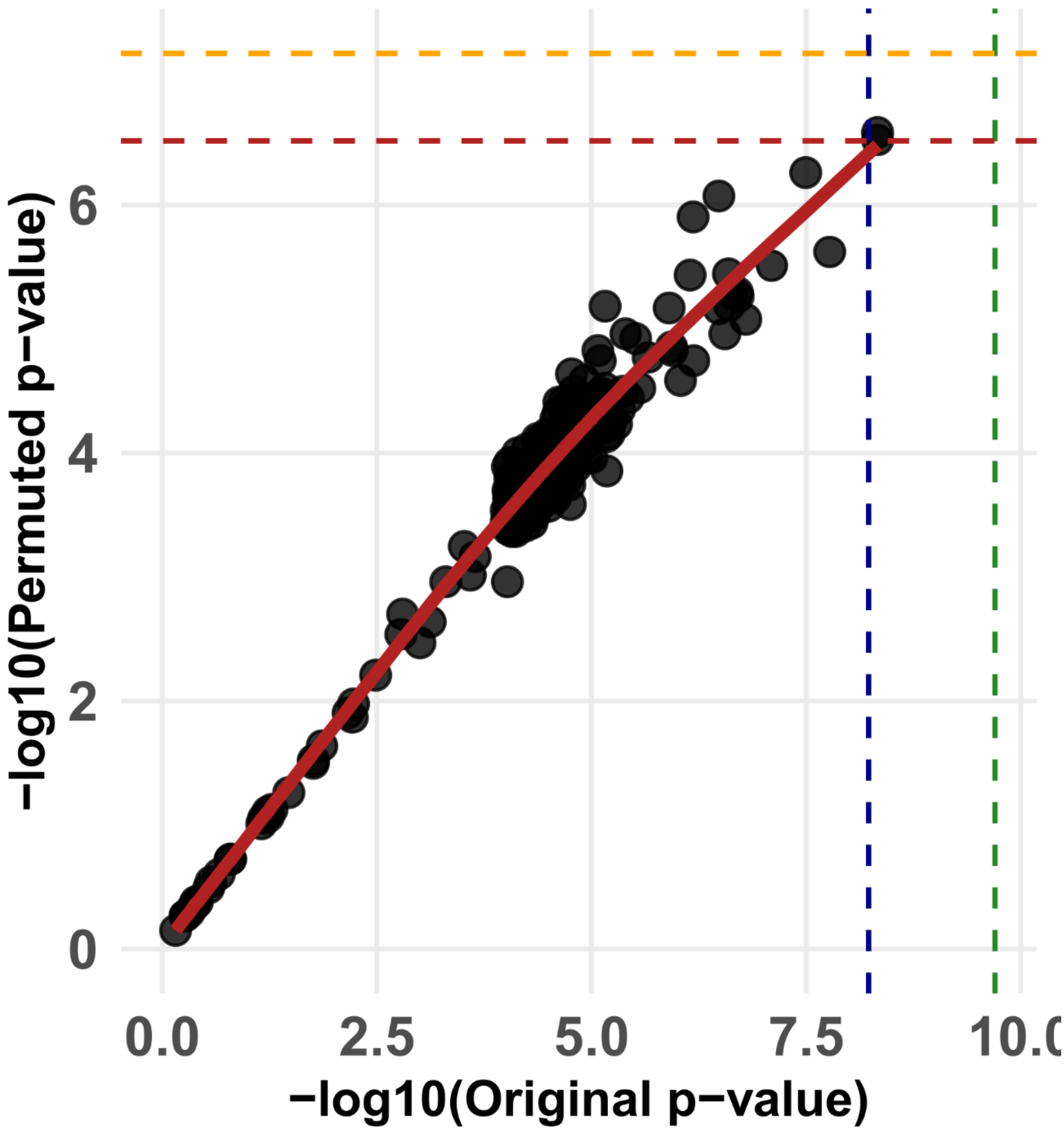

Extended data figure 36: Scatterplot of GEMMA parametric p-values (unpermuted) and permutation-derived p-values for Lodging in Sejet 2021. Only p-values originally below 10^{-4} in any of the three GWAS methods (FarmCPU, GEMMA, ISIS EM-BLASSO) are shown. Both p-value sets are -log10 transformed. To the points is fitted a smooth spline constrained to be monotonously increasing. 4 p-value thresholds are shown as dashed lines, the yellow and red horizontal ones are the 0.01 and 0.05 thresholds estimated using simpleM, while the green and blue vertical ones are the 0.01 and 0.05 thresholds estimated using PermGWAS2.

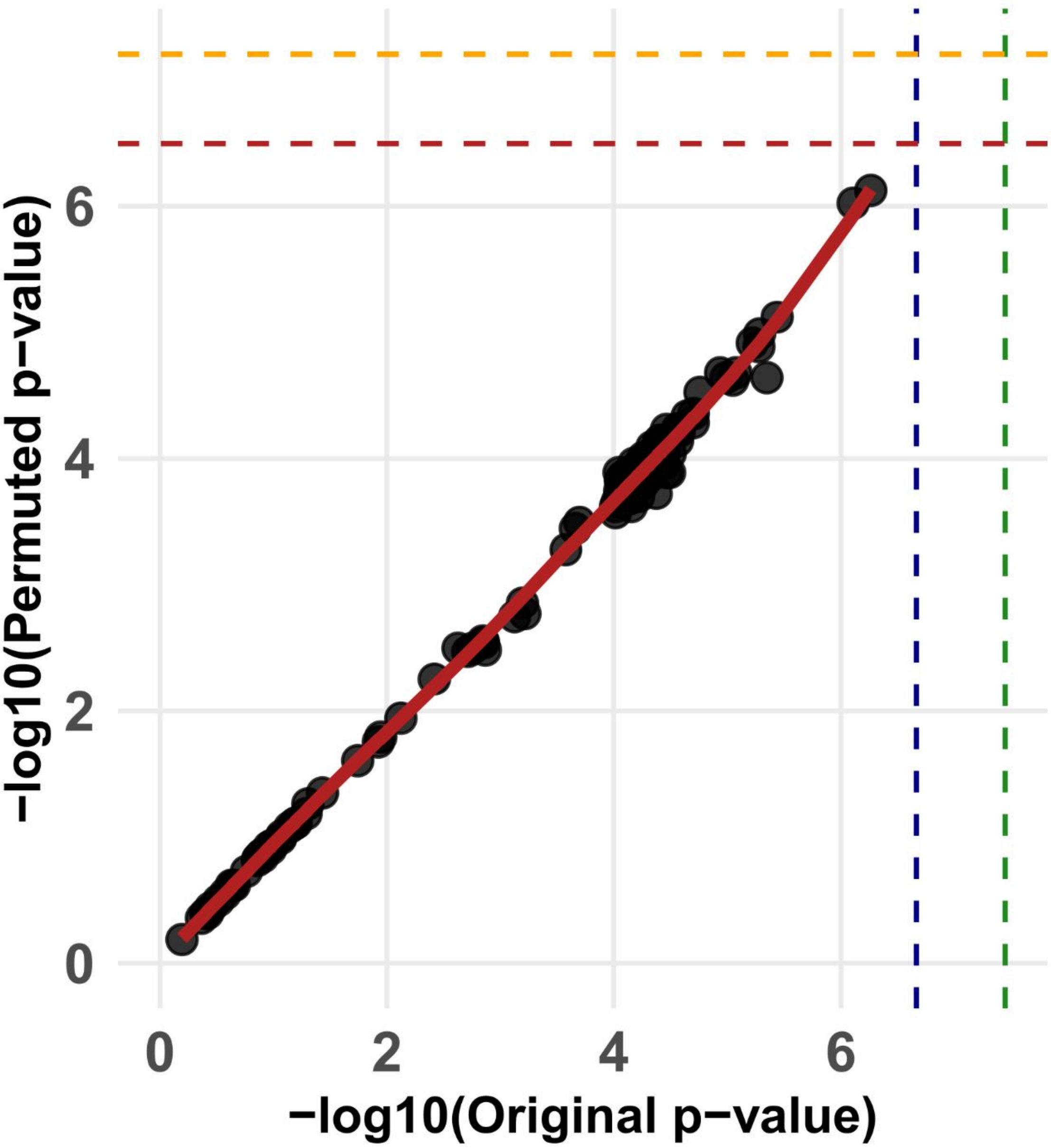

Extended data figure 37: Scatterplot of GEMMA parametric p-values (unpermuted) and permutation-derived p-values for Maturation Date (BLUEs across trials). Only p-values originally below 10^{-4} in any of the three GWAS methods (FarmCPU, GEMMA, ISIS EM-BLASSO) are shown. Both p-value sets are -log10 transformed. To the points is fitted a smooth spline constrained to be monotonously increasing. 4 p-value thresholds are shown as dashed lines, the yellow and red horizontal ones are the 0.01 and 0.05 thresholds estimated using simpleM, while the green and blue vertical ones are the 0.01 and 0.05 thresholds estimated using PermGWAS2.

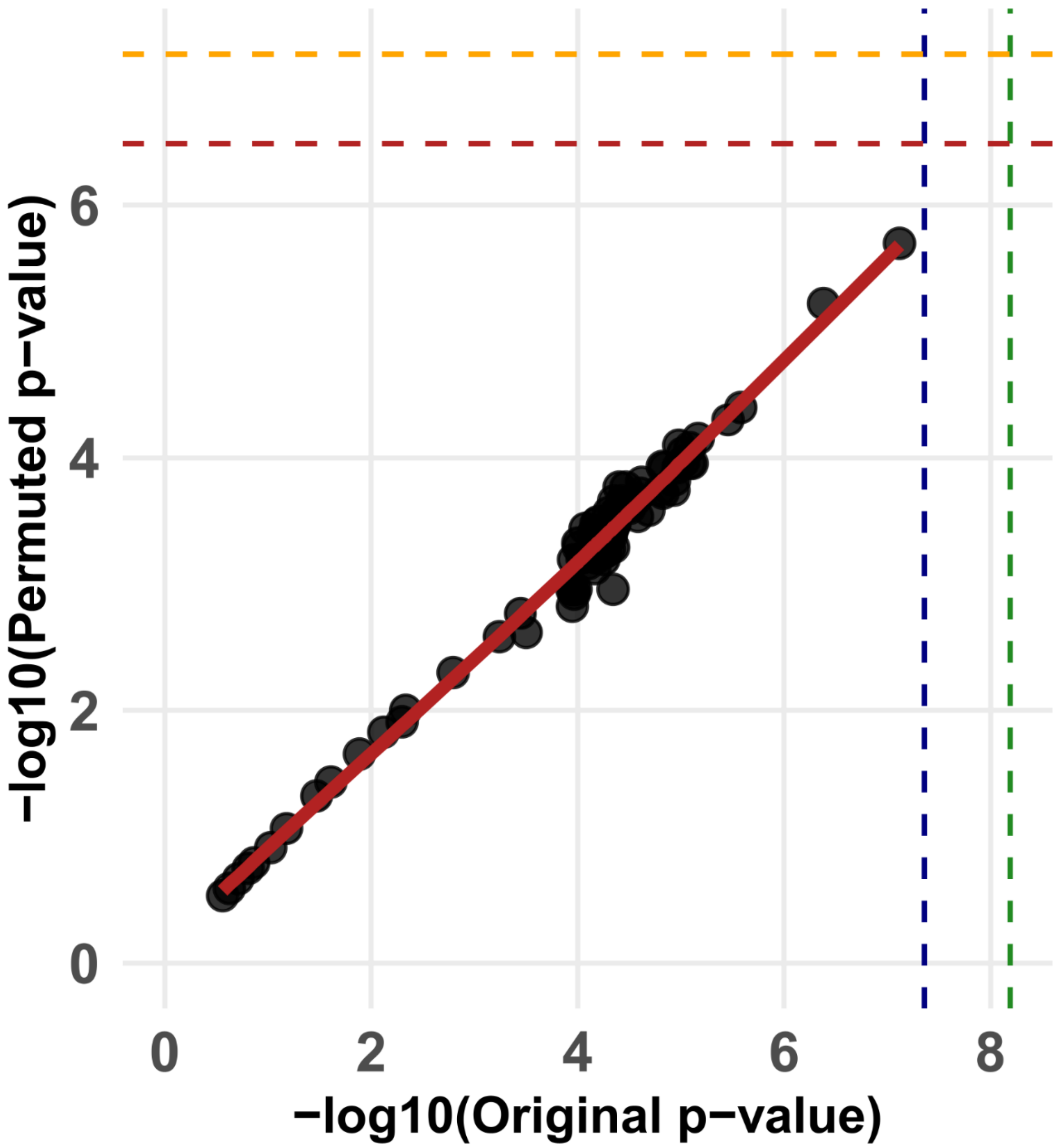

Extended data figure 38: Scatterplot of GEMMA parametric p-values (unpermuted) and permutation-derived p-values for Maturation Date in Nordic Seed 2018. Only p-values originally below 10^{-4} in any of the three GWAS methods (FarmCPU, GEMMA, ISIS EM-BLASSO) are shown. Both p-value sets are -log10 transformed. To the points is fitted a smooth spline constrained to be monotonously increasing. 4 p-value thresholds are shown as dashed lines, the yellow and red horizontal ones are the 0.01 and 0.05 thresholds estimated using simpleM, while the green and blue vertical ones are the 0.01 and 0.05 thresholds estimated using PermGWAS2.

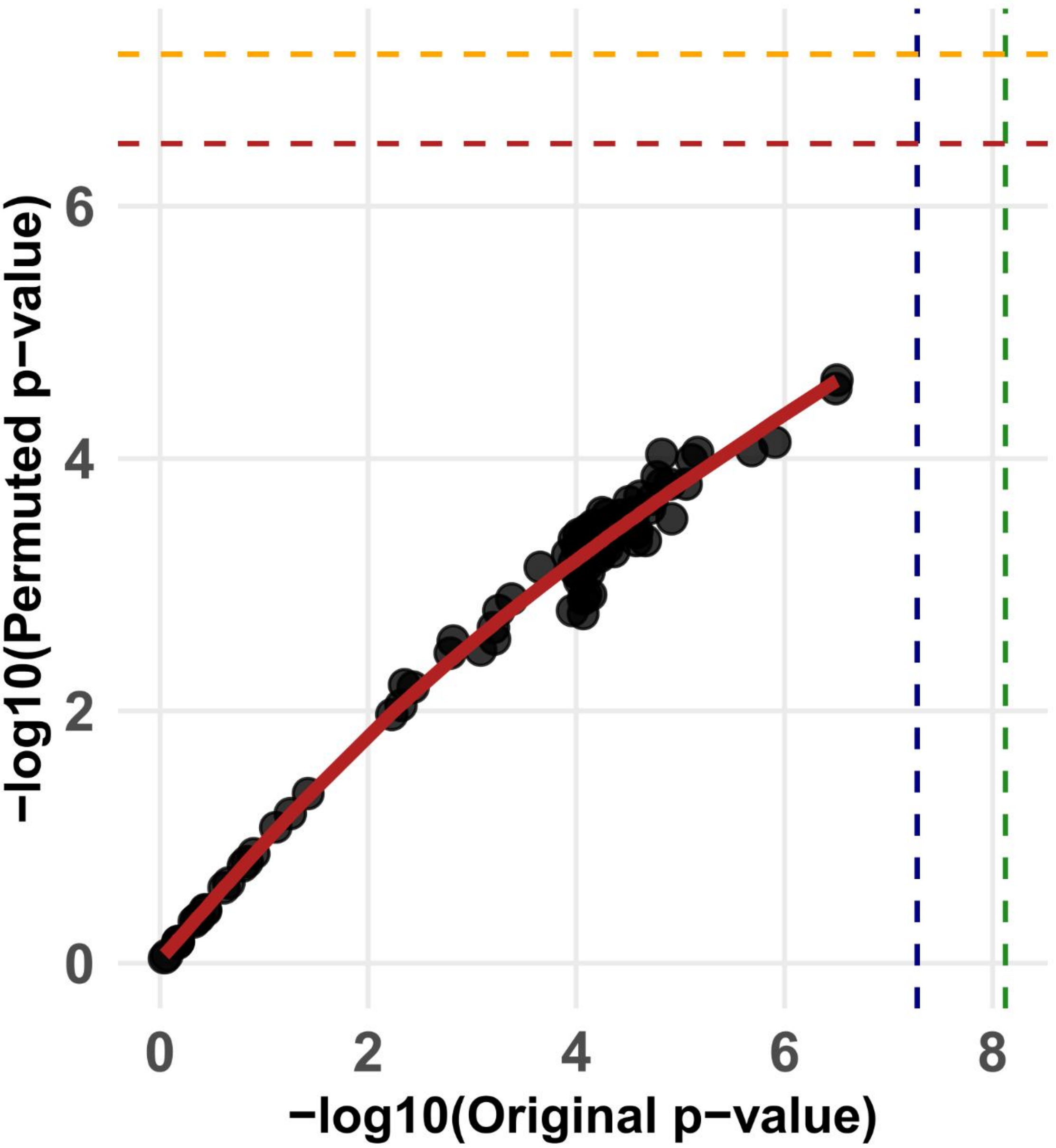

Extended data figure 39: Scatterplot of GEMMA parametric p-values (unpermuted) and permutation-derived p-values for Maturation Date in Nordic Seed 2019. Only p-values originally below 10^{-4} in any of the three GWAS methods (FarmCPU, GEMMA, ISIS EM-BLASSO) are shown. Both p-value sets are -log10 transformed. To the points is fitted a smooth spline constrained to be monotonously increasing. 4 p-value thresholds are shown as dashed lines, the yellow and red horizontal ones are the 0.01 and 0.05 thresholds estimated using simpleM, while the green and blue vertical ones are the 0.01 and 0.05 thresholds estimated using PermGWAS2.

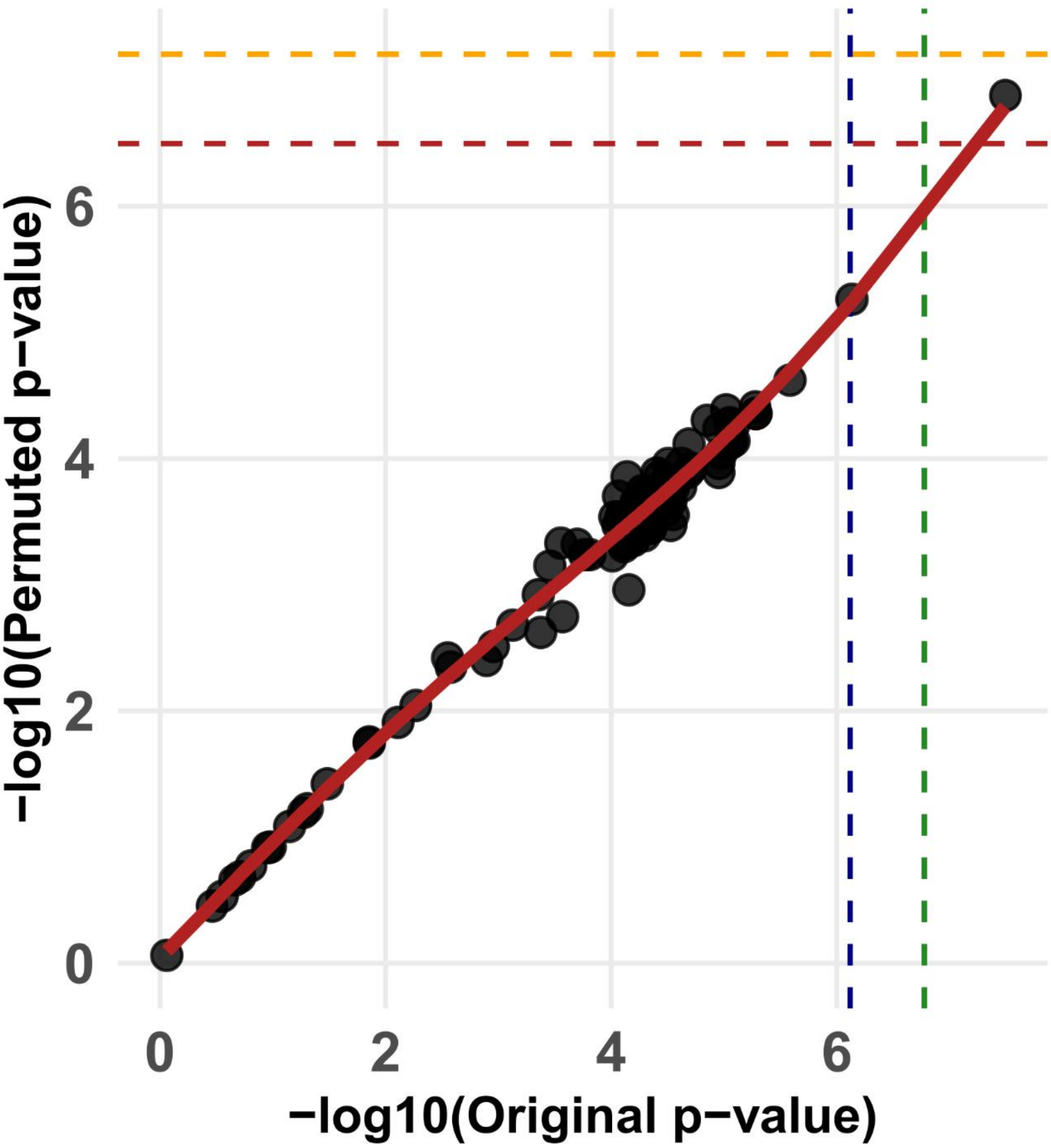

Extended data figure 40: Scatterplot of GEMMA parametric p-values (unpermuted) and permutation-derived p-values for Maturation Date in Nordic Seed 2020. Only p-values originally below 10^{-4} in any of the three GWAS methods (FarmCPU, GEMMA, ISIS EM-BLASSO) are shown. Both p-value sets are -log10 transformed. To the points is fitted a smooth spline constrained to be monotonously increasing. 4 p-value thresholds are shown as dashed lines, the yellow and red horizontal ones are the 0.01 and 0.05 thresholds estimated using simpleM, while the green and blue vertical ones are the 0.01 and 0.05 thresholds estimated using PermGWAS2.

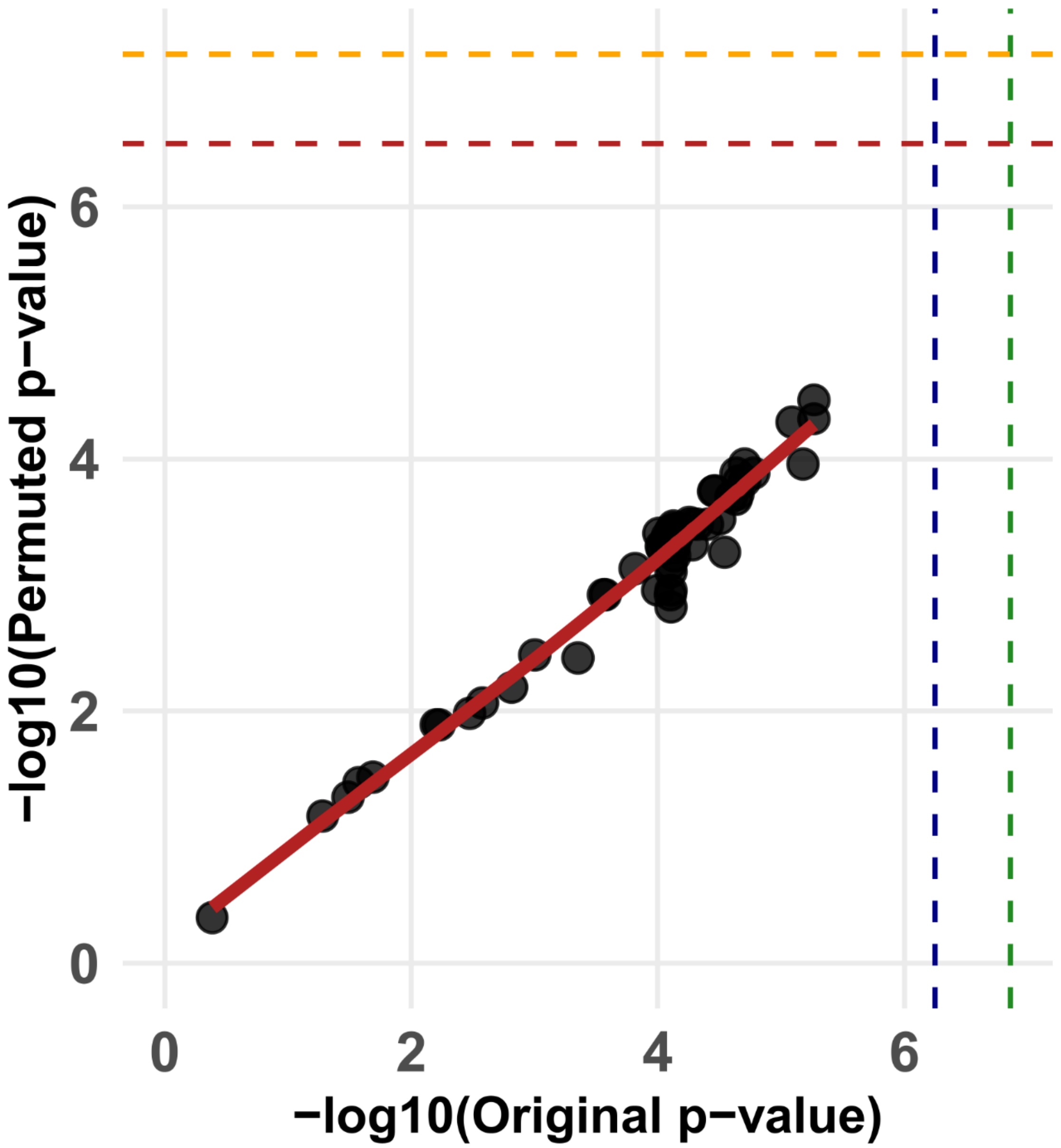

Extended data figure 41: Scatterplot of GEMMA parametric p-values (unpermuted) and permutation-derived p-values for Maturation Date in Sejet 2019. Only p-values originally below 10^{-4} in any of the three GWAS methods (FarmCPU, GEMMA, ISIS EM-BLASSO) are shown. Both p-value sets are -log10 transformed. To the points is fitted a smooth spline constrained to be monotonously increasing. 4 p-value thresholds are shown as dashed lines, the yellow and red horizontal ones are the 0.01 and 0.05 thresholds estimated using simpleM, while the green and blue vertical ones are the 0.01 and 0.05 thresholds estimated using PermGWAS2.

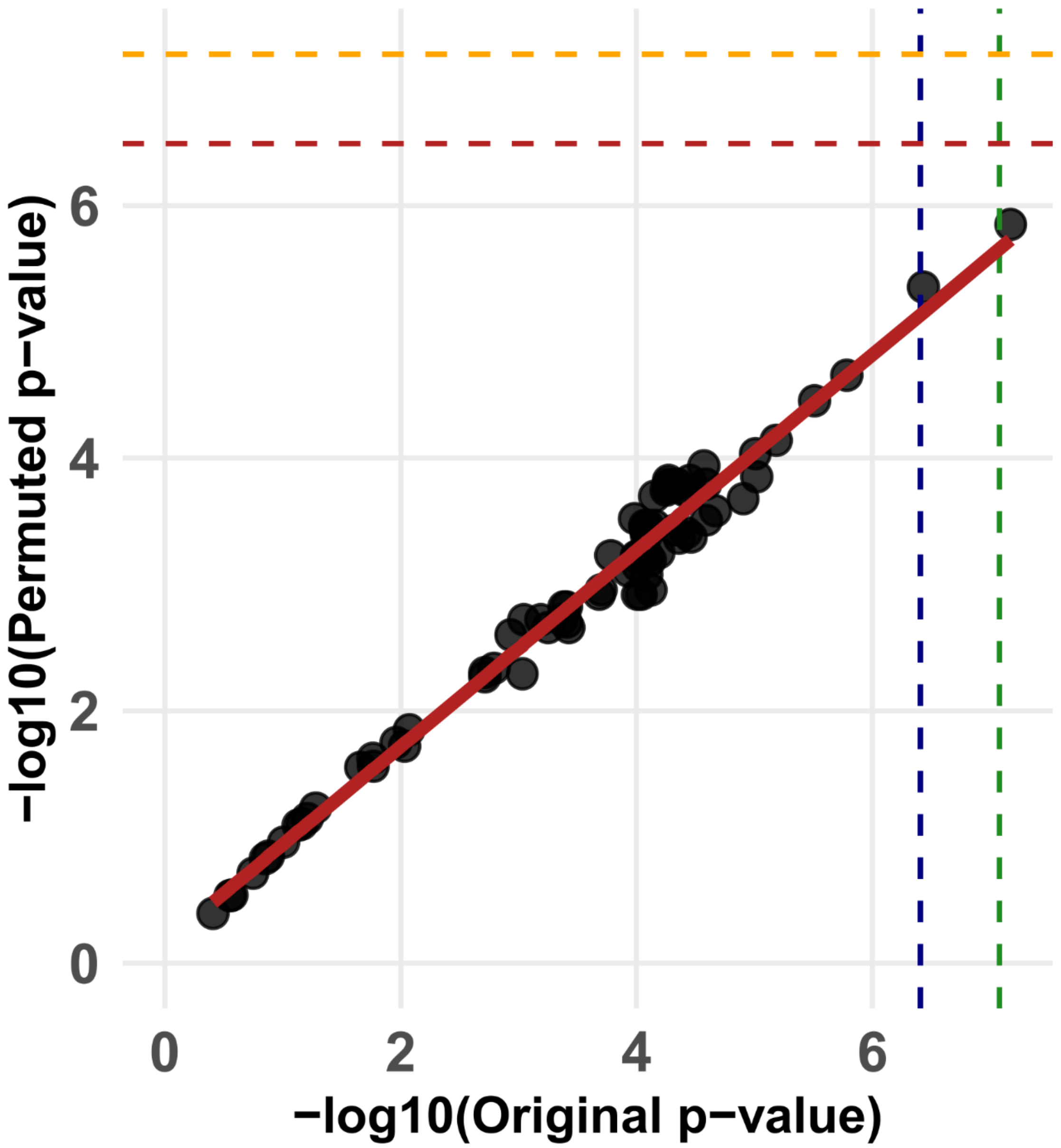

Extended data figure 42: Scatterplot of GEMMA parametric p-values (unpermuted) and permutation-derived p-values for Number of Ovules (BLUEs across trials). Only p-values originally below 10^{-4} in any of the three GWAS methods (FarmCPU, GEMMA, ISIS EM-BLASSO) are shown. Both p-value sets are -log10 transformed. To the points is fitted a smooth spline constrained to be monotonously increasing. 4 p-value thresholds are shown as dashed lines, the yellow and red horizontal ones are the 0.01 and 0.05 thresholds estimated using simpleM, while the green and blue vertical ones are the 0.01 and 0.05 thresholds estimated using PermGWAS2.

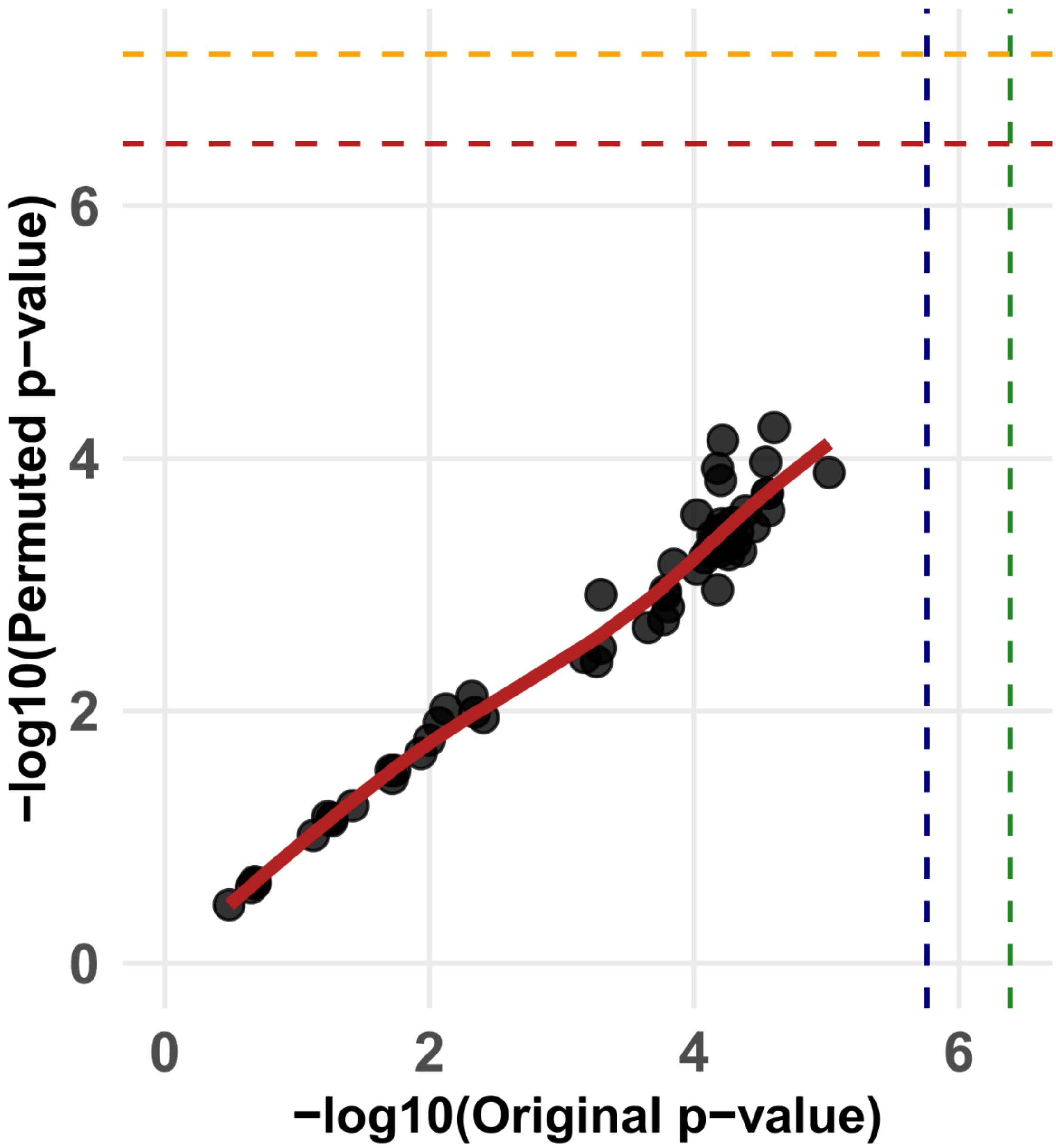

Extended data figure 43: Scatterplot of GEMMA parametric p-values (unpermuted) and permutation-derived p-values for Number of Ovules in Nordic Seed 2019. Only p-values originally below 10^{-4} in any of the three GWAS methods (FarmCPU, GEMMA, ISIS EM-BLASSO) are shown. Both p-value sets are -log10 transformed. To the points is fitted a smooth spline constrained to be monotonously increasing. 4 p-value thresholds are shown as dashed lines, the yellow and red horizontal ones are the 0.01 and 0.05 thresholds estimated using simpleM, while the green and blue vertical ones are the 0.01 and 0.05 thresholds estimated using PermGWAS2.

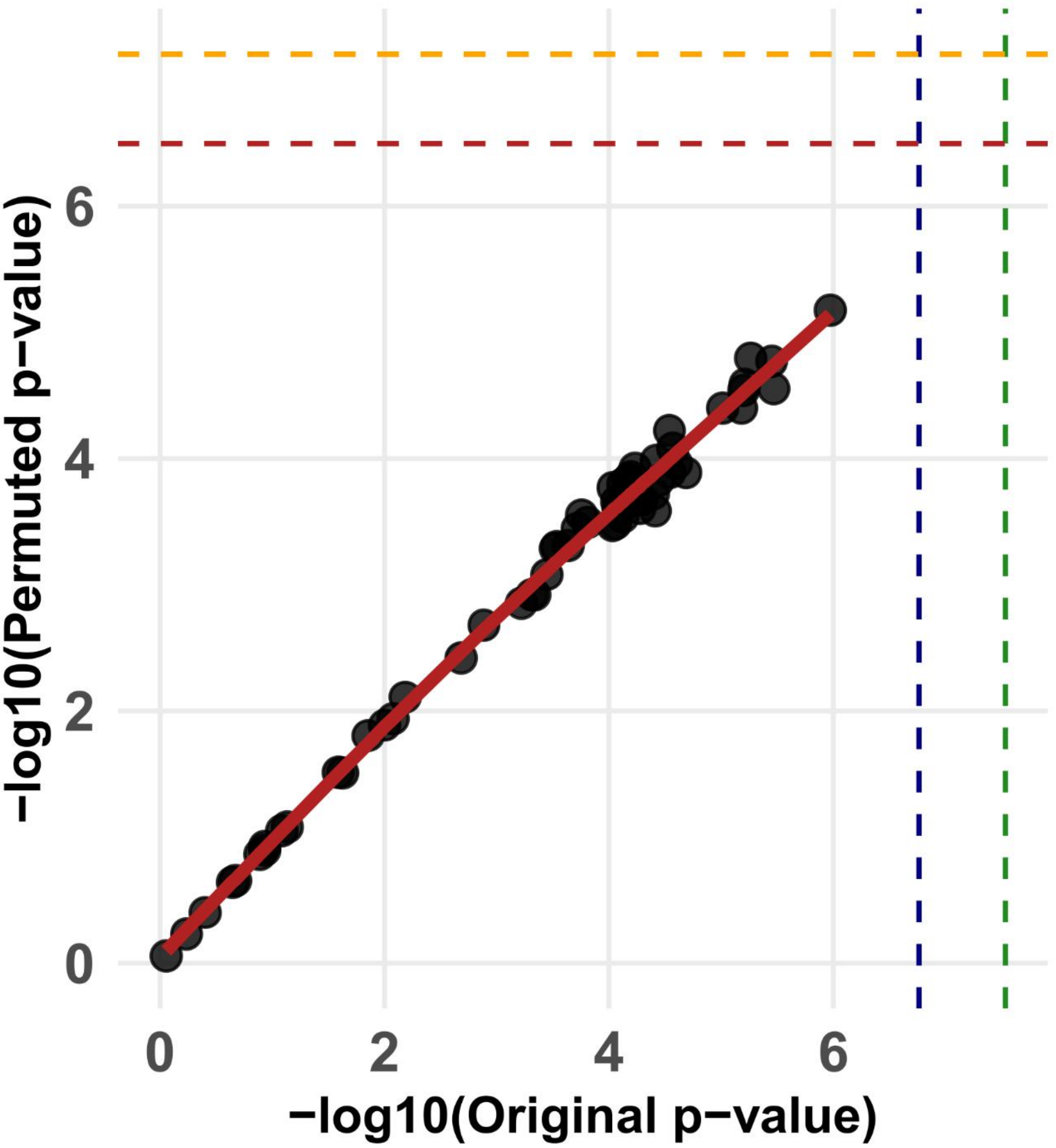

Extended data figure 44: Scatterplot of GEMMA parametric p-values (unpermuted) and permutation-derived p-values for Number of Ovules in Nordic Seed 2020. Only p-values originally below 10^{-4} in any of the three GWAS methods (FarmCPU, GEMMA, ISIS EM-BLASSO) are shown. Both p-value sets are -log10 transformed. To the points is fitted a smooth spline constrained to be monotonously increasing. 4 p-value thresholds are shown as dashed lines, the yellow and red horizontal ones are the 0.01 and 0.05 thresholds estimated using simpleM, while the green and blue vertical ones are the 0.01 and 0.05 thresholds estimated using PermGWAS2.

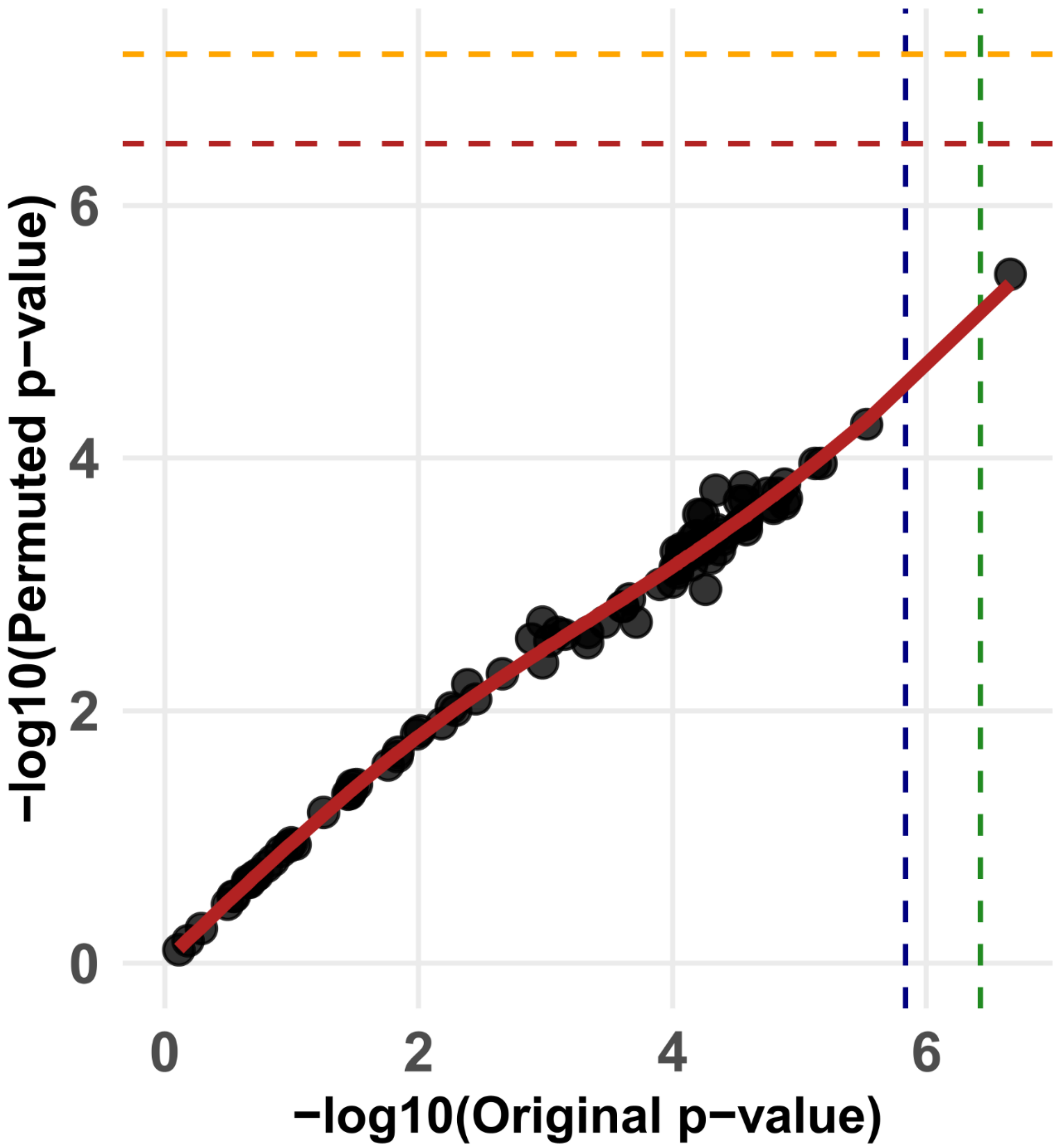

Extended data figure 45: Scatterplot of GEMMA parametric p-values (unpermuted) and permutation-derived p-values for Plant Height (BLUEs across trials). Only p-values originally below 10^{-4} in any of the three GWAS methods (FarmCPU, GEMMA, ISIS EM-BLASSO) are shown. Both p-value sets are -log10 transformed. To the points is fitted a smooth spline constrained to be monotonously increasing. 4 p-value thresholds are shown as dashed lines, the yellow and red horizontal ones are the 0.01 and 0.05 thresholds estimated using simpleM, while the green and blue vertical ones are the 0.01 and 0.05 thresholds estimated using PermGWAS2.

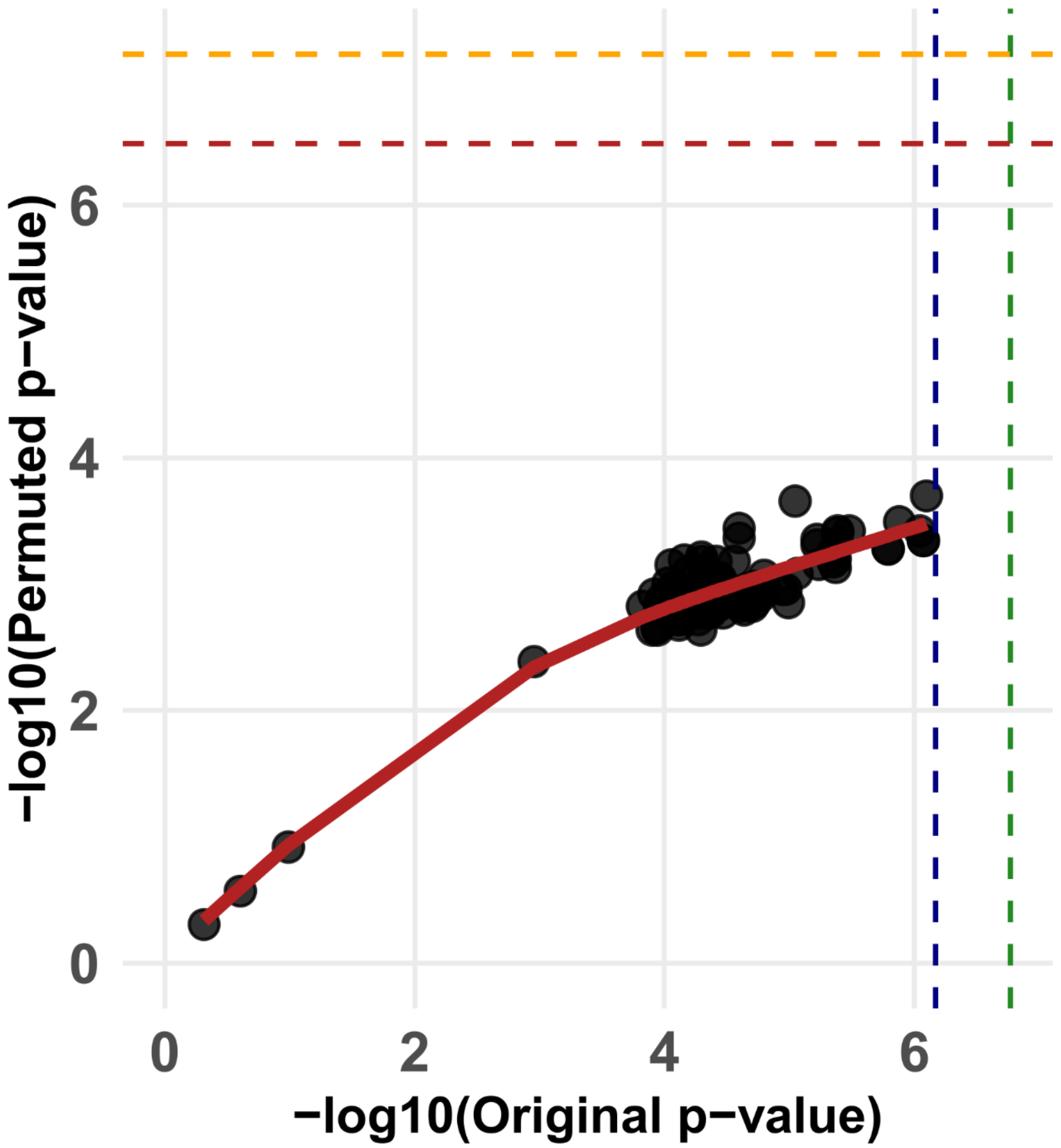

Extended data figure 46: Scatterplot of GEMMA parametric p-values (unpermuted) and permutation-derived p-values for Plant Height in Nordic Seed 2018. Only p-values originally below 10^{-4} in any of the three GWAS methods (FarmCPU, GEMMA, ISIS EM-BLASSO) are shown. Both p-value sets are -log10 transformed. To the points is fitted a smooth spline constrained to be monotonously increasing. 4 p-value thresholds are shown as dashed lines, the yellow and red horizontal ones are the 0.01 and 0.05 thresholds estimated using simpleM, while the green and blue vertical ones are the 0.01 and 0.05 thresholds estimated using PermGWAS2.

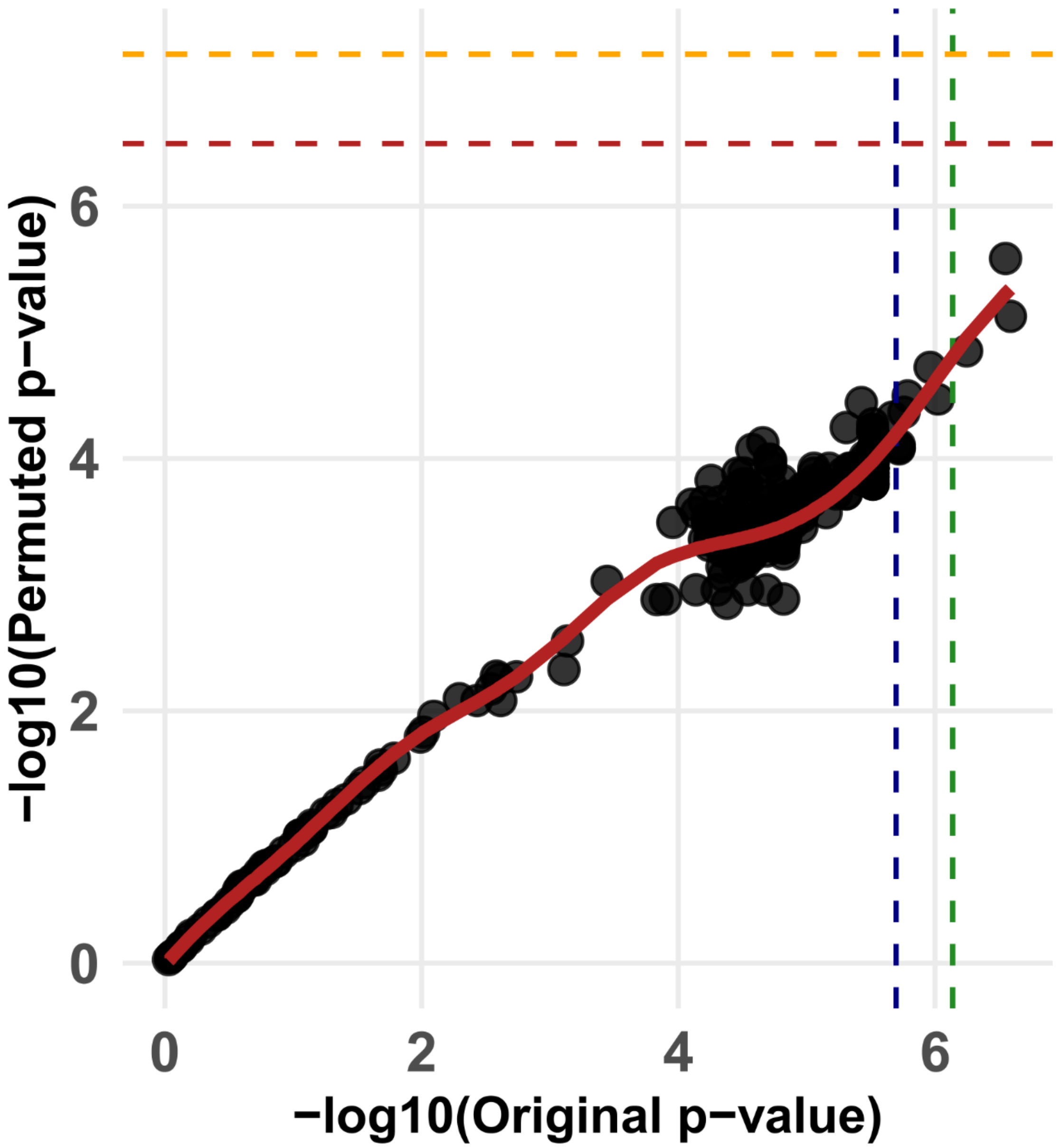

Extended data figure 47: Scatterplot of GEMMA parametric p-values (unpermuted) and permutation-derived p-values for Plant Height in Nordic Seed 2019. Only p-values originally below 10^{-4} in any of the three GWAS methods (FarmCPU, GEMMA, ISIS EM-BLASSO) are shown. Both p-value sets are -log10 transformed. To the points is fitted a smooth spline constrained to be monotonously increasing. 4 p-value thresholds are shown as dashed lines, the yellow and red horizontal ones are the 0.01 and 0.05 thresholds estimated using simpleM, while the green and blue vertical ones are the 0.01 and 0.05 thresholds estimated using PermGWAS2.

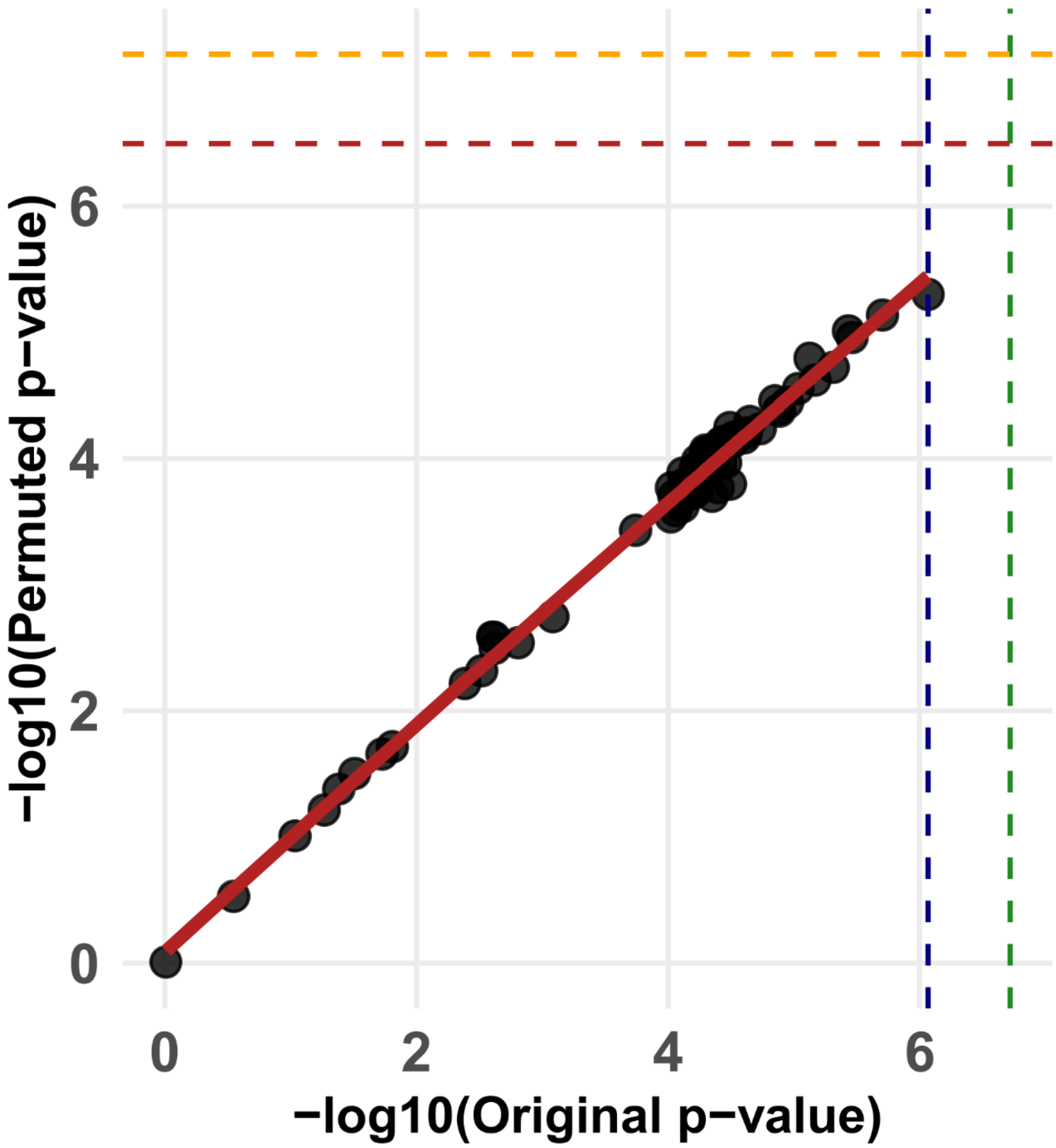

Extended data figure 48: Scatterplot of GEMMA parametric p-values (unpermuted) and permutation-derived p-values for Plant Height in Nordic Seed 2020. Only p-values originally below 10^{-4} in any of the three GWAS methods (FarmCPU, GEMMA, ISIS EM-BLASSO) are shown. Both p-value sets are -log10 transformed. To the points is fitted a smooth spline constrained to be monotonously increasing. 4 p-value thresholds are shown as dashed lines, the yellow and red horizontal ones are the 0.01 and 0.05 thresholds estimated using simpleM, while the green and blue vertical ones are the 0.01 and 0.05 thresholds estimated using PermGWAS2.

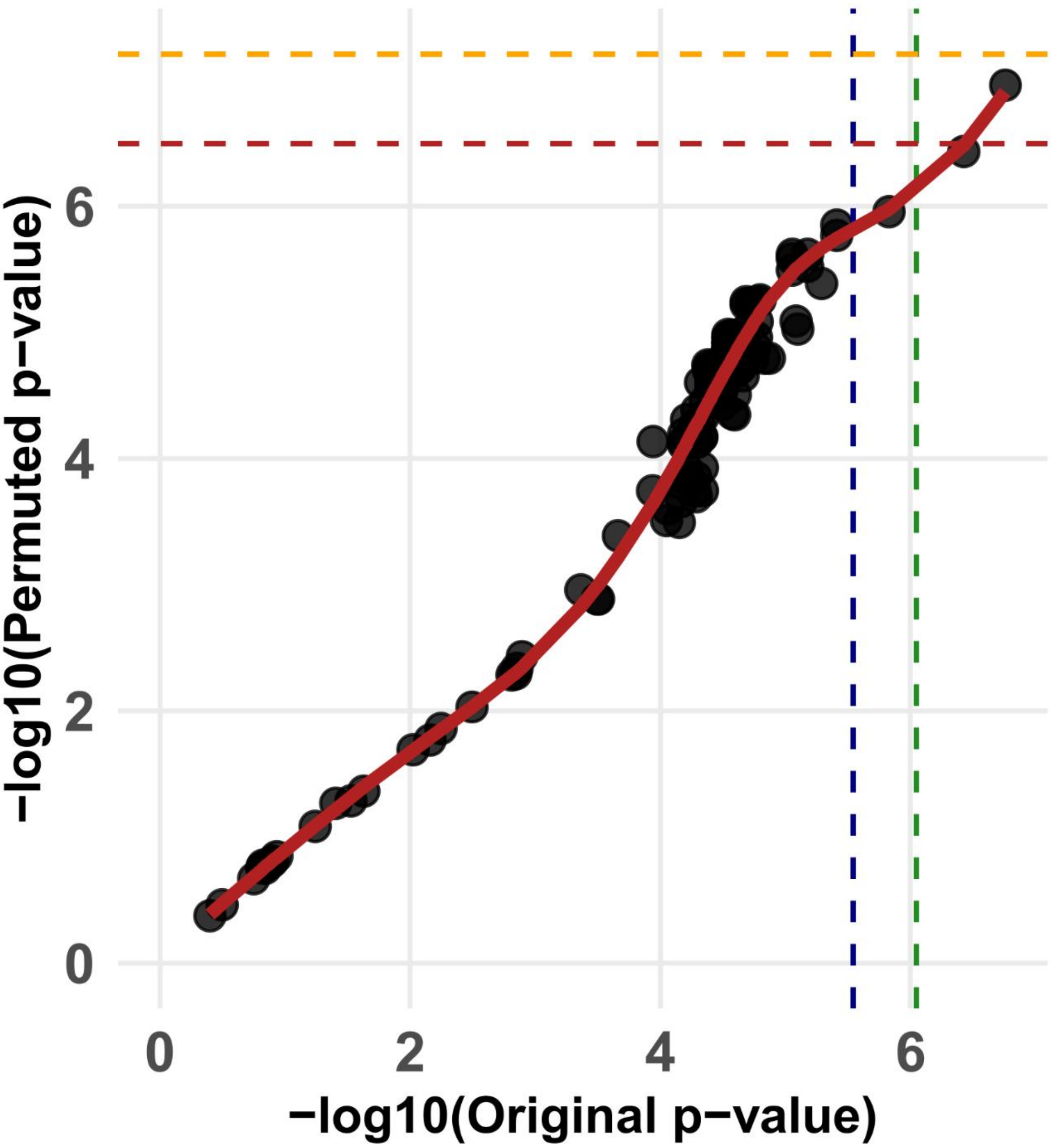

Extended data figure 49: Scatterplot of GEMMA parametric p-values (unpermuted) and permutation-derived p-values for Plant Height in Sejet 2019. Only p-values originally below 10^{-4} in any of the three GWAS methods (FarmCPU, GEMMA, ISIS EM-BLASSO) are shown. Both p-value sets are -log10 transformed. To the points is fitted a smooth spline constrained to be monotonously increasing. 4 p-value thresholds are shown as dashed lines, the yellow and red horizontal ones are the 0.01 and 0.05 thresholds estimated using simpleM, while the green and blue vertical ones are the 0.01 and 0.05 thresholds estimated using PermGWAS2.

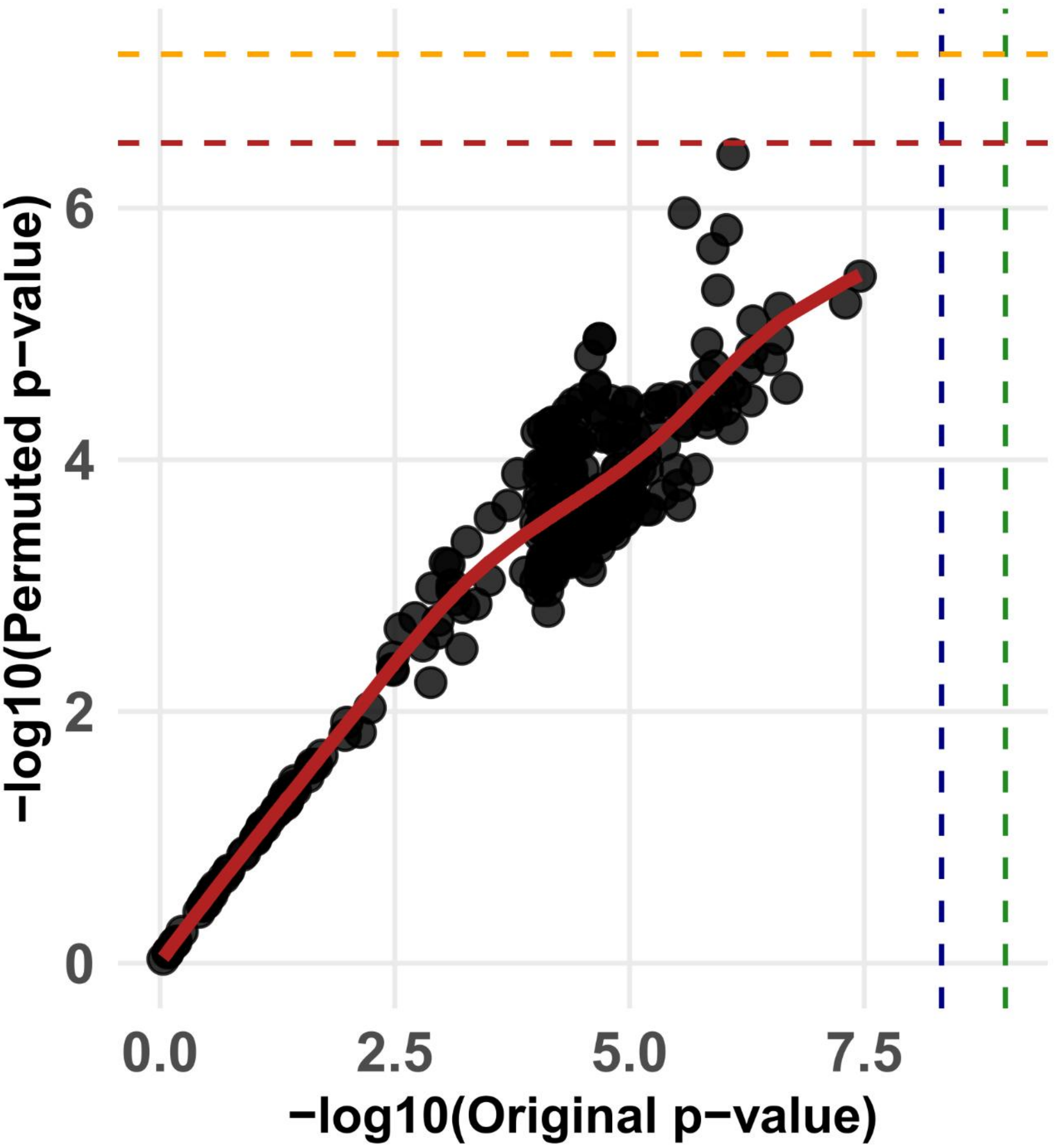

Extended data figure 50: Scatterplot of GEMMA parametric p-values (unpermuted) and permutation-derived p-values for Plant Height in Sejet 2021. Only p-values originally below 10^{-4} in any of the three GWAS methods (FarmCPU, GEMMA, ISIS EM-BLASSO) are shown. Both p-value sets are -log10 transformed. To the points is fitted a smooth spline constrained to be monotonously increasing. 4 p-value thresholds are shown as dashed lines, the yellow and red horizontal ones are the 0.01 and 0.05 thresholds estimated using simpleM, while the green and blue vertical ones are the 0.01 and 0.05 thresholds estimated using PermGWAS2.

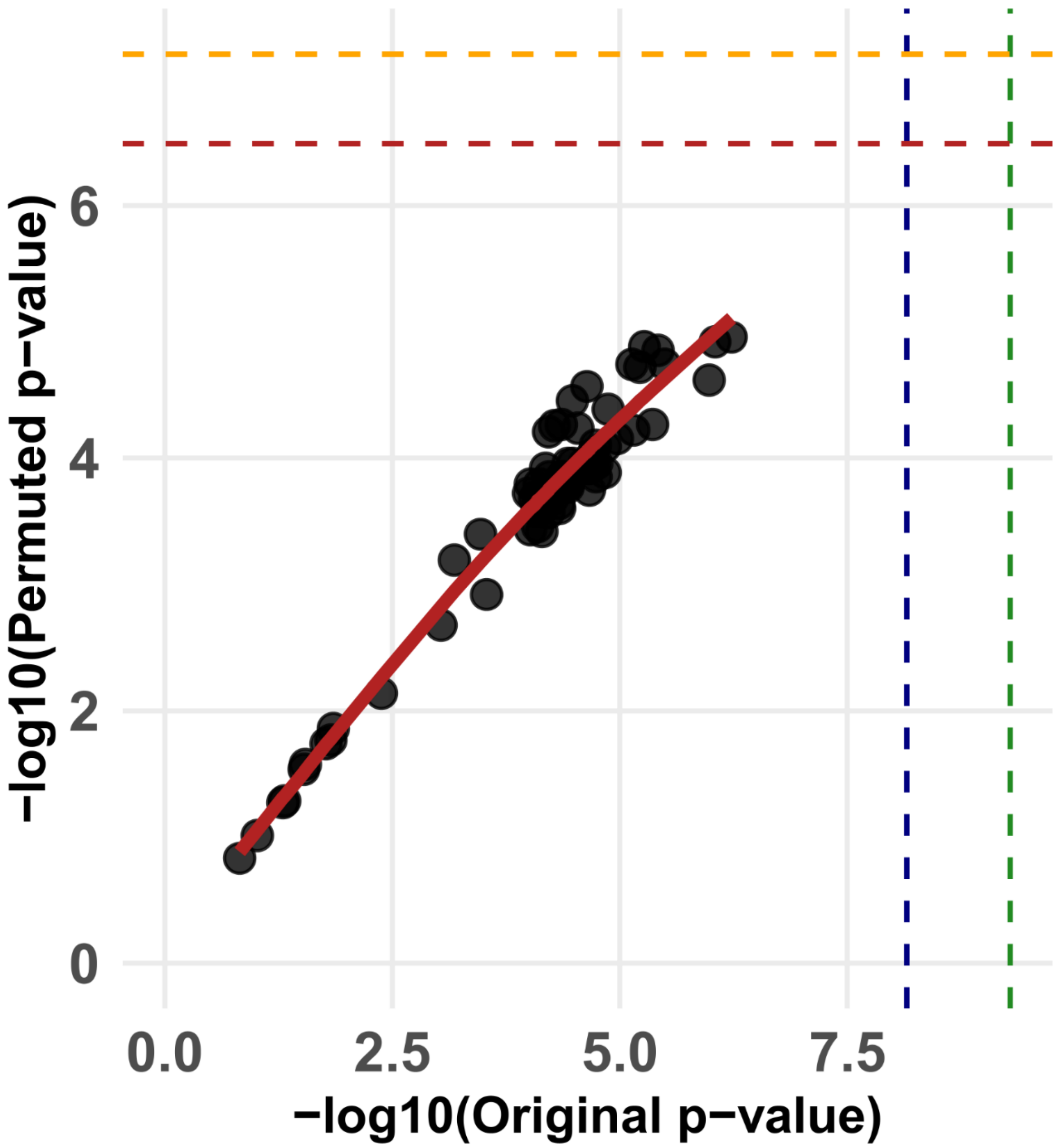

Extended data figure 51: Scatterplot of GEMMA parametric p-values (unpermuted) and permutation-derived p-values for Seed Circularity type 1 (BLUEs across trials). Only p-values originally below 10^{-4} in any of the three GWAS methods (FarmCPU, GEMMA, ISIS EM-BLASSO) are shown. Both p-value sets are -log10 transformed. To the points is fitted a smooth spline constrained to be monotonously increasing. 4 p-value thresholds are shown as dashed lines, the yellow and red horizontal ones are the 0.01 and 0.05 thresholds estimated using simpleM, while the green and blue vertical ones are the 0.01 and 0.05 thresholds estimated using PermGWAS2.

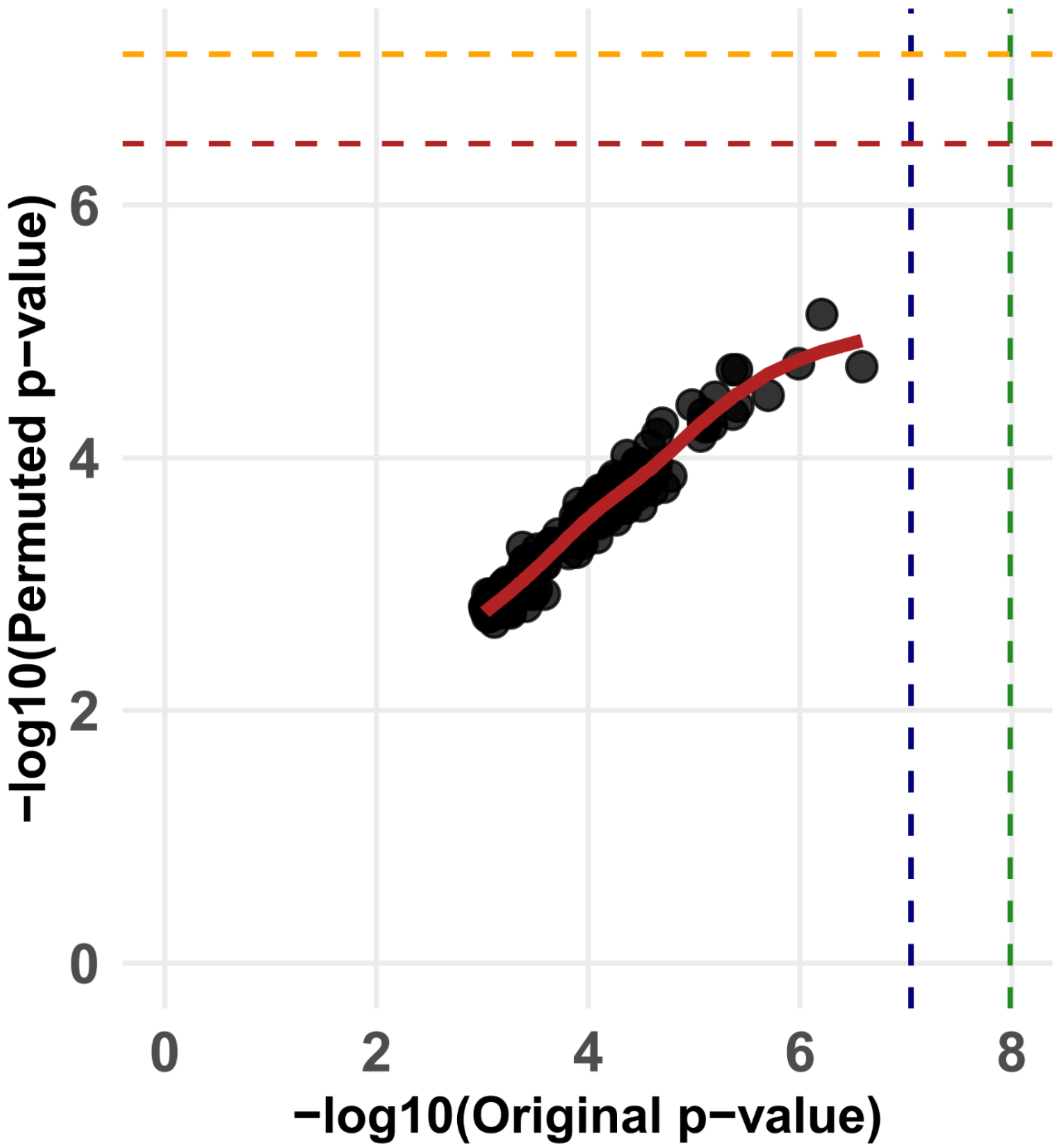

Extended data figure 52: Scatterplot of GEMMA parametric p-values (unpermuted) and permutation-derived p-values for Seed Circularity type 1 in Nordic Seed 2018. Only p-values originally below 10^{-4} in any of the three GWAS methods (FarmCPU, GEMMA, ISIS EM-BLASSO) are shown. Both p-value sets are -log10 transformed. To the points is fitted a smooth spline constrained to be monotonously increasing. 4 p-value thresholds are shown as dashed lines, the yellow and red horizontal ones are the 0.01 and 0.05 thresholds estimated using simpleM, while the green and blue vertical ones are the 0.01 and 0.05 thresholds estimated using PermGWAS2.

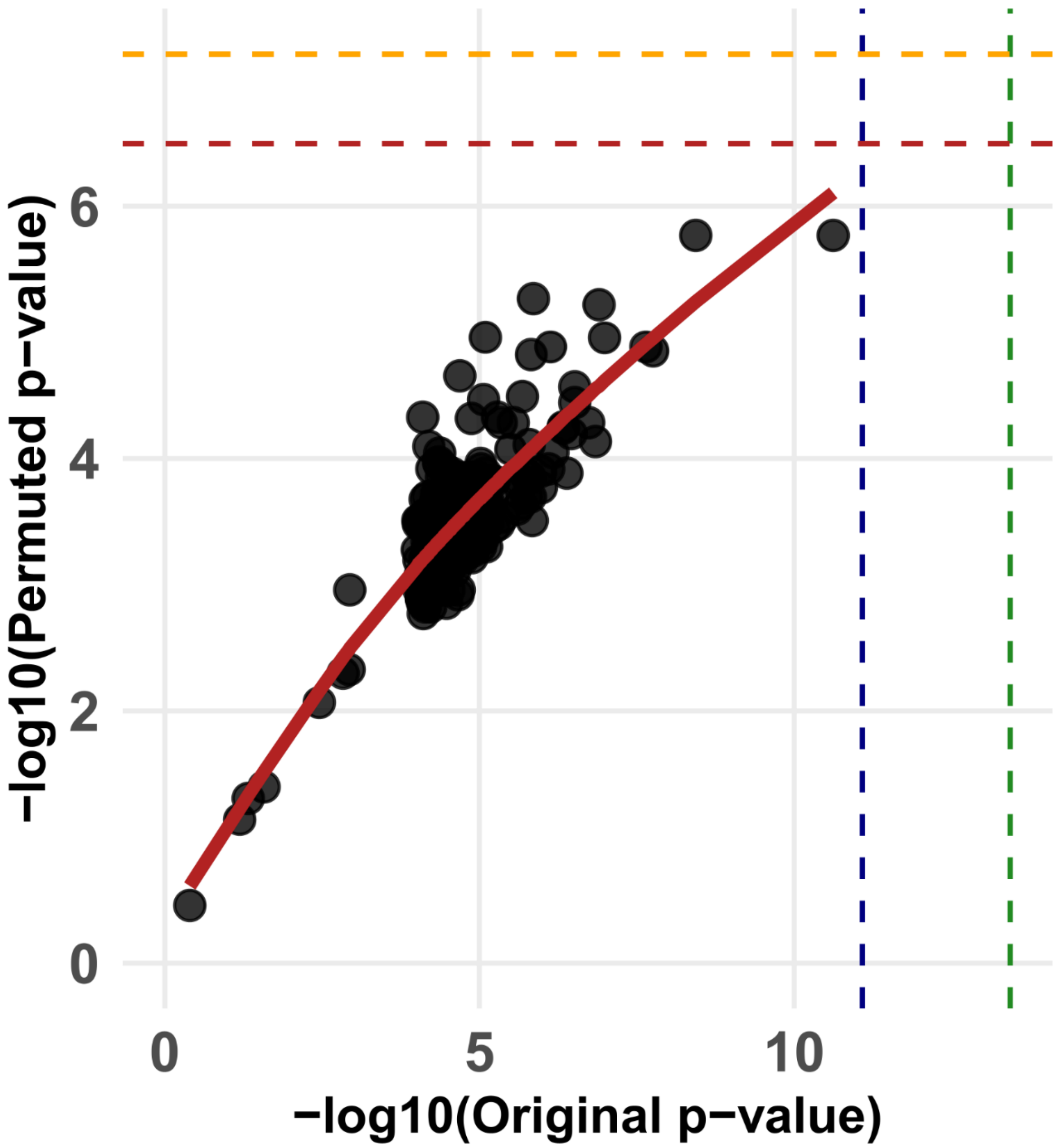

Extended data figure 53: Scatterplot of GEMMA parametric p-values (unpermuted) and permutation-derived p-values for Seed Circularity type 1 in Nordic Seed 2020. Only p-values originally below 10^{-4} in any of the three GWAS methods (FarmCPU, GEMMA, ISIS EM-BLASSO) are shown. Both p-value sets are -log10 transformed. To the points is fitted a smooth spline constrained to be monotonously increasing. 4 p-value thresholds are shown as dashed lines, the yellow and red horizontal ones are the 0.01 and 0.05 thresholds estimated using simpleM, while the green and blue vertical ones are the 0.01 and 0.05 thresholds estimated using PermGWAS2.

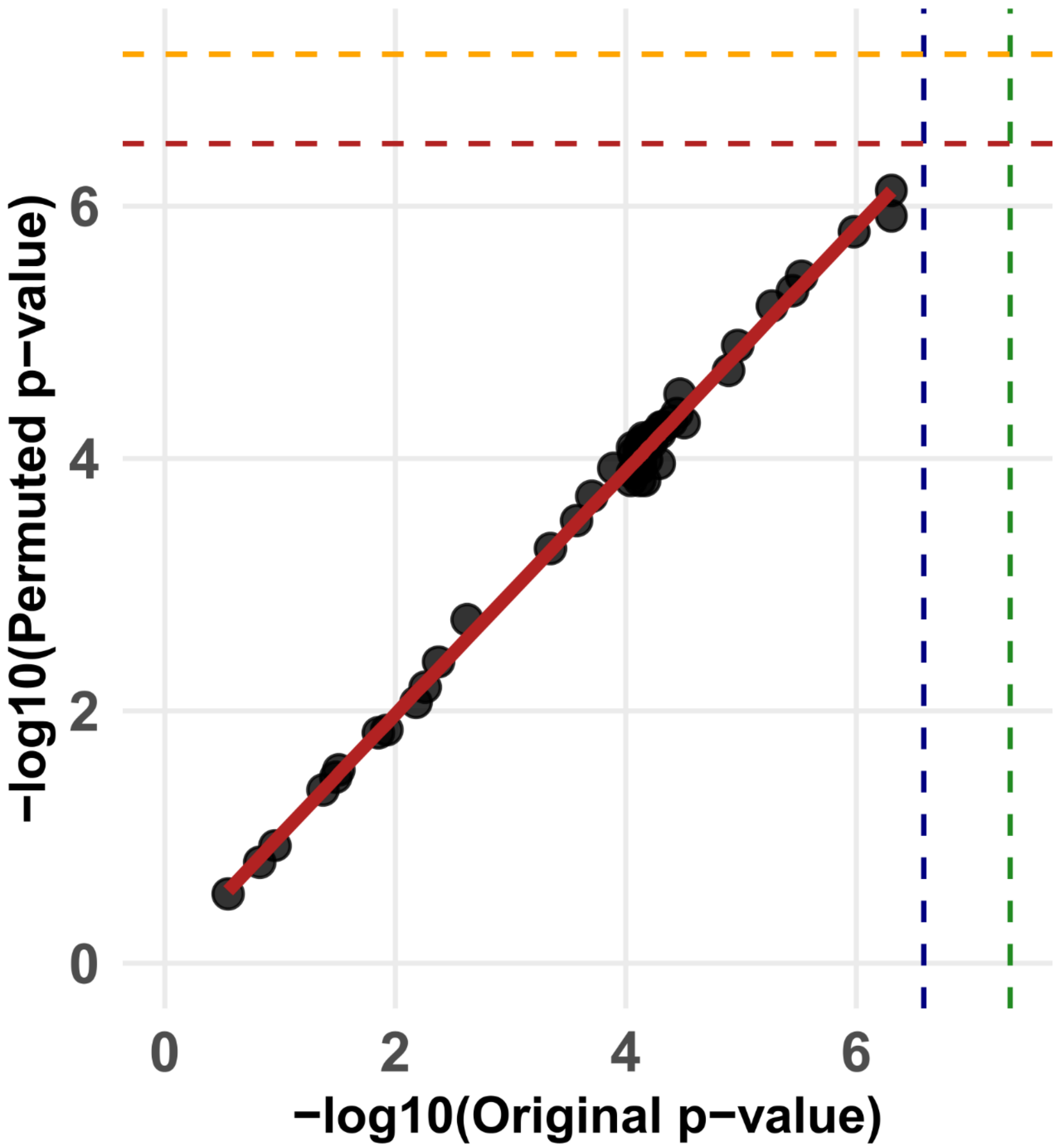

Extended data figure 54: Scatterplot of GEMMA parametric p-values (unpermuted) and permutation-derived p-values for Seed Circularity type 2 (BLUEs across trials). Only p-values originally below 10^{-4} in any of the three GWAS methods (FarmCPU, GEMMA, ISIS EM-BLASSO) are shown. Both p-value sets are -log10 transformed. To the points is fitted a smooth spline constrained to be monotonously increasing. 4 p-value thresholds are shown as dashed lines, the yellow and red horizontal ones are the 0.01 and 0.05 thresholds estimated using simpleM, while the green and blue vertical ones are the 0.01 and 0.05 thresholds estimated using PermGWAS2.

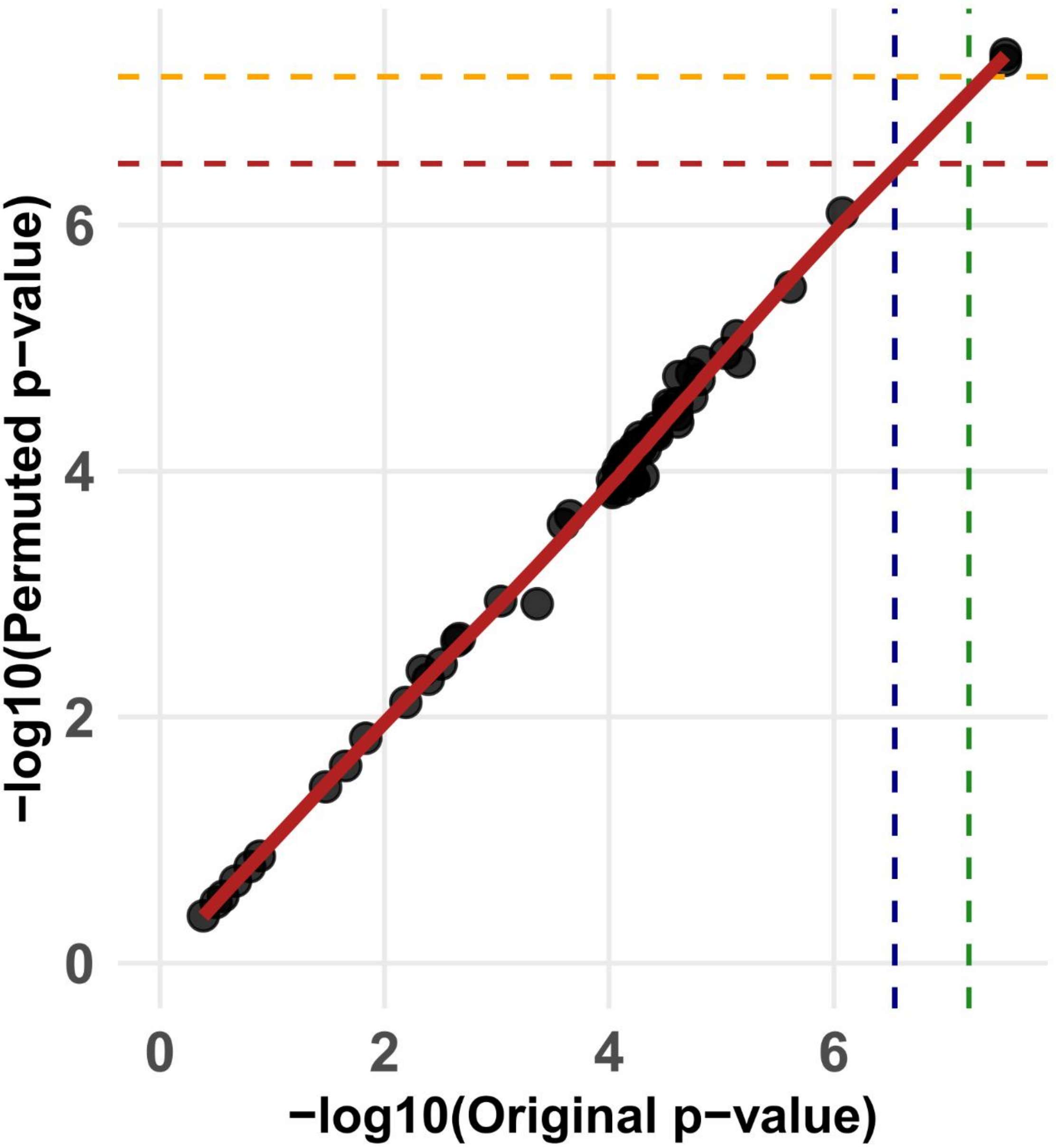

Extended data figure 55: Scatterplot of GEMMA parametric p-values (unpermuted) and permutation-derived p-values for Seed Circularity type 2 in Sejet 2020. Only p-values originally below 10^{-4} in any of the three GWAS methods (FarmCPU, GEMMA, ISIS EM-BLASSO) are shown. Both p-value sets are -log10 transformed. To the points is fitted a smooth spline constrained to be monotonously increasing. 4 p-value thresholds are shown as dashed lines, the yellow and red horizontal ones are the 0.01 and 0.05 thresholds estimated using simpleM, while the green and blue vertical ones are the 0.01 and 0.05 thresholds estimated using PermGWAS2.

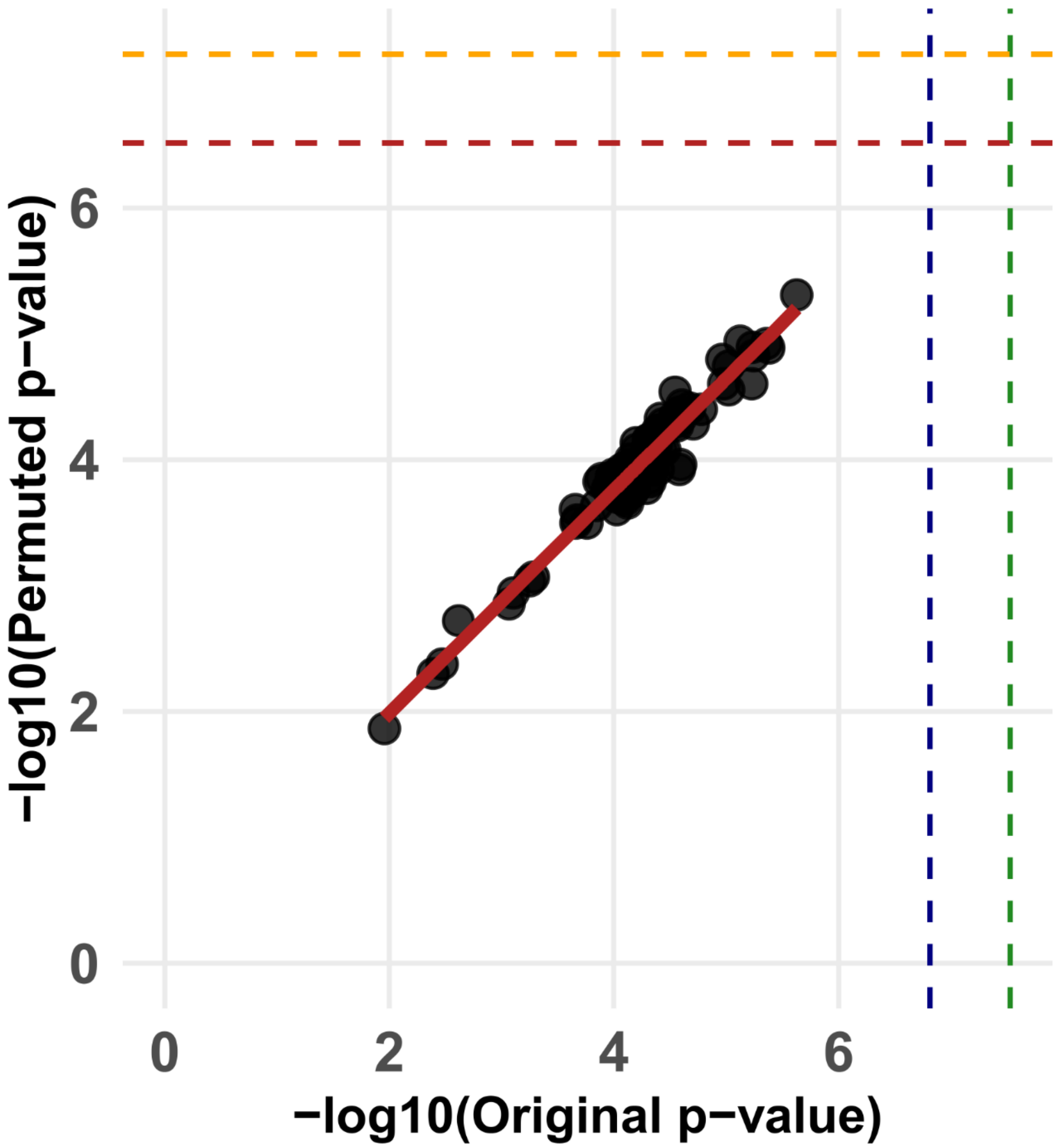

Extended data figure 56: Scatterplot of GEMMA parametric p-values (unpermuted) and permutation-derived p-values for Seed Circularity type 2 in Sejet 2021. Only p-values originally below 10^{-4} in any of the three GWAS methods (FarmCPU, GEMMA, ISIS EM-BLASSO) are shown. Both p-value sets are -log10 transformed. To the points is fitted a smooth spline constrained to be monotonously increasing. 4 p-value thresholds are shown as dashed lines, the yellow and red horizontal ones are the 0.01 and 0.05 thresholds estimated using simpleM, while the green and blue vertical ones are the 0.01 and 0.05 thresholds estimated using PermGWAS2.

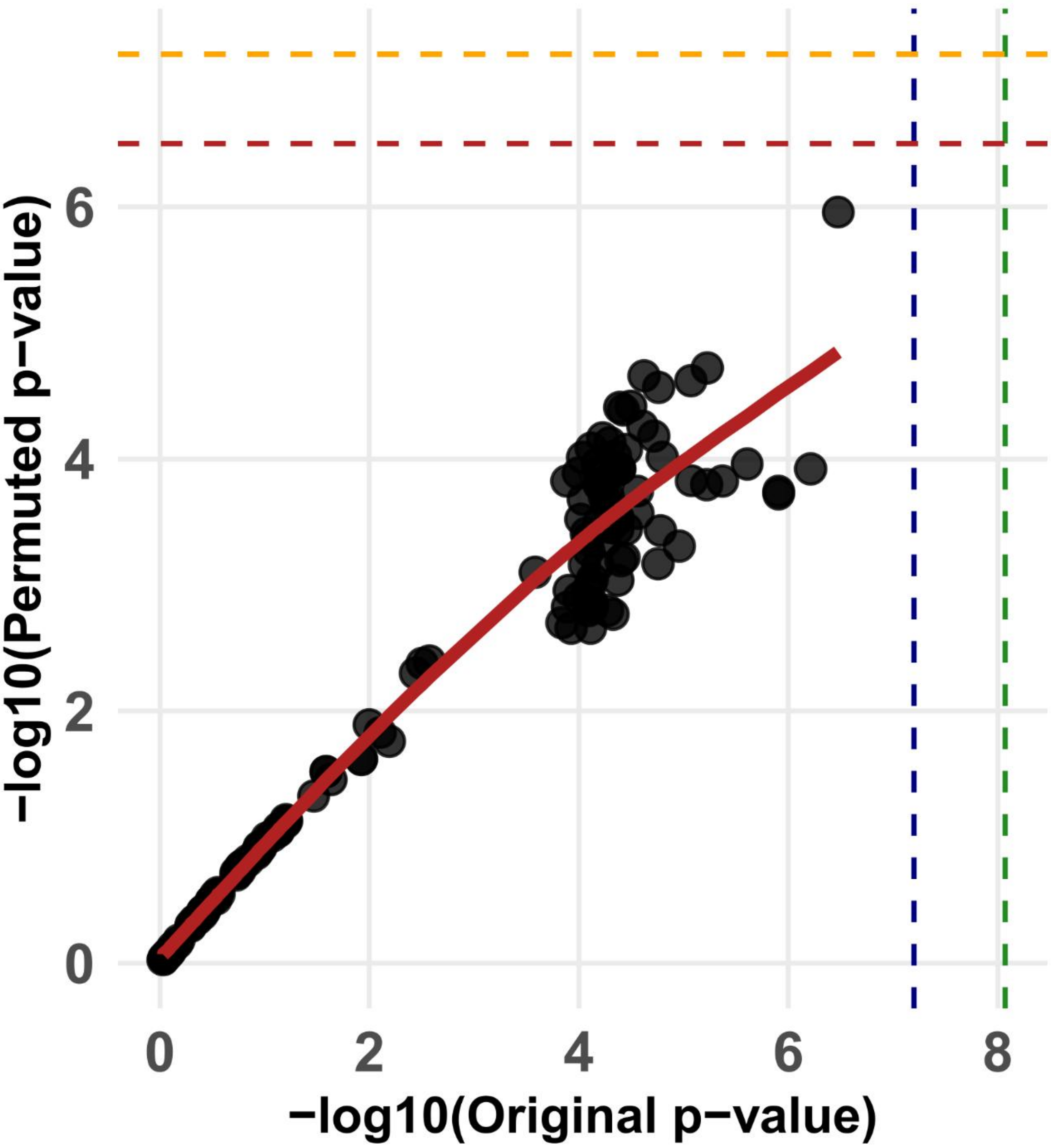

Extended data figure 57: Scatterplot of GEMMA parametric p-values (unpermuted) and permutation-derived p-values for Seed L to W ratio type 1 in Sejet 2019. Only p-values originally below 10^{-4} in any of the three GWAS methods (FarmCPU, GEMMA, ISIS EM-BLASSO) are shown. Both p-value sets are -log10 transformed. To the points is fitted a smooth spline constrained to be monotonously increasing. 4 p-value thresholds are shown as dashed lines, the yellow and red horizontal ones are the 0.01 and 0.05 thresholds estimated using simpleM, while the green and blue vertical ones are the 0.01 and 0.05 thresholds estimated using PermGWAS2.

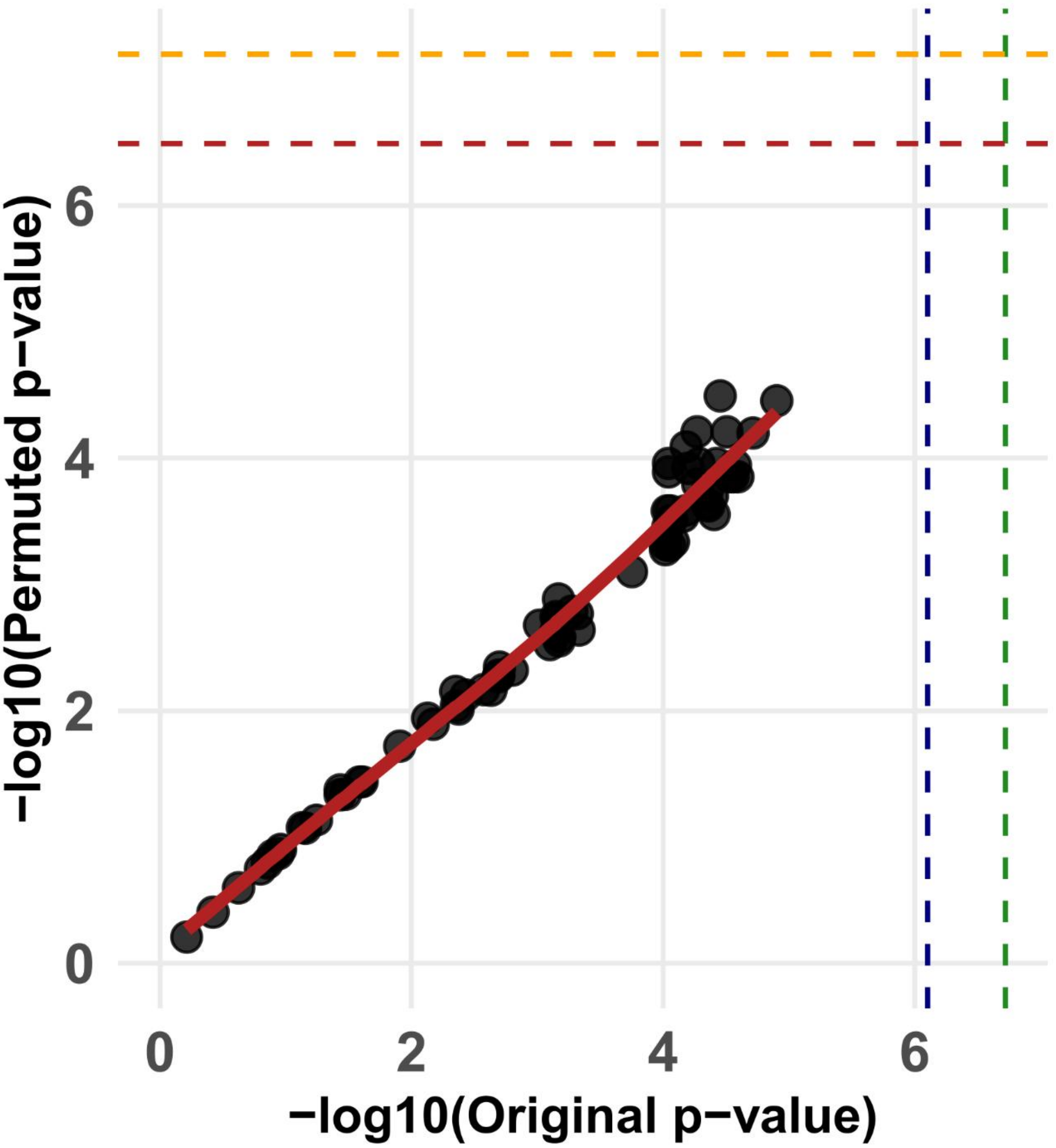

Extended data figure 58: Scatterplot of GEMMA parametric p-values (unpermuted) and permutation-derived p-values for Seed L to W ratio type 2 (BLUEs across trials). Only p-values originally below 10^{-4} in any of the three GWAS methods (FarmCPU, GEMMA, ISIS EM-BLASSO) are shown. Both p-value sets are -log10 transformed. To the points is fitted a smooth spline constrained to be monotonously increasing. 4 p-value thresholds are shown as dashed lines, the yellow and red horizontal ones are the 0.01 and 0.05 thresholds estimated using simpleM, while the green and blue vertical ones are the 0.01 and 0.05 thresholds estimated using PermGWAS2.

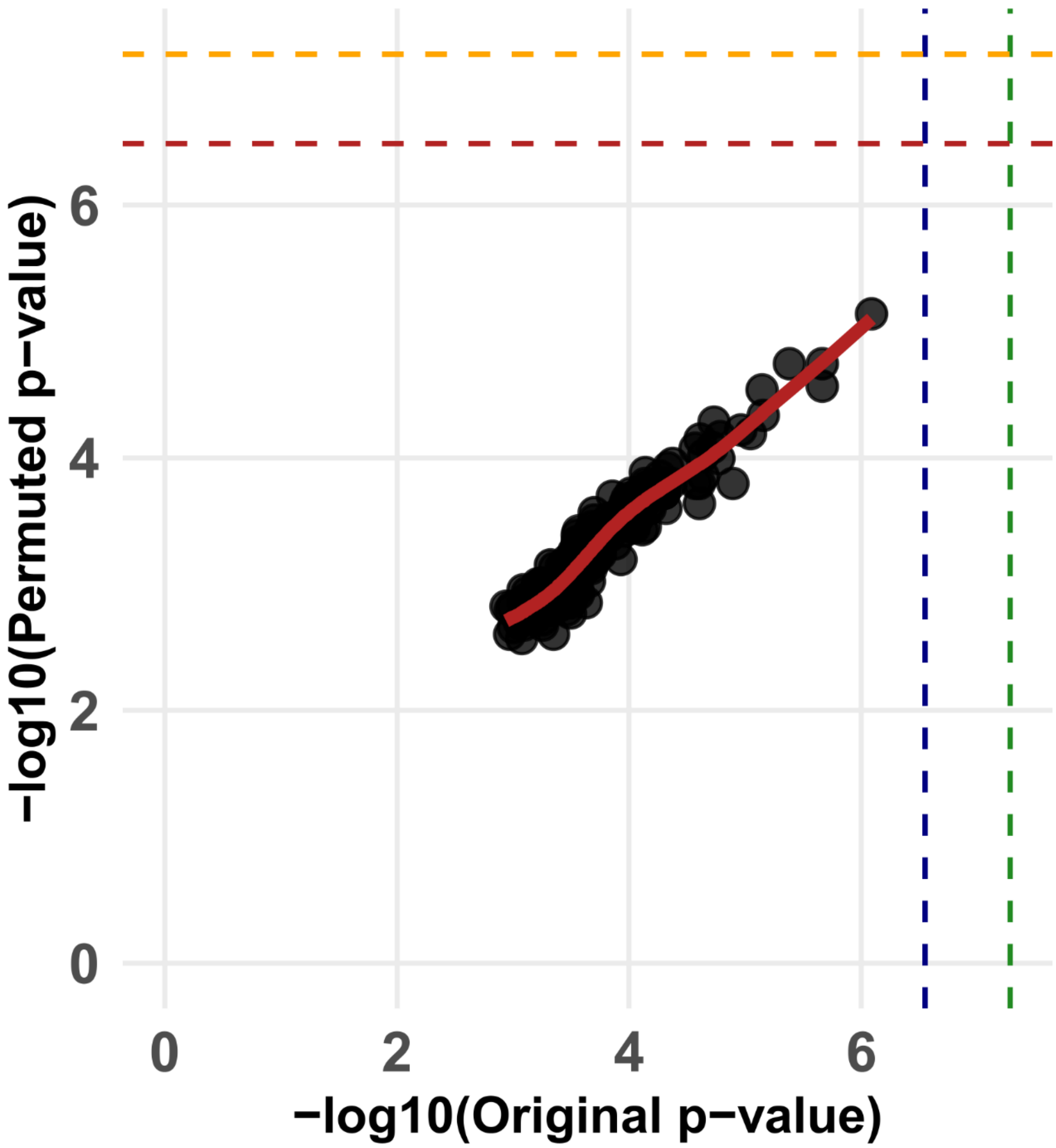

Extended data figure 59: Scatterplot of GEMMA parametric p-values (unpermuted) and permutation-derived p-values for Seed L to W ratio type 2 in Nordic Seed 2018. Only p-values originally below 10^{-4} in any of the three GWAS methods (FarmCPU, GEMMA, ISIS EM-BLASSO) are shown. Both p-value sets are -log10 transformed. To the points is fitted a smooth spline constrained to be monotonously increasing. 4 p-value thresholds are shown as dashed lines, the yellow and red horizontal ones are the 0.01 and 0.05 thresholds estimated using simpleM, while the green and blue vertical ones are the 0.01 and 0.05 thresholds estimated using PermGWAS2.

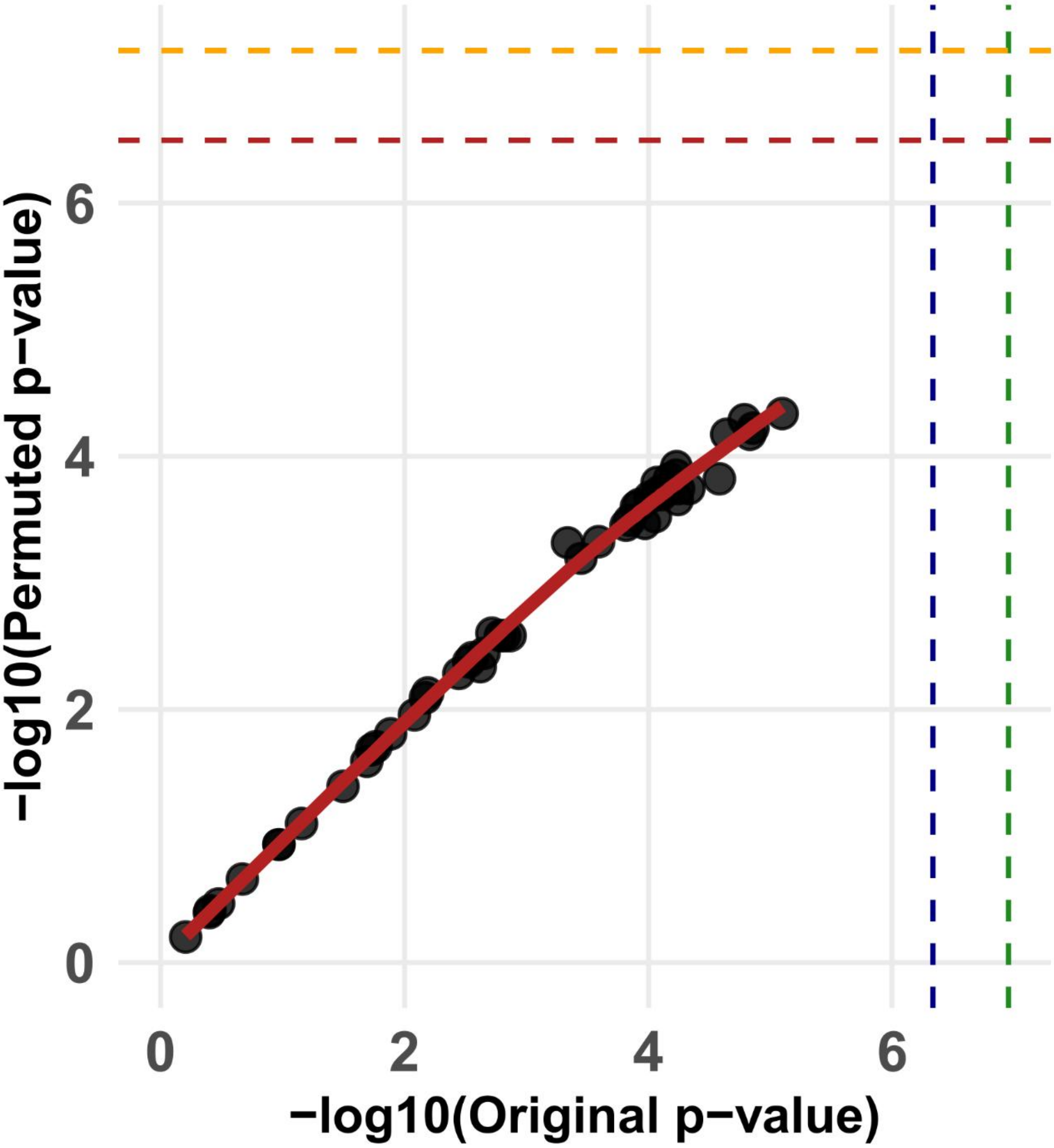

Extended data figure 60: Scatterplot of GEMMA parametric p-values (unpermuted) and permutation-derived p-values for Seed L to W ratio type 2 in Nordic Seed 2020. Only p-values originally below 10^{-4} in any of the three GWAS methods (FarmCPU, GEMMA, ISIS EM-BLASSO) are shown. Both p-value sets are -log10 transformed. To the points is fitted a smooth spline constrained to be monotonously increasing. 4 p-value thresholds are shown as dashed lines, the yellow and red horizontal ones are the 0.01 and 0.05 thresholds estimated using simpleM, while the green and blue vertical ones are the 0.01 and 0.05 thresholds estimated using PermGWAS2.

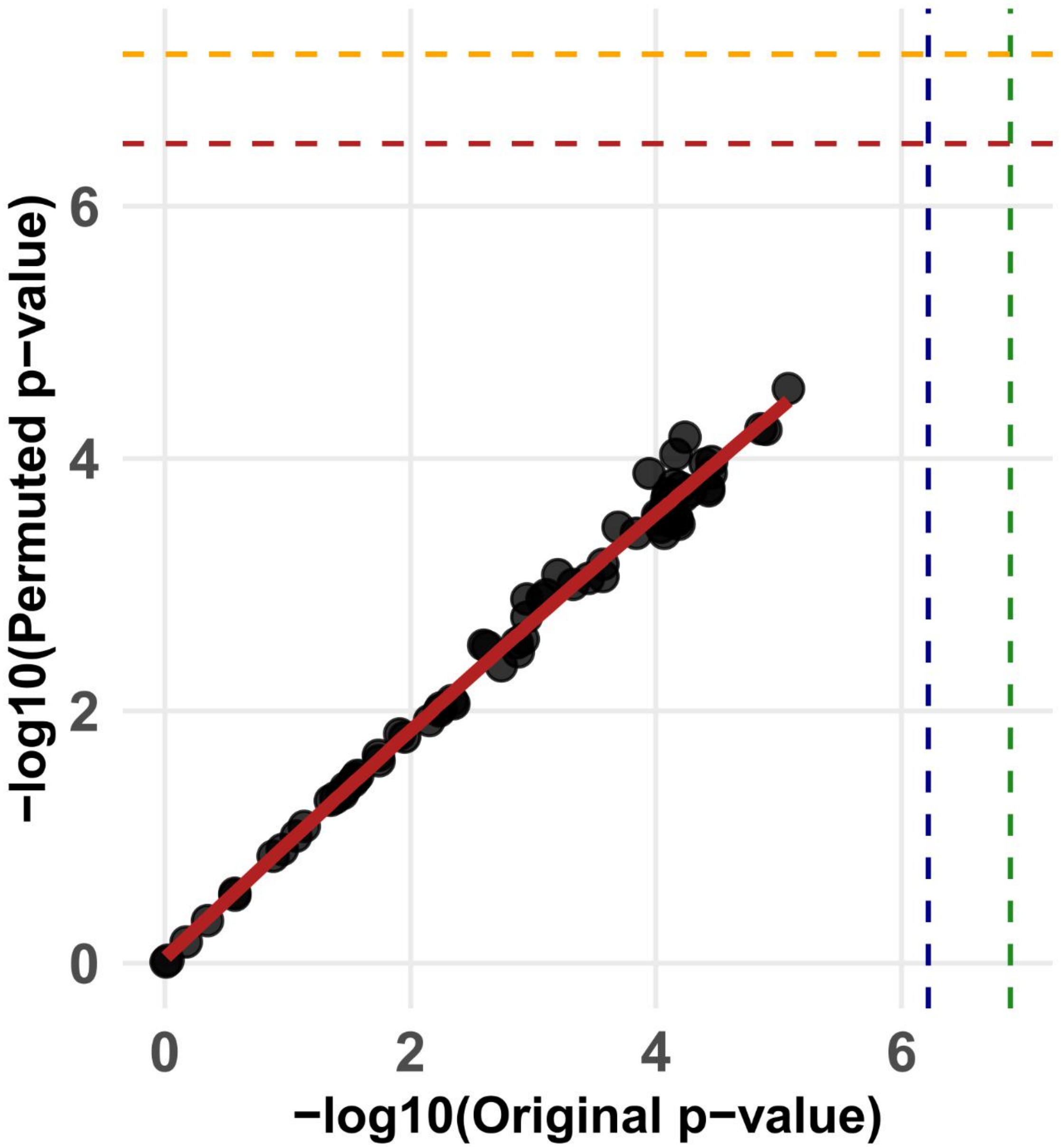

Extended data figure 61: Scatterplot of GEMMA parametric p-values (unpermuted) and permutation-derived p-values for Seed L to W ratio type 2 in Sejet 2020. Only p-values originally below 10^{-4} in any of the three GWAS methods (FarmCPU, GEMMA, ISIS EM-BLASSO) are shown. Both p-value sets are -log10 transformed. To the points is fitted a smooth spline constrained to be monotonously increasing. 4 p-value thresholds are shown as dashed lines, the yellow and red horizontal ones are the 0.01 and 0.05 thresholds estimated using simpleM, while the green and blue vertical ones are the 0.01 and 0.05 thresholds estimated using PermGWAS2.

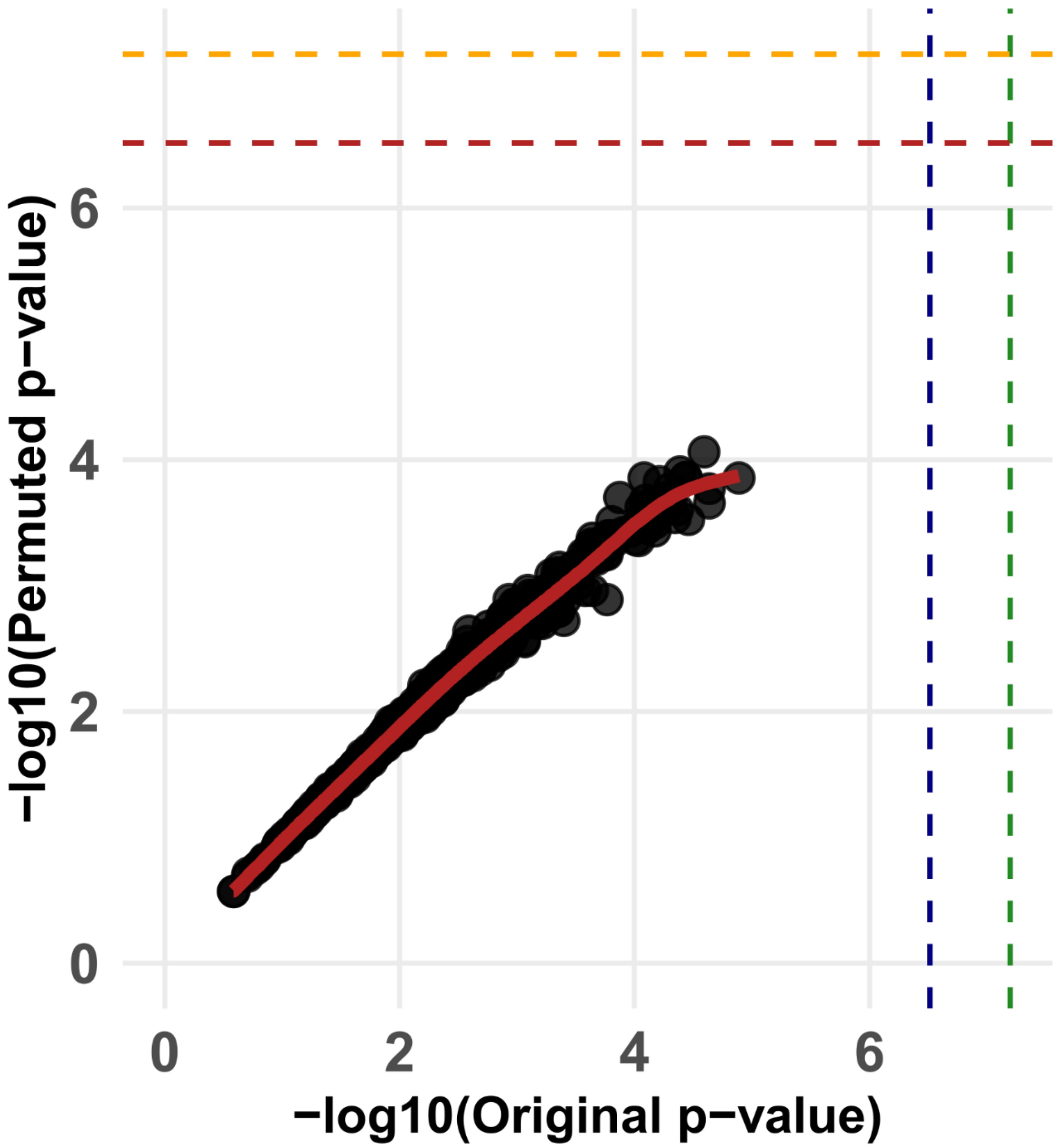

Extended data figure 62: Scatterplot of GEMMA parametric p-values (unpermuted) and permutation-derived p-values for Seed L to W ratio type 2 in Sejet 2021. Only p-values originally below 10^{-4} in any of the three GWAS methods (FarmCPU, GEMMA, ISIS EM-BLASSO) are shown. Both p-value sets are -log10 transformed. To the points is fitted a smooth spline constrained to be monotonously increasing. 4 p-value thresholds are shown as dashed lines, the yellow and red horizontal ones are the 0.01 and 0.05 thresholds estimated using simpleM, while the green and blue vertical ones are the 0.01 and 0.05 thresholds estimated using PermGWAS2.

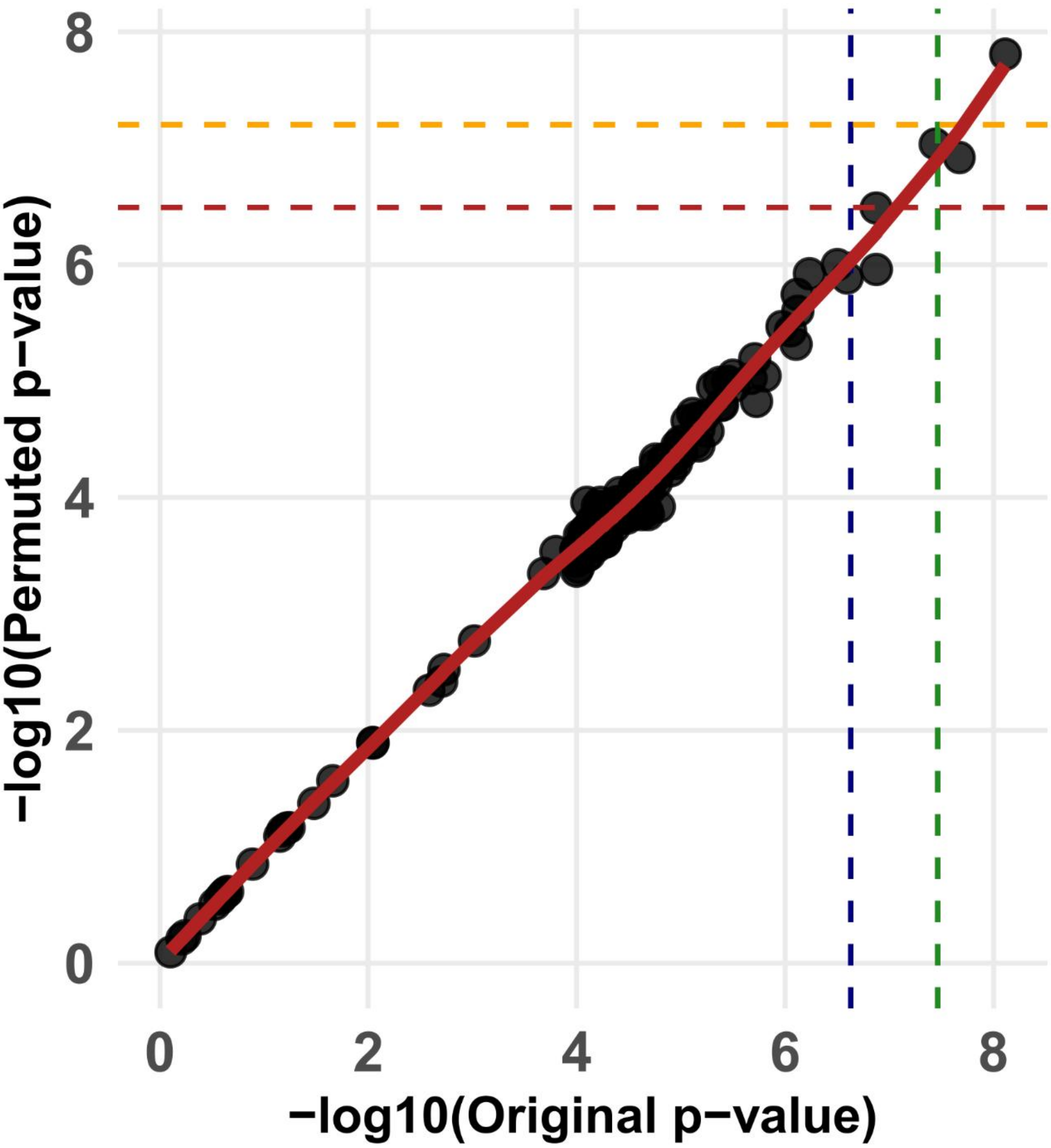

Extended data figure 63: Scatterplot of GEMMA parametric p-values (unpermuted) and permutation-derived p-values for Sterile Tillers (BLUEs across trials). Only p-values originally below 10^{-4} in any of the three GWAS methods (FarmCPU, GEMMA, ISIS EM-BLASSO) are shown. Both p-value sets are -log10 transformed. To the points is fitted a smooth spline constrained to be monotonously increasing. 4 p-value thresholds are shown as dashed lines, the yellow and red horizontal ones are the 0.01 and 0.05 thresholds estimated using simpleM, while the green and blue vertical ones are the 0.01 and 0.05 thresholds estimated using PermGWAS2.

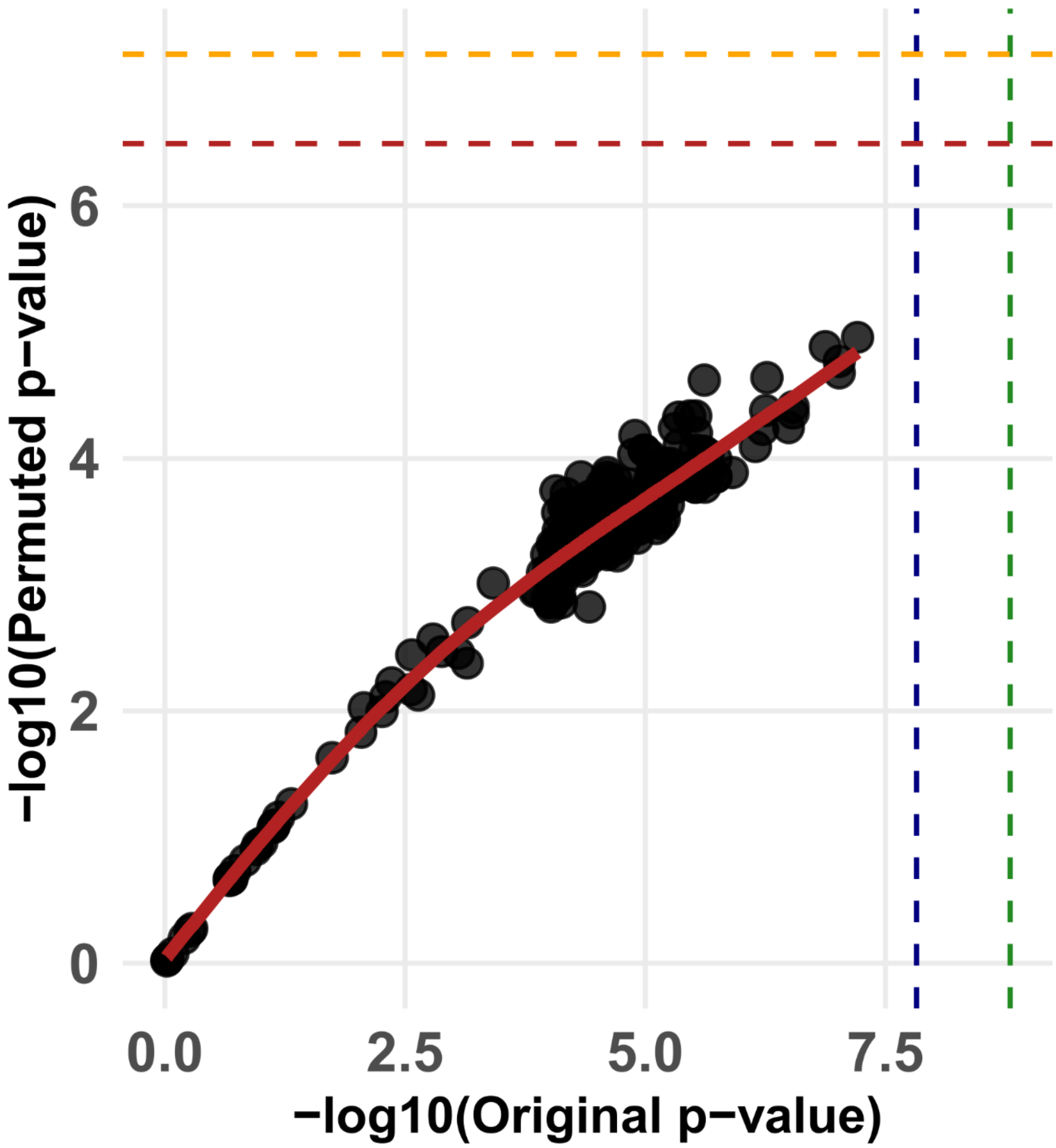

Extended data figure 64: Scatterplot of GEMMA parametric p-values (unpermuted) and permutation-derived p-values for Sterile Tillers in Nordic Seed 2019. Only p-values originally below 10^{-4} in any of the three GWAS methods (FarmCPU, GEMMA, ISIS EM-BLASSO) are shown. Both p-value sets are -log10 transformed. To the points is fitted a smooth spline constrained to be monotonously increasing. 4 p-value thresholds are shown as dashed lines, the yellow and red horizontal ones are the 0.01 and 0.05 thresholds estimated using simpleM, while the green and blue vertical ones are the 0.01 and 0.05 thresholds estimated using PermGWAS2.

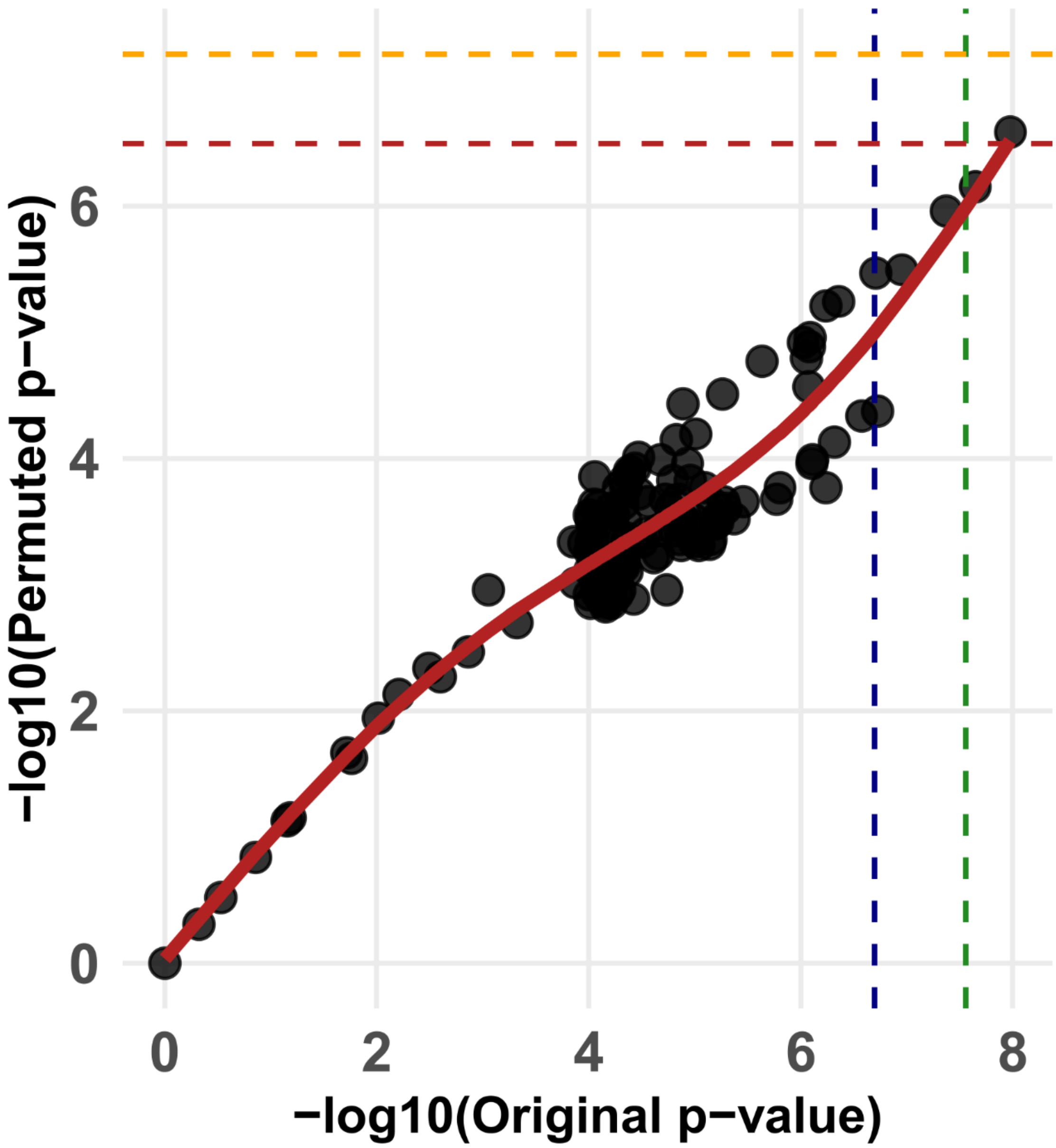

Extended data figure 65: Scatterplot of GEMMA parametric p-values (unpermuted) and permutation-derived p-values for Sterile Tillers in Nordic Seed 2020. Only p-values originally below 10^{-4} in any of the three GWAS methods (FarmCPU, GEMMA, ISIS EM-BLASSO) are shown. Both p-value sets are -log10 transformed. To the points is fitted a smooth spline constrained to be monotonously increasing. 4 p-value thresholds are shown as dashed lines, the yellow and red horizontal ones are the 0.01 and 0.05 thresholds estimated using simpleM, while the green and blue vertical ones are the 0.01 and 0.05 thresholds estimated using PermGWAS2.
